# Individual participant data meta-analysis to compare EPDS accuracy to detect major depression with and without the self-harm item

**DOI:** 10.1038/s41598-023-29114-w

**Published:** 2023-03-10

**Authors:** Xia Qiu, Yin Wu, Ying Sun, Brooke Levis, Jizhou Tian, Jill T. Boruff, Pim Cuijpers, John P. A. Ioannidis, Sarah Markham, Roy C. Ziegelstein, Simone N. Vigod, Andrea Benedetti, Brett D. Thombs, the DEPRESsion Screening Data (DEPRESSD) EPDS Group, Chen He, Ankur Krishnan, Parash Mani Bhandari, Dipika Neupane, Zelalem Negeri, Mahrukh Imran, Danielle B. Rice, Marleine Azar, Matthew J. Chiovitti, Simon Gilbody, Lorie A. Kloda, Scott B. Patten, Nicholas D. Mitchell, Rubén Alvarado, Jacqueline Barnes, Cheryl Tatano Beck, Carola Bindt, Humberto Correa, Tiago Castro e Couto, Genesis Chorwe-Sungani, Valsamma Eapen, Nicolas Favez, Ethel Felice, Gracia Fellmeth, Michelle Fernandes, Sally Field, Barbara Figueiredo, Jane R. W. Fisher, Eric P. Green, Simone Honikman, Louise M. Howard, Pirjo A. Kettunen, Jane Kohlhoff, Zoltán Kozinszky, Angeliki A. Leonardou, Michael Maes, Pablo Martínez, Sandra Nakić Radoš, Daisuke Nishi, Susan J. Pawlby, Tamsen J. Rochat, Heather J. Rowe, Deborah J. Sharp, Alkistis Skalkidou, Johanne Smith-Nielsen, Alan Stein, Kuan-Pin Su, Inger Sundström-Poromaa, Meri Tadinac, S. Darius Tandon, Iva Tendais, Annamária Töreki, Thach D. Tran, Kylee Trevillion, Katherine Turner, Mette S. Væver, Thandi van Heyningen, Johann M. Vega-Dienstmaier, Karen Wynter, Kimberly A. Yonkers

**Affiliations:** 1 Lady Davis Institute for Medical Research, Jewish General Hospital, 4333 Cote Ste Catherine Road, Montréal, QC H3T 1E4 Canada; 2 Department of Pediatrics, West China Second University Hospital, Key Laboratory of Obstetric and Gynecologic and Pediatric Diseases and Birth Defects of Ministry of Education, Sichuan University, Chengdu, Sichuan China; 3 Department of Psychiatry, McGill University, Montréal, QC Canada; 4 Centre for Prognosis Research, School of Medicine, Keele University, Staffordshire, UK; 5 Schulich Library of Physical Sciences, Life Sciences, and Engineering, McGill University, Montréal, QC Canada; 6 Department of Clinical, Neuro and Developmental Psychology, Amsterdam Public Health Research Institute, Vrije Universiteit, Amsterdam, the Netherlands; 7 Department of Medicine, Department of Epidemiology and Population Health, Department of Biomedical Data Science, Department of Statistics, Stanford University, Stanford, CA USA; 8 Department of Biostatistics and Health Informatics, King’s College London, London, UK; 9 Department of Medicine, Johns Hopkins University School of Medicine, Baltimore, MD USA; 10 Women’s College Hospital and Research Institute, University of Toronto, Toronto, ON Canada; 11 Department of Epidemiology, Biostatistics and Occupational Health, McGill University, Montréal, QC Canada; 12 Respiratory Epidemiology and Clinical Research Unit, Centre for Outcomes Research and Evaluation, Research Institute of the McGill University Health Centre, 5252 Boulevard de Maisonneuve, Montréal, QC H4A 3S5 Canada; 13 Department of Medicine, McGill University, Montréal, QC Canada; 14 Department of Psychology, McGill University, Montréal, QC Canada; 15 Department of Educational and Counselling Psychology, McGill University, Montréal, QC Canada; 16 Biomedical Ethics Unit, McGill University, Montréal, QC Canada; 17 Hull York Medical School and the Department of Health Sciences, University of York, Heslington, York UK; 18 Concordia University, Montréal, QC Canada; 19 Departments of Community Health Sciences and Psychiatry, University of Calgary, Calgary, Canada; 20 Department of Psychiatry, University of Alberta, Edmonton, AB Canada; 21 School of Public Health, Faculty of Medicine, Universidad de Chile, Santiago, Chile; 22 Department of Psychological Sciences, Birkbeck, University of London, London, UK; 23 University of Connecticut School of Nursing, Mansfield, CT USA; 24 Department of Child and Adolescent Psychiatry, University Medical Center Hamburg-Eppendorf, Hamburg, Germany; 25 Faculty of Medicine, Universidade Federal de Minas Gerais, Belo Horizonte, MG Brazil; 26 Federal University of Uberlândia, Uberlândia, Brazil; 27 Department of Mental Health, Kamuzu College of Nursing, University of Malawi, Blantyre, Malawi; 28 School of Psychiatry, University of New South Wales, Kensington, Australia; 29 Faculty of Psychology and Educational Sciences, University of Geneva, Geneva, Switzerland; 30 Department of Psychiatry, Mount Carmel Hospital, Attard, Malta; 31 Nuffield Department of Population Health, University of Oxford, Oxford, UK; 32 Department of Paediatrics, MRC Lifecourse Epidemiology Centre and Faculty of Medicine, University of Southampton, Southampton, UK; 33 Perinatal Mental Health Project, Alan J. Flisher Centre for Public Mental Health, Department of Psychiatry and Mental Health, University of Cape Town, Cape Town, South Africa; 34 School of Psychology, University of Minho, Braga, Portugal; 35 School of Public Health and Preventive Medicine, Monash University, Melbourne, Australia; 36 Duke Global Health Institute, Durham, NC USA; 37 Institute of Psychiatry, Psychology and Neuroscience, King’s College London, London, UK; 38 Department of General Hospital Psychiatry, North Karelia Central Hospital, Joensuu, Finland; 39 Department of Obstetrics and Gynecology, Danderyd Hospital, Stockholm, Sweden; 40 First Department of Psychiatry, Women’s Mental Health Clinic, Athens University Medical School, Athens, Greece; 41 Department of Psychiatry, Faculty of Medicine, Chulalongkorn University, Bangkok, Thailand; 42 Escuela de Psicología, Facultad de Humanidades, Universidad de Santiago de Chile, Santiago, Chile; 43 Department of Psychology, Catholic University of Croatia, Zagreb, Croatia; 44 Department of Mental Health, Graduate School of Medicine, The University of Tokyo, Tokyo, Japan; 45 MRC/Developmental Pathways to Health Research Unit, Faculty of Health Sciences, University of Witwatersrand, Johannesburg, South Africa; 46 Centre for Academic Primary Care, Bristol Medical School, University of Bristol, Bristol, UK; 47 Department of Women’s and Children’s Health, Uppsala University, Uppsala, Sweden; 48 Department of Psychology, Center for Early Intervention and Family Studies, University of Copenhagen, Copenhagen, Denmark; 49 Department of Psychiatry, University of Oxford, Oxford, UK; 50 An-Nan Hospital, China Medical University and Mind-Body Interface Laboratory, China Medical University Hospital, Taichung, Taiwan; 51 Department of Psychology, Faculty of Humanities and Social Sciences, University of Zagreb, Zagreb, Croatia; 52 Feinberg School of Medicine, Northwestern University, Chicago, IL USA; 53 Department of Emergency, University of Szeged, Szeged, Hungary; 54 Epilepsy Center-Child Neuropsychiatry Unit, ASST Santi Paolo Carlo, San Paolo Hospital, Milan, Italy; 55 Facultad de Medicina Alberto Hurtado, Universidad Peruana Cayetano Heredia, Lima, Perú; 56 School of Nursing and Midwifery, Deakin University, Melbourne, Australia; 57 Department of Psychiatry, Yale School of Medicine, New Haven, CT USA

**Keywords:** Psychiatric disorders, Diagnosis, Psychology, Diseases, Health care, Medical research, Outcomes research

## Abstract

Item 10 of the Edinburgh Postnatal Depression Scale (EPDS) is intended to assess thoughts of intentional self-harm but may also elicit concerns about accidental self-harm. It does not specifically address suicide ideation but, nonetheless, is sometimes used as an indicator of suicidality. The 9-item version of the EPDS (EPDS-9), which omits item 10, is sometimes used in research due to concern about positive endorsements of item 10 and necessary follow-up. We assessed the equivalence of total score correlations and screening accuracy to detect major depression using the EPDS-9 versus full EPDS among pregnant and postpartum women. We searched Medline, Medline In-Process and Other Non-Indexed Citations, PsycINFO, and Web of Science from database inception to October 3, 2018 for studies that administered the EPDS and conducted diagnostic classification for major depression based on a validated semi-structured or fully structured interview among women aged 18 or older during pregnancy or within 12 months of giving birth. We conducted an individual participant data meta-analysis. We calculated Pearson correlations with 95% prediction interval (PI) between EPDS-9 and full EPDS total scores using a random effects model. Bivariate random-effects models were fitted to assess screening accuracy. Equivalence tests were done by comparing the confidence intervals (CIs) around the pooled sensitivity and specificity differences to the equivalence margin of δ = 0.05. Individual participant data were obtained from 41 eligible studies (10,906 participants, 1407 major depression cases). The correlation between EPDS-9 and full EPDS scores was 0.998 (95% PI 0.991, 0.999). For sensitivity, the EPDS-9 and full EPDS were equivalent for cut-offs 7–12 (difference range − 0.02, 0.01) and the equivalence was indeterminate for cut-offs 13–15 (all differences − 0.04). For specificity, the EPDS-9 and full EPDS were equivalent for all cut-offs (difference range 0.00, 0.01). The EPDS-9 performs similarly to the full EPDS and can be used when there are concerns about the implications of administering EPDS item 10.

**Trial registration:** The original IPDMA was registered in PROSPERO (CRD42015024785).

## Introduction

Depression is a common and disabling mental disorder among women during pregnancy and in the postpartum period^1,2^. A 2005 systematic review estimated that the point prevalence of major depression during pregnancy and postpartum ranged from 1 to 6% at different time points (first trimester of pregnancy to one year postpartum), based on 2–6 studies at any given time point (N = 111–2104 participants)^3^. A 2008 national survey from the USA with more than 14,000 participants reported 12-month period prevalence was similar among pregnant women (8%), postpartum women (9%), and similar-aged non-pregnant women (8%)^4^. Nonetheless, perinatal depression may have substantial adverse effects on mothers, fathers, partner relationships, and infants, including impairment of maternal function, paternal depression, premature delivery, infants with low birth weight and developmental delays, and impaired parent-infant interactions^5–8^.

Depression screening involves using self-report questionnaires to identify individuals who exceed a pre-defined cut-off score for further diagnostic evaluation to determine whether they have depression^9,10^. Guidelines from the United States Preventive Services Task Force and the Australian government recommend depression screening in pregnant and postpartum women^11,12^. The United Kingdom National Screening Committee and Canadian Task Force on Preventive Health Care, on the other hand, recommend against screening due to concerns about false positives, possible associated harms, and a lack of evidence from randomized controlled trials that screening leads to improved health outcomes^13,14^.

The 10‐item Edinburgh Postnatal Depression Scale (EPDS) is the most commonly used self‐report questionnaire for depression screening in pregnancy and postpartum^15,16^. It is also used to monitor symptoms among people undergoing treatment for depression and as a continuous outcome measure in research. Respondents rate how they have felt in the previous seven days^17^. Each item is scored 0–3, and possible total scores range from 0 to 30; higher scores indicate more severe depressive symptoms. Cut‐off values of ≥ 10 and ≥ 13 are often recommended for screening^15,18,19^. A 2020 individual participant data meta-analysis (IPDMA) reported that a cut-off of 11 or higher maximized the sum of sensitivity (81%) and specificity (88%), when using a semi-structured diagnostic interview as the reference standard (N = 36 studies, 9066 participants, 1330 major depression cases)^20^.

Although brief tools have been designed specifically to assess suicide ideation and risk in health care settings^21^, item 10 of the EPDS is sometimes used as a proxy of suicidal ideation. Item 10 of the EPDS is intended to assess thoughts of self-harm: “the thought of harming myself has occurred to me”^15,22^. A review from 2005 reported that 5–15% of pregnant and postpartum women had thoughts of self-harm (item score ≥ 1) based on this item^23^. However, responses to this item may not accurately reflect whether suicide ideation is present. One study compared positive responses on item 10 to item 3 of the Hamilton Depression Rating Scale (HDRS), which directly asks about suicidal ideation, among a sample of women with mood disorders during the first year postpartum; 17% (22/131) of participants who were administered the EPDS had positive responses versus 6% (9/146) on HDRS item 3^24^. A study of 574 pregnant and postpartum women with positive responses to item-10 found that 324 (57%) women had fleeting thoughts of avoiding problems but no intent to self-harm, and 75 (13%) misunderstood the item^25^. One potential reason for this is that the item is not specific; and some women may misinterpret it to include unintentional injury, such as due to falls from impaired balance^26–28^, for instance. When the full EPDS is used in research studies, misinterpretation of item 10 could require follow-up with many women who endorse the item, even though most responses are false positives. This consumes substantial resources without evidence of benefit to study participants from administering the item.

A 9-item version of the EPDS (EPDS-9), which omits item 10, is sometimes used. A study of 371 women from the United States referred to a program serving women with or at risk of postpartum depression found that 49% of participants scored ≥ 13 on the full EPDS compared to 48% based on the EPDS-9^29^. If differences in performance between the EPDS-9 and full EPDS are minimal, the EPDS-9 could be used for a range of purposes in research studies, where administering item 10 could have significant resource implications, including the need to follow-up on potentially large numbers of false positive responses to item 10. It might also be considered in trials of screening programs or in jurisdictions where screening is done in practice. However, no study has compared correlations between continuous scores and level of agreement in screening accuracy between the EPDS-9 and full EPDS. The objectives of the present study were to (1) evaluate the association of continuous EPDS-9 and full EPDS scores for assessing depressive symptom severity; and (2) assess the equivalence of the accuracy of the EPDS-9 and full EPDS across relevant cut-offs for screening to detect major depression.

## Methods

The present study used a subset of participants from a database originally synthesized for an IPDMA on the accuracy of the full EPDS for depression screening^20^. The original IPDMA was registered in PROSPERO (CRD42015024785), and a protocol was published^30^. Results from the main IPDMA of the EPDS have been published^20^. To assess the equivalence of the EPDS-9 and full EPDS, we followed similar methods to those used in our previously published study that assessed the equivalence of the Patient Health Questionnaire-8 (PHQ-8) and PHQ-9^31^. Prior to initiating the present study, we published a study-specific protocol on the Open Science Framework (https://osf.io/n9mfq/).

### Study eligibility

For the main IPDMA, studies and datasets were eligible if (1) they administered the EPDS; (2) diagnostic classification for current major depressive disorder or major depressive episode was done based on a validated semi-structured or fully structured interview using Diagnostic and Statistical Manual of Mental Disorders (DSM)^32–34^ or International Classification of Diseases (ICD) criteria^35^; (3) participants were women aged 18 or older who completed assessments during pregnancy or within 12 months of giving birth; (4) the EPDS and diagnostic interview were conducted within two weeks; and (5) participants were not limited to people receiving psychiatric assessment or seeking psychiatric care because screening is done to identify previously unrecognized cases. Datasets where not all participants were eligible were included if primary data allowed selection of eligible participants. There were no restrictions based on language or study design. For the present study, we only included datasets from primary studies that provided individual EPDS item scores for all 10 items, because only those datasets allowed us to generate EPDS-9 scores and compare the EPDS-9 and full EPDS.

### Search strategy and selection of eligible studies

A medical librarian searched Medline, Medline In-Process and Other Non-Indexed Citations, PsycINFO, and Web of Science from database inception to October 3, 2018, using a peer-reviewed search strategy (Supplementary Methods 1). Additionally, investigators reviewed reference lists of relevant reviews and queried contributing authors about non-published studies. Search results were uploaded into RefWorks (RefWorks-COS, Bethesda, MD, USA). After de-duplication, unique citations were uploaded into DistillerSR (Evidence Partners, Ottawa, Canada), which was used to store and track search results, conduct screening for eligibility, document correspondence with primary study authors, and extract study characteristics.

Two investigators independently reviewed titles and abstracts for eligibility. For publications deemed potentially eligible by either reviewer, a full-text review was done by two investigators, also independently. Disagreements between reviewers after full-text reviews were resolved by consensus and a third investigator was consulted if necessary.

### Data contribution, extraction, and synthesis

Authors of eligible datasets were invited to contribute de-identified primary data. We attempted to contact corresponding authors of eligible primary studies by email up to three times, as necessary. When authors did not respond to our emails, we tried to contact them by phone and emailed co-authors. There was no time limit for how long authors had to provide data.

Two investigators independently extracted information on the diagnostic interview administered and the country of study from the published reports. Any discrepancies were resolved by consensus. Participant-level data included in the synthesized dataset included country human development index (which reflects life expectancy, education, and income of a country)^36^, age, pregnant or postpartum status, diagnostic interview administered, major depression classification status, and EPDS item scores. We used major depressive disorder or major depressive episode based on DSM or ICD criteria; if both were reported, we prioritized major depressive episode because screening attempts to detect episodes of depression. Clinically, additional assessment would be needed to determine if episodes were related to major depressive disorder or another psychiatric disorder (bipolar disorder, persistent depressive disorder). We also prioritized DSM over ICD because DSM is more commonly used in existing studies. We used statistical weights to reflect sampling procedures if provided in the datasets, for instance, when primary studies administered a diagnostic interview to all participants with positive screening results but only a random sample of those with negative results. Some studies used sampling procedures that merited weights but did not use weights. For those studies, we used inverse selection probabilities to generate appropriate weights.

For all datasets, we verified that participant characteristics and screening accuracy results for the full EPDS matched those that had been published. When primary data and original publications were discrepant, we identified and corrected errors when possible and resolved any outstanding discrepancies in consultation with the original investigators. We transformed all study-level and individual-level participant data into a standardized format and combined in a single synthesized dataset. For nine studies that collected data at multiple time points (four with two time points, four with three time points, and one with four time points), we selected the time point with the most participants. If the number of participants was maximized at multiple time points, we selected the one with the most women who had major depression.

We used the Quality Assessment of Diagnostic Accuracy Studies-2 tool (QUADAS-2)^37^ to assess risk of bias of included studies. No QUADAS domain items, however, were associated with outcomes in our main EPDS IPDMA^20^. Furthermore, QUADAS is designed to assess risk of bias in estimates of screening accuracy but not study features that might bias differences between using a full scale and a minimally shortened version of that scale. Thus, QUADAS ratings are provided in Supplementary Methods 2 but were not include in analyses.

### Statistical analyses

To evaluate the association of EPDS-9 and full EPDS scores for assessing depressive symptom severity, a Pearson correlation and a 95% confidence interval (CI) were first calculated between the EPDS-9 and full EPDS scores for each study, then we generated the pooled estimate of correlations, 95% CI, and the prediction interval (PI), with a random effect model that accounted for clustering within primary studies.

To compare correlations and the screening accuracy of the EPDS-9 and full EPDS, we included all primary studies combined across type of diagnostic interview reference standards (primary analysis). There are differences in the way different types of diagnostic interviews are designed and their likelihood of classifying major depression^38–42^, but, since in each primary study, EPDS-9 and full EPDS scores are compared to the same reference standard, we did not have reason to believe that differences between the two measures would depend on the specific reference standard used. Nonetheless, we separately analyzed primary studies by the type of diagnostic interview used as the reference standard (secondary analyses), as we did in the previously published main EPDS IPDMA^20^.

For all studies pooled and by reference standard, for the EPDS-9 and full EPDS cut-offs ≥ 7 to ≥ 15, separately, bivariate random-effects models using an adaptive Gauss Hermite quadrature with 1 quadrature point^20,43^. This 2-stage meta-analytic approach models sensitivity and specificity at the same time, taking the inherent correlation between them and the precision of estimates within studies into account. A random-effects model was used as we assumed true values of sensitivity and specificity would vary across primary studies. We estimated accuracy for cut-offs from 7 to 15 to provide a range around the most commonly used cut-offs of ≥ 10 and ≥ 13, consistent with our main IPDMA of the EPDS^20^.

To examine the equivalence in accuracy between the EPDS-9 and full EPDS across cut-offs, overall and by reference standard, we used the results of the random-effects meta-analyses at each cut-off to construct separate empirical receiver operating characteristic (ROC) curves and areas under the curve (AUC) based on the pooled estimates. Equivalence between the EPDS-9 and full EPDS sensitivity and specificity was evaluated at each cut-off separately. This allowed us to test whether the sensitivity and specificity of the EPDS-9 were similar to the full EPDS, up to a pre-specified maximum difference, that is, an equivalence margin^44^. In the present study, an equivalence margin of δ = 0.05 was used, which is the same margin that was used previously to compare the PHQ-8 and PHQ-9^31^. CIs for the differences between the EPDS-9 and full EPDS sensitivity and specificity at each cut-off were constructed via a cluster bootstrap approach^45,46^, with resampling at the study and subject level. For each comparison, we ran 1000 iterations of the bootstrap. For each bootstrap iteration, the bivariate random-effects model was fitted to the EPDS-9 and full EPDS data, separately, and pooled sensitivities, specificities, and difference estimates between the EPDS-9 and full EPDS were computed. We compared the CIs around the pooled sensitivity and specificity differences to the equivalence margin of δ = 0.05. If the entire CI was between − 0.05 and + 0.05 then we rejected the hypothesis that there were differences large enough to be important and concluded that equivalence was present. If the entire CI was outside of the interval, then we failed to reject the hypothesis that the EPDS-9 and full EPDS were not equivalent. When the CIs crossed the ± 0.05 threshold, findings on equivalence were deemed indeterminate.

To investigate heterogeneity across studies, by overall and reference standard, we generated forest plots for the differences in sensitivity and specificity estimates between the EPDS-9 and full EPDS for cut-offs ≥ 10, ≥ 11 and ≥ 13 for each study. We also quantified heterogeneity at cut-offs ≥ 10, ≥ 11 and ≥ 13, by reporting the estimated variances of the random effects for the differences in the EPDS-9 and full EPDS sensitivity and specificity (τ^2^)^47,48^. Additionally, the 95% prediction intervals which we calculated reflect the range of true effects that can be expected in future settings or studies^49^.

All analyses were run in R software (R version R 3.5.0^50^ and R Studio version 1.1.423^51^ using the lme4 package^52^.

### Ethical approval

As this study involved secondary analysis of anonymized previously collected data, the Research Ethics Committee of the Jewish General Hospital determined that this project did not require research ethics approval. However, for each included dataset, we confirmed that the original study received ethics approval and that all patients provided informed consent.

## Results

### Search results and characteristics of the primary data

For the main IPDMA, 4434 unique titles and abstracts were identified from the electronic database searches. 4056 of these were excluded after title and abstract screening and 257 after full-text review (Supplementary Table S1), resulting in 121 eligible articles from 81 unique participant samples. Of these samples, 56 (69%) contributed datasets. Furthermore, authors of included studies contributed data from 2 unpublished studies. In total, 58 full EPDS studies were provided to the main IPDMA. For the present study, 17 studies (4626 participants, 659 major depression cases) with datasets that included full EPDS scores but not individual item scores were excluded. Thus, 41 studies (10,906 participants, 1407 major depression cases) were analyzed (Supplementary Figure S1). Characteristics of the included studies are shown in Supplementary Table S2. Characteristics of the 25 eligible studies that did not provide data and the 17 excluded studies that provided only EPDS total scores are shown in Supplementary Table S3.

There were 24 included primary studies (5412 participants, 803 major depression cases) that used semi-structured diagnostic interviews to assess major depression, 4 (3189 participants, 228 major depression cases) that used fully structured diagnostic interviews other than the Mini International Neuropsychiatric Interview (MINI), and 13 (2305 participants, 376 major depression cases) that used the MINI. The Structured Clinical Interview for DSM Disorders (SCID) was the most used semi-structured interview (22 studies, 5157 participants, 765 major depression cases), and the Composite International Diagnostic Interview was the most commonly used fully structured interview (3 studies, 2963 participants, 196 major depression cases). Characteristics of participants are shown in Table 1.

**Table 1:** Participant characteristics by subgroups.

Participant subgroup	N of participants	N (%) with major depression
All participants	10,906	1407 (13%)
Country human development index		
Very high	7741	940 (12%)
High	1780	235 (13%)
Low-medium	1385	232 (17%)
Age		
< 25	2549	402 (16%)
≥ 25	8342	1003 (12%)
Not reported	15	2 (13%)
Pregnant or postpartum status		
Pregnant	6043	756 (13%)
Postpartum	4863	651 (13%)
Type of diagnostic interview		
Semi-structured diagnostic interview	5412	803 (15%)
Fully structured diagnostic interview	3189	228 (7%)
Mini International Neuropsychiatric Interview	2305	376 (16%)

### EPDS-9 and item 10 scores

As shown in Table 2, among participants in all studies, 1% of participants screened negative at an EPDS-9 cut-off of ≥ 10 but had a non-zero EPDS item 10 score. This percentage was also 1% at a cut-off of ≥ 11 and increased to 2% at a cut-off of ≥ 13. The correlation between the EPDS-9 and full EPDS scores was 0.998 (95% PI: 0.991, 0.999). The forest plot is shown in Supplementary Figure S2.

**Table 2:** Characteristics of participants who rated item 10 by EPDS-9 score at cut-offs ≥ 10, ≥ 11, and ≥ 13.

Cut-off	N of participants^a^ (N studies)	N (%) with positive EPDS-9 score	N (%) with positive full EPDS score	N (%) with negative screening result for EPDS-9 score and non-zero item 10 score
All studies				
≥ 10	29,216 (41)	6204 (21%)	6268 (22%)	285 (1%)
≥ 11	29,216 (41)	5166 (18%)	5277 (18%)	374 (1%)
≥ 13	29,216 (41)	3364 (12%)	3486 (12%)	549 (2%)
Semi-structured reference standard				
≥ 10	19,285 (24)	4021 (21%)	4058 (21%)	161 (1%)
≥ 11	19,285 (24)	3367 (18%)	3445 (18%)	222 (1%)
≥ 13	19,285 (24)	2167 (11%)	2227 (12%)	301 (2%)
Fully structured reference standard				
≥ 10	5360 (4)	1091 (20%)	1097 (21%)	28 (1%)
≥ 11	5360 (4)	892 (17%)	898 (17%)	35 (1%)
≥ 13	5360 (4)	602 (11%)	610 (11%)	47 (1%)
MINI reference standard				
≥ 10	4571 (13)	1092 (24%)	1112 (24%)	96 (2%)
≥ 11	4571 (13)	907 (20%)	934 (20%)	117 (3%)
≥ 13	4571 (13)	596 (13%)	648 (14%)	201 (4%)

EPDS, Edinburgh Postnatal Depression Scale.

^a^Numbers of participants add up to > 10,906 as they were weighted by sampling weights.

### Screening accuracy of the EPDS-9 and full EPDS

**Figure 1:**
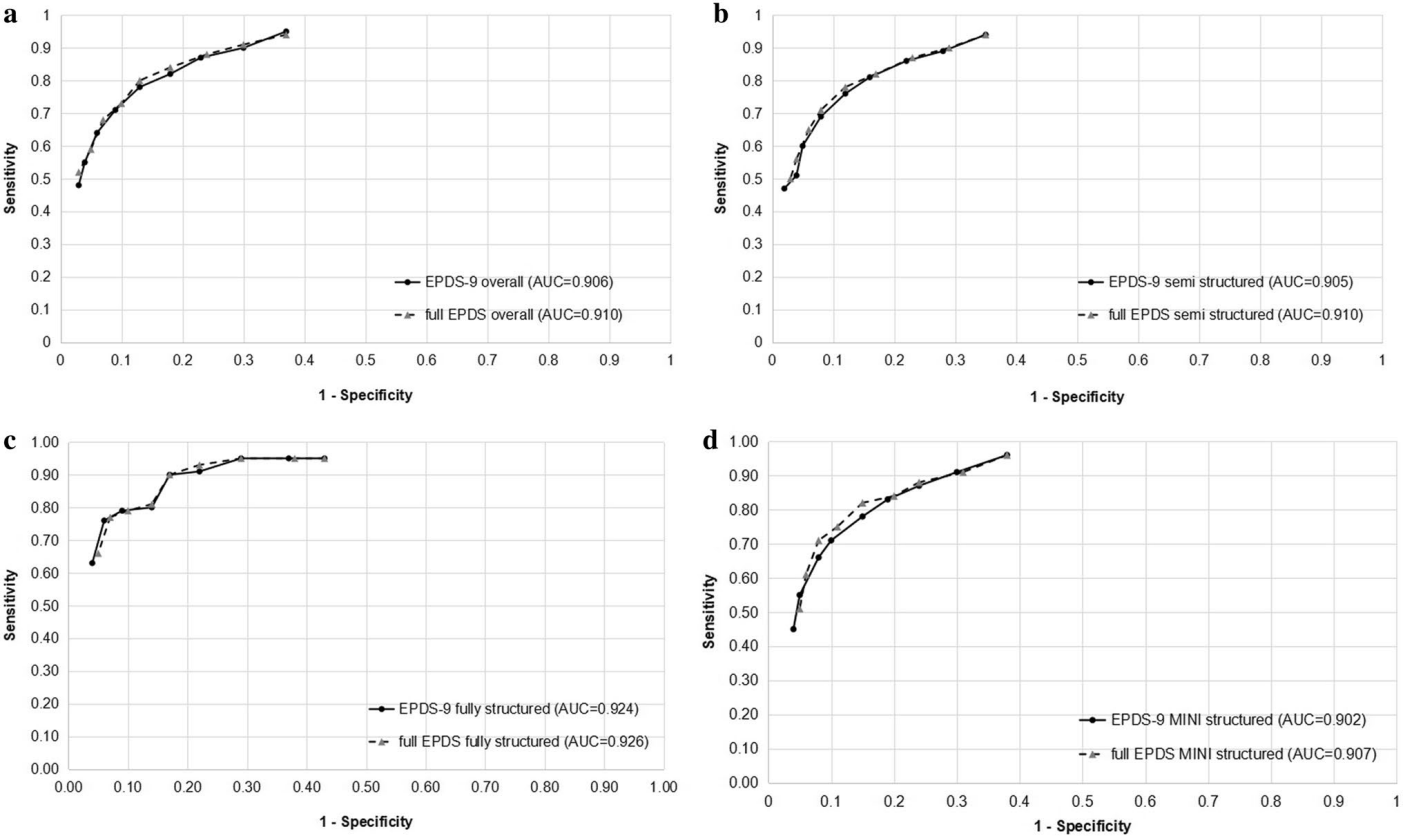
(**a**)–(**d**) ROC curves for the EPDS-9 and full EPDS (**a**) compared to all reference standards, (**b**) compared to a semi-structured reference standard, (**c**) compared to a fully structured reference standard (MINI excluded), and (**d**) compared to the MINI reference standard.

ROC curves that compare sensitivity and specificity estimates of the EPDS-9 and full EPDS for cut-offs ≥ 7 to ≥ 15 are shown in Fig. 1, overall and separately by semi-structured, fully structured, and MINI reference standards. The ROC curves for the EPDS-9 and full EPDS were highly overlapping for overall and each reference standard. The AUC of the EPDS-9 and full EPDS for all interviews combined was 0.906 versus 0.910. By interview type, it was 0.905 versus 0.910 for semi-structured interviews, 0.924 versus 0.926 for fully structured interviews (excluding the MINI), and 0.902 versus 0.907 for the MINI.

Comparisons of sensitivity and specificity estimates between the EPDS-9 and full EPDS at cut-offs ≥ 7 to ≥ 15 for all reference standards combined are shown in Table 3. At cut-off ≥ 11, which maximized the sum of sensitivity and specificity of the full EPDS in the main IPDMA^20^, sensitivity was 0.78 (95% CI 0.71, 0.84) and specificity was 0.87 (95% CI 0.83, 0.90) for the EPDS-9 versus 0.80 (95% CI 0.74, 0.86) and 0.87 (95% CI 0.83, 0.90) for the full EPDS. Comparisons of sensitivity and specificity estimates between the EPDS-9 and full EPDS for cut-offs ≥ 7 to ≥ 15 across the three different reference standard categories are shown in Supplementary Table S4.

**Table 3:** Comparison of sensitivity and specificity estimates between EPDS-9 and full EPDS across cut-offs 7–15 for studies that used all reference standards (N Studies = 41; N Participants = 10,906; N major depression = 1407).

Cut-off	EPDS-9^a^				Full EPDS^b^				EPDS-9 – full EPDS			
	Sensitivity	95% CI	Specificity	95% CI	Sensitivity	95% CI	Specificity	95% CI	Sensitivity	95% CI	Specificity	95% CI
≥ 7	0.95	(0.91, 0.97)	0.63	(0.57, 0.69)	0.94	(0.91, 0.97)	0.63	(0.56, 0.69)	0.01	(− 0.00, 0.00)	0.00	(0.00, 0.00)
≥ 8	0.90	(0.86, 0.93)	0.70	(0.64, 0.76)	0.91	(0.86, 0.94)	0.70	(0.64, 0.75)	− 0.01	(− 0.02, − 0.00)	0.00	(0.00, 0.01)
≥ 9	0.87	(0.82, 0.91)	0.77	(0.71, 0.82)	0.88	(0.83, 0.92)	0.76	(0.71, 0.81)	− 0.01	(− 0.03, 0.00)	0.01	(0.00, 0.01)
≥ 10	0.82	(0.76, 0.87)	0.82	(0.78, 0.86)	0.84	(0.78, 0.89)	0.82	(0.77, 0.86)	− 0.02	(− 0.04, − 0.00)	0.00	(0.00, 0.01)
≥ 11	0.78	(0.71, 0.84)	0.87	(0.83, 0.90)	0.80	(0.74, 0.86)	0.87	(0.83, 0.90)	− 0.02	(− 0.04, − 0.01)	0.00	(0.00, 0.01)
≥ 12	0.71	(0.63, 0.78)	0.91	(0.88, 0.93)	0.73	(0.65, 0.80)	0.90	(0.87, 0.93)	− 0.02	(− 0.05, − 0.01)	0.01	(0.00, 0.01)
≥ 13	0.64	(0.55, 0.72)	0.94	(0.91, 0.95)	0.68	(0.59, 0.76)	0.93	(0.90, 0.95)	− 0.04	(− 0.08, − 0.02)	0.01	(0.00, 0.01)
≥ 14	0.55	(0.46, 0.63)	0.96	(0.94, 0.97)	0.59	(0.50, 0.68)	0.95	(0.93, 0.97)	− 0.04	(− 0.08, − 0.02)	0.01	(0.00, 0.01)
≥ 15^a,b^	0.48	(0.40, 0.56)	0.97	(0.96, 0.98)	0.52	(0.44, 0.60)	0.97	(0.95, 0.98)	− 0.04	(− 0.07, − 0.02)	0.00	(0.00, 0.01)

CI, confidence interval; EPDS, Edinburgh Postnatal Depression Scale.

^a^For EPDS-9 cut-off 15, among all studies, the default and bobyqa optimizers in glmer failed to converge, thus L-BFSG-B was used instead.

^b^For full EPDS cut-off 15, among all studies, the default optimizer in glmer failed to converge, thus bobyqa was used instead.

Overall, among all 41 primary studies, across cut-offs ≥ 7 to ≥ 15, sensitivity was between 1 percent higher and 4 percent lower for the EPDS-9 compared to the full EPDS (Table 3). At cut-off ≥ 10, the difference was − 0.02 (95% CI − 0.04, − 0.00), at cut-off ≥ 11, the difference was − 0.02 (95% CI − 0.04, − 0.01), and at cut-off ≥ 13, the difference was − 0.04 (95% CI − 0.08, − 0.02). The EPDS-9 and full EPDS were equivalent for cut-offs ≥ 7 to ≥ 12 and the equivalence was indeterminate for cut-offs ≥ 13 to ≥ 15. For specificity, the differences between the EPDS-9 and full EPDS were within 0.01 for all cut-offs. The EPDS-9 and full EPDS were equivalent for all cut-offs. As shown in Supplementary Table S4, in comparisons stratified by different reference standards, sensitivity estimates were similarly equivalent or indeterminate, and specificity estimates were equivalent at all cut-offs.

**Figure 2:**
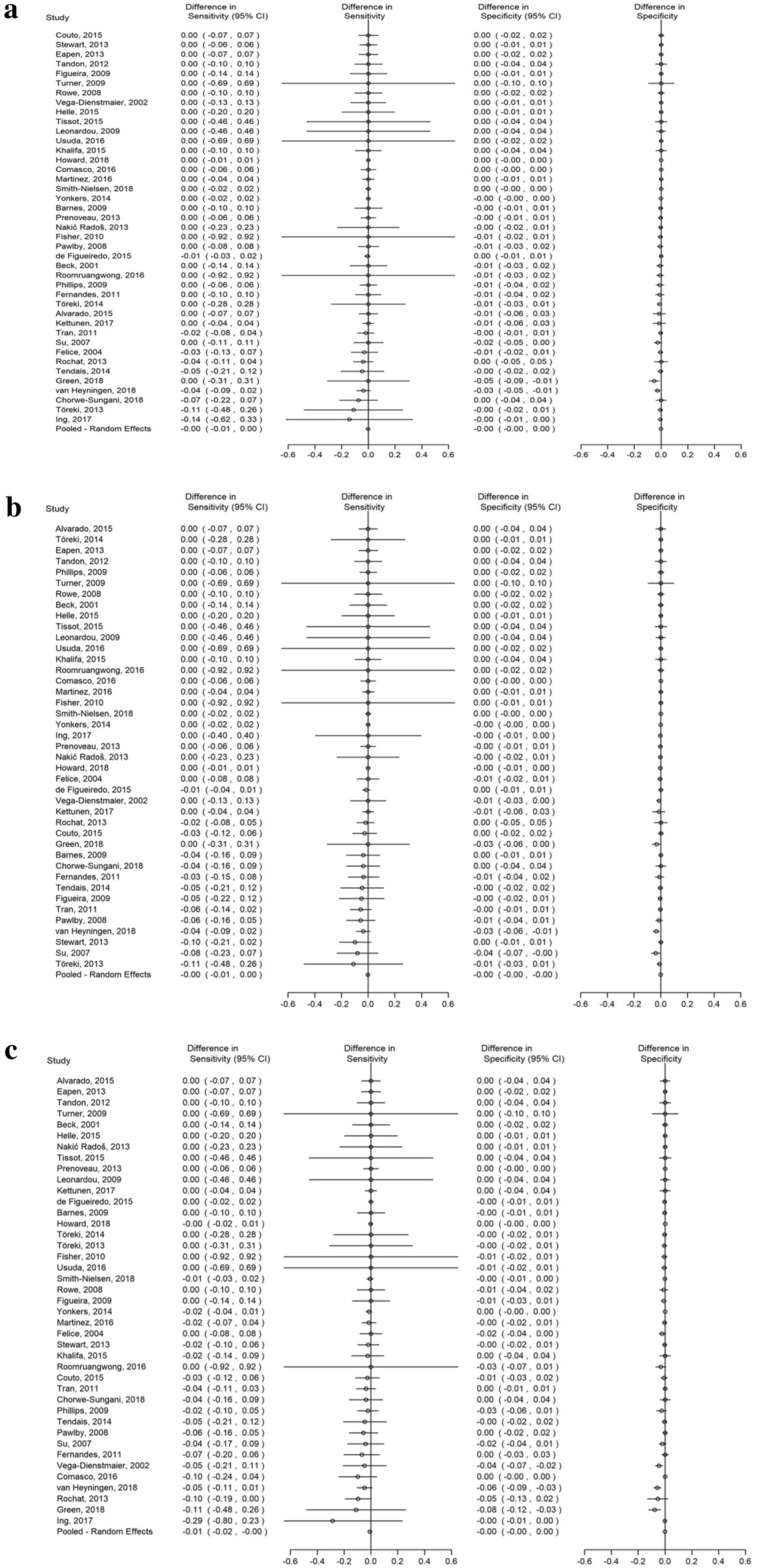
(**a**)–(**c**) Forest plots of the difference in sensitivity and specificity estimates between EPDS-9 and full EPDS among all studies at cut-offs (**a**) ≥ 10, (**b**) ≥ 11, and (**c**) ≥ 13.

Forest plots illustrating the difference in sensitivity and specificity estimates between the EPDS-9 and full EPDS for the most used cut-offs ≥ 10, ≥ 11, and ≥ 13 are shown in Fig. 2. At cut-offs of ≥ 10, ≥ 11, and ≥ 13, low heterogeneity existed in the differences across all 41 studies; τ^2^ was < 0.01 for both differences in sensitivity and specificity, and the widest 95% prediction intervals were − 0.01 to 0.01 for differences in sensitivity and − 0.00 to 0.00 for differences in specificity. Forest plots of the differences of sensitivity and specificity estimates for cut-offs ≥ 10, ≥ 11 and ≥ 13 between the EPDS-9 and full EPDS among studies by reference standard category are shown in Supplementary Figure S3.

## Discussion

The present study had two major findings. First, the scores between the continuous EPDS-9 and the full EPDS were highly correlated (0.998, 95% PI 0.991, 0.999). Second, across cut-offs, including the commonly used cut-offs of ≥ 10, ≥ 11, and ≥ 13, compared with the full EPDS, the EPDS-9 had similar sensitivity and specificity in screening major depression among pregnant and postpartum women, across all studies and for all three types of reference standard categories. In analyses pooled across reference standards, sensitivity was equivalent for cut-offs ≥ 7 to ≥ 12 and indeterminate for cut-offs ≥ 13 to ≥ 15. Specificity was equivalent for all cut-offs. Low heterogeneity in differences show that results were consistent across included studies.

Our findings about the EPDS-9 and full EPDS among pregnant and postpartum women are similar to results from a similar IPDMA on the equivalency of the screening accuracy of the PHQ-8 and PHQ-9, where the item removed in the shorter version also assessed self-harm. In that IPDMA, the screening accuracy between the PHQ-8 and PHQ-9 were similar across all cut-offs for detecting major depression^31^. Differences in sensitivity between the PHQ-8 and PHQ-9 were between 0.00 to 0.05, suggesting the sensitivity may be minimally reduced with the PHQ-8, although differences were deemed indeterminant. Specificity was equivalent for all cut-offs. We did not report positive predictive values in the present study, but these have been previously documented in our main IPDMA for the full EPDS^20^. For major depression prevalence values of 5–25%, positive predictive values for a cutoff of ≥ 11 compared to semi-structured interviews, for instance, ranged from 26 to 69%, and negative predictive values ranged from 93 to 99%. These would be similar for the EPDS-9.

Previous studies indicate that item 10 of the full EPDS overestimates the risk of suicidal ideation and identified substantially more people as at risk than scales designed to assess suicidal ideation risk^24,28^. Ideally, if researchers wish to assess suicide risk, a method designed specifically for that purpose, such as the P4 would be used given the limitations of EPDS item 10^21^. Potentially negative ramifications of using item 10 in research studies involve both resources and messaging to study participants. Ethically, all participants who score ≥ 1 on the item would need to be followed up with risk assessments, even though very few would be at risk, which could require substantial resources. Additionally, there is risk in impairing relationships with some women who must undergo these interviews even though they are not at risk. There are similar ramifications in clinical settings, where follow-up interviews would be needed for all women with positive EPDS screens and women with a non-zero item 10 score but negative screens overall.

The present study is the first meta-analysis using a large individual participant dataset to compare the measurement performance of the EPDS-9 and full EPDS, which is a major strength. The large sample size enabled us to generate precise estimates of correlations and equivalence for screening accuracy between the two versions of the EPDS. Furthermore, we compared results for the EPDS-9 and full EPDS from all studies and three different reference standard categories with all cut-offs, rather than just published cut-offs, which may result in bias due to selective cut-off reporting when individual participant data are not available^53^.

Limitations also need to be considered. First, we restricted meta-analysis to studies with complete data for full EPDS individual item scores (71%, 41 from 58 eligible studies) and were not able to include all studies. We do not know of any reason why studies that did and did not record item-level data might differ in the association between full EPDS and EPDS-9 scores. Secondly, although we categorized studies based on the interview administered, interviews might not have always been used as originally designed; for instance, it is possible that some interviewers may not have had the experience or training required to administer semi-structured interviews. The low heterogeneity in the main analysis, though, suggests that results are applicable across different diagnostic interviews. Third, we conducted a secondary analysis of data collected up to 2018 for a previously published IPDMA. Based on prediction intervals, which were all between − 0.01 to 0.01 for differences in sensitivity and − 0.00 to 0.00 for differences in specificity, however, additional data would not likely influence results meaningfully. It would not be a good use of resources to conduct additional studies on this research question. Fourth, we did not track inter-rater agreement for assessing eligibility at the title and abstract and full-text review levels.

In summary, this IPDMA showed that the EPDS-9 performs similarly to the full EPDS for assessing depressive symptom severity. The two EPDS versions also had similar screening accuracy in screening for major depression. The negative ramifications of false positive responses on item 10 suggest that using the EPDS-9 instead of the full EPDS should be considered as measurement performance is similar to the full EPDS.

## Supplementary Information

Supplementary Information.

## Data Availability

Data contribution agreements with primary study authors do not include permission to make their data publicly available, although the dataset used in this study will be archived through a McGill University repository (Borealis, https://borealisdata.ca/dataverse/depressdproject/). The R codes used for the analysis will be made publicly available through the same repository. Requests to access the dataset to verify study results but not for other purposes can be sent to the corresponding authors via the “Access Dataset” function on the repository website.
