# Supplementary material for: Individual participant data meta-analysis to compare EPDS accuracy to detect major depression with and without the self-harm item
Source: Sci Rep. 2023 Mar 10;13:4026. doi: 10.1038/s41598-023-29114-w (PMC10006408; doi:10.1038/s41598-023-29114-w)
Supplement: Supplementary file 1 — Supplementary Information. [file 41598_2023_29114_MOESM1_ESM.docx]

**Supplementary Material**

**Supplementary Methods 1.** Search strategies.

**Supplementary Methods 2.** QUADAS-2 ratings for each primary study included in the present study

**Supplementary Figure S1.** Flow diagram of study selection process.

**Supplementary Figure S2.** Forest plot of the Pearson correlation between EPDS-9 and full EPDS scores.

**Supplementary Figure S3.** Forest plots of the difference in sensitivity and specificity estimates at cut-off ≥ 10, ≥ 11, ≥ 13 between EPDS-9 and full EPDS among each reference standard (semi-structured diagnostic interviews, fully structured diagnostic interviews and MINI).

**Supplementary Table S1.** Reasons for exclusion for 257 articles excluded at full-text level.

**Supplementary Table S2.** Characteristics of included primary studies (N = 41).

**Supplementary Table S3.** Characteristics of eligible primary studies that did not provide data for the present study (N = 42).

**Supplementary Table S4.** Comparison of sensitivity and specificity estimates between EPDS-9 and full EPDS across cutoffs ≥ 7 to ≥ 15 for studies by reference standard type.

**Supplementary Material References**

**Supplementary Methods 1.** Search strategies.

**MEDLINE (OvidSP)**

1. EPDS.af.

2. Edinburgh Postnatal Depression.af.

3. Edinburgh Depression Scale.af.

4. or/1-3

5. Mass Screening/

6. Psychiatric Status Rating Scales/

7. "Predictive Value of Tests"/

8. "Reproducibility of Results"/

9. exp "Sensitivity and Specificity"/

10. Psychometrics/

11. Prevalence/

12. Reference Values/

13. Reference Standards/

14. exp Diagnostic Errors/

15. Mental Disorders/di, pc [Diagnosis, Prevention & Control]

16. Mood Disorders/di, pc [Diagnosis, Prevention & Control]

17. Depressive Disorder/di, pc [Diagnosis, Prevention & Control]

18. Depressive Disorder, Major/di, pc [Diagnosis, Prevention & Control]

19. Depression, Postpartum/di, pc [Diagnosis, Prevention & Control]

20. Depression/di, pc [Diagnosis, Prevention & Control]

21. validation studies.pt.

22. comparative study.pt.

23. screen*.af.

24. prevalence.af.

25. predictive value*.af.

26. detect*.ti.

27. sensitiv*.ti.

28. valid*.ti.

29. revalid*.ti.

30. predict*.ti.

31. accura*.ti.

32. psychometric*.ti.

33. identif*.ti.

34. specificit*.ab.

35. cut?off*.ab.

36. cut* score*.ab.

37. cut?point*.ab.

38. threshold score*.ab.

39. reference standard*.ab.

40. reference test*.ab.

41. index test*.ab.

42. gold standard.ab.

43. or/5-42

44. 4 and 43

**PsycINFO (OvidSP)**

1. EPDS.af.

2. Edinburgh Postnatal Depression.af.

3. Edinburgh Depression Scale.af.

4. or/1-3

5. Diagnosis/

6. Medical Diagnosis/

7. Psychodiagnosis/

8. Misdiagnosis/

9. Screening/

10. Health Screening/

11. Screening Tests/

12. Prediction/

13. Cutting Scores/

14. Psychometrics/

15. Test Validity/

16. screen*.af.

17. predictive value*.af.

18. detect*.ti.

19. sensitiv*.ti.

20. valid*.ti.

21. revalid*.ti.

22. accura*.ti.

23. psychometric*.ti.

24. specificit*.ab.

25. cut?off*.ab.

26. cut* score*.ab.

27. cut?point*.ab.

28. threshold score*.ab.

29. reference standard*.ab.

30. reference test*.ab.

31. index test*.ab.

32. gold standard.ab.

33. or/5-32

34. 4 and 33

**Web of Science (Web of Knowledge)**

#1. TS=(EPDS OR “Edinburgh Postnatal Depression” OR “Edinburgh Depression Scale”)

#2. TS=(screen* OR prevalence OR “predictive value*” OR detect* OR sensitiv* OR valid* OR revalid* OR predict* OR accura* OR psychometric* OR identif* OR specificit* OR cutoff* OR “cut off*” OR “cut* score*” OR cutpoint* OR “cut point*” OR “threshold score*” OR “reference standard*” OR “reference test*” OR “index test*” OR “gold standard” OR “reliab*”)

#2 AND #1

*Databases=SCI-EXPANDED, SSCI, A&HCI*

**Supplementary Methods 2.** QUADAS-2 ratings for each primary study included in the present study

| **First Author, Year** | **Domain 1: Participant Selection** | | | | | **Domain 2: Index Text** | | | | **Domain 3: Reference Standard** | | | | | **Domain 4: FLow and Timing** | | | | |
| --- | --- | --- | --- | --- | --- | --- | --- | --- | --- | --- | --- | --- | --- | --- | --- | --- | --- | --- | --- |
|  | **SQ1** | **SQ2** | **SQ3** | **RoB** | **AC** | **SQ1** | **SQ2** | **RoB** | **AC** | **SQ1** | **SQ2** | **SQ3** | **RoB** | **AC** | **SQ1** | **SQ2** | **SQ3** | **SQ4** | **RoB** |
| **Semi-Structured Interviews** | | | | | | | | | | | | | | | | | | | |
| **Barnes, 2009^1^** | U/C | Yes | Yes | U/C | U/C | N/A | N/A | Low | Low | Yes | U/C | U/C | U/C | Low | Yes | Yes | Yes | Yes | Low |
| **Beck, 2001^2^** | No | Yes | Yes | U/C | U/C | N/A | N/A | Low | Low | Yes | Yes | Yes | Low | Low | Yes | Yes | Yes | Yes | Low |
| **de Figueiredo, 2015^3^** | U/C | Yes | Yes | U/C | U/C | N/A | N/A | Low | Low | Yes | Yes | Yes | Low | Low | Yes | Yes | Yes | No | High |
| **Green, 2018^4^** | Yes | Yes | Yes | Low | U/C | N/A | N/A | Low | Low | Yes | Yes | Yes | Low | U/C | Yes | Yes | Yes | Yes | Low |
| **Helle, 2015^5^** | No | Yes | Yes | U/C | U/C | N/A | N/A | Low | Low | Yes | U/C | U/C | U/C | Low | U/C | Yes | Yes | U/C | U/C |
| **Howard, 2018^6^** | No | Yes | Yes | U/C | Low | N/A | N/A | Low | U/C | Yes | U/C | Yes | U/C | U/C | U/C | Yes | Yes | Yes | U/C |
| **Ing, 2017^7^** | U/C | Yes | Yes | Low | U/C | N/A | N/A | Low | Low | Yes | Yes | Yes | Low | Low | Yes | Yes | Yes | Yes | Low |
| **Kettunen, 2017^8^** | No | No | Yes | High | Low | N/A | N/A | Low | U/C | Yes | No | Yes | High | U/C | U/C | Yes | Yes | Yes | U/C |
| **Leonardou, 2009^9^** | Yes | Yes | Yes | Low | U/C | N/A | N/A | Low | Low | Yes | Yes | Yes | Low | Low | Yes | Yes | Yes | Yes | Low |
| **Nakić Radoš, 2013^10^** | U/C | Yes | Yes | U/C | Low | N/A | N/A | Low | Low | Yes | Yes | U/C | U/C | U/C | Yes | Yes | Yes | Yes | Low |
| **Pawlby, 2008^11^** | Yes | Yes | Yes | Low | U/C | N/A | N/A | Low | Low | Yes | U/C | Yes | U/C | Low | U/C | Yes | Yes | U/C | U/C |
| **Phillips, 2009^12^** | U/C | Yes | Yes | U/C | U/C | N/A | N/A | Low | Low | Yes | Yes | Yes | Low | Low | Yes | Yes | Yes | Yes | Low |
| **Prenoveau, 2013^13^** | U/C | Yes | Yes | U/C | Low | N/A | N/A | Low | Low | Yes | U/C | Yes | U/C | Low | IPD^a^ | Yes | Yes | U/C | U/C |
| **Rochat, 2013^14^** | U/C | Yes | Yes | Low | U/C | N/A | N/A | Low | U/C | Yes | U/C | U/C | U/C | Low | Yes | Yes | Yes | Yes | Low |
| **Smith-Nielsen, 2018^15^** | No | Yes | Yes | U/C | U/C | N/A | N/A | Low | Low | Yes | No | U/C | High | U/C | Yes | Yes | Yes | Yes | Low |
| **Stewart, 2013^16^** | No | Yes | Yes | U/C | U/C | N/A | N/A | Low | Low | Yes | Yes | Yes | Low | Low | Yes | Yes | Yes | No | U/C |
| **Tandon, 2012^17^** | No | Yes | U/C | High | Low | N/A | N/A | Low | Low | Yes | U/C | Yes | U/C | Low | Yes | Yes | Yes | Yes | Low |
| **Tendais, 2014^18^** | U/C | Yes | Yes | U/C | U/C | N/A | N/A | Low | Low | Yes | Yes | U/C | U/C | Low | U/C | U/C | Yes | U/C | U/C |
| **Tissot, 2015^19^** | No | Yes | Yes | U/C | Low | N/A | N/A | Low | Low | Yes | U/C | Yes | U/C | Low | U/C | Yes | Yes | U/C | U/C |
| **Töreki, 2013^20^** | U/C | Yes | Yes | U/C | Low | N/A | N/A | Low | Low | Yes | Yes | Yes | Low | U/C | Yes | Yes | Yes | Yes | Low |
| **Töreki, 2014^21^** | U/C | Yes | Yes | U/C | Low | N/A | N/A | Low | Low | Yes | Yes | Yes | Low | U/C | Yes | Yes | Yes | Yes | Low |
| **Tran, 2011^22^** | Yes | Yes | Yes | Low | U/C | N/A | N/A | Low | Low | Yes | Yes | Yes | Low | U/C | Yes | Yes | Yes | Yes | Low |
| **Turner, 2009^23^** | U/C | Yes | Yes | U/C | Low | N/A | N/A | Low | Low | Yes | U/C | Yes | U/C | U/C | U/C | Yes | Yes | Yes | U/C |
| **Vega-Dienstmaier, 2002^24^** | U/C | Yes | Yes | U/C | U/C | N/A | N/A | Low | U/C | Yes | U/C | U/C | U/C | U/C | Yes | Yes | Yes | Yes | Low |
| **Fully-Structured Interviews** | | | | | | | | | | | | | | | | | | | |
| **Felice, 2004^25^** | Yes | Yes | Yes | Low | Low | N/A | N/A | Low | Low | Yes | Yes | Yes | Low | Low | Yes | Yes | Yes | Yes | Low |
| **Fisher, 2010^26^** | U/C | Yes | Yes | Low | U/C | N/A | N/A | Low | Low | Yes | U/C | Yes | U/C | Low | Yes | Yes | Yes | Yes | Low |
| **Rowe, 2008^27^** | No | Yes | Yes | U/C | U/C | N/A | N/A | Low | Low | Yes | Yes | Yes | Low | Low | Yes | Yes | Yes | Yes | Low |
| **Yonkers, 2014^28^** | U/C | Yes | Yes | U/C | Low | N/A | N/A | Low | U/C | Yes | U/C | Yes | U/C | U/C | Yes | Yes | Yes | U/C | U/C |
| **Mini International Neuropsychiatric Interview (MINI)** | | | | | | | | | | | | | | | | | | | |
| **Alvarado, 2015^29^** | U/C | Yes | Yes | U/C | U/C | N/A | N/A | Low | Low | Yes | Yes | Yes | Low | U/C | Yes | Yes | Yes | Yes | Low |
| **Chorwe-Sungani, 2018^30^** | No | Yes | Yes | U/C | U/C | N/A | N/A | Low | Low | Yes | Yes | Yes | Low | Low | Yes | Yes | Yes | Yes | Low |
| **Couto, 2015^31^** | U/C | Yes | Yes | U/C | U/C | N/A | N/A | Low | Low | Yes | Yes | Yes | Low | Low | U/C | Yes | Yes | U/C | U/C |
| **Comasco, 2016^32^** | Yes | Yes | Yes | Low | Low | N/A | N/A | Low | U/C | Yes | U/C | Yes | U/C | U/C | Yes | Yes | Yes | Yes | Low |
| **Eapen, 2013^33^** | No | Yes | Yes | U/C | U/C | N/A | N/A | Low | Low | Yes | U/C | Yes | U/C | Low | U/C | Yes | Yes | No | U/C |
| **Fernandes, 2011^34^** | Yes | Yes | Yes | Low | Low | N/A | N/A | Low | Low | Yes | U/C | Yes | U/C | Low | Yes | Yes | Yes | Yes | Low |
| **Figueira, 2009^35^** | U/C | Yes | Yes | U/C | Low | N/A | N/A | Low | Low | Yes | Yes | Yes | Low | Low | Yes | Yes | Yes | Yes | Low |
| **Khalifa, 2015^36^** | No | Yes | Yes | U/C | U/C | N/A | N/A | Low | Low | Yes | Yes | Yes | Low | Low | Yes | No | Yes | No | High |
| **Martinez, 2016^37^** | U/C | Yes | Yes | U/C | U/C | N/A | N/A | Low | Low | Yes | U/C | Yes | U/C | U/C | Yes | Yes | Yes | Yes | Low |
| **Roomruangwong, 2016^38^** | U/C | Yes | Yes | U/C | U/C | N/A | N/A | Low | Low | Yes | U/C | Yes | U/C | U/C | Yes | Yes | Yes | Yes | Low |
| **Su, 2007^39^** | U/C | Yes | Yes | U/C | U/C | N/A | N/A | Low | Low | Yes | Yes | Yes | Low | Low | Yes | Yes | Yes | Yes | Low |
| **Usuda, 2016^40^** | U/C | Yes | Yes | Low | U/C | N/A | N/A | Low | Low | Yes | U/C | Yes | U/C | U/C | Yes | Yes | Yes | Yes | Low |
| **van Heyningen, 2018^41^** | U/C | Yes | Yes | Low | U/C | N/A | N/A | Low | Low | Yes | U/C | Yes | U/C | Low | Yes | Yes | Yes | Yes | Low |

**Abbreviations**: AC: acceptability concern, RoB: risk of bias, SQ: signalling question, N/A: not applicable; U/C: Unclear

^a^Rating varies at the individual participant level

**Supplementary Figure S1.** Flow diagram of study selection process.

**56** Eligibe EPDS studies contributed primary data (N = 15,020)

**25** Eligible EPDS studies did not provide primary data

24 Author did not respond or unable to contribute data

1 Decision to contribute still pending

(N = 6,569)

**2** Studies the search did not retrieve, and were provided by authors of other published eligible studies (N = 512)

**17** Studies contributed primary data but unable to provide item-level data for each EPDS item (N = 4,626)

**58** EPDS datasets with primary data (N = 15,532)

**41** EPDS studies included in the present study (N = 10,906)

**4434** Unique titles or abstracts identified and screened for potential eligibility

**121** Articles meeting eligibility criteria

**40** Articles excluded owing to duplicate participant sample

**81** Unique studies meeting eligibility criteria (N = 21,589)

**378** Full text articles reviewed for eligibility

**4056** Titles or abstracts excluded

**257** Articles excluded

8 No original data

8 No EPDS

48 No major depression

49 No validated interview to assess major depression

21 >2 weeks between EPDS and diagnostic interview

90 Sample selected for known distress, mental health diagnosis, or psychiatric setting

9 No pregnant or postpartum women

6 No adults

1 No major depression cases

17 Could not determine eligibility

**Supplementary Figure S2.** Forest plot of the Pearson correlation between EPDS-9 and full EPDS scores.

**
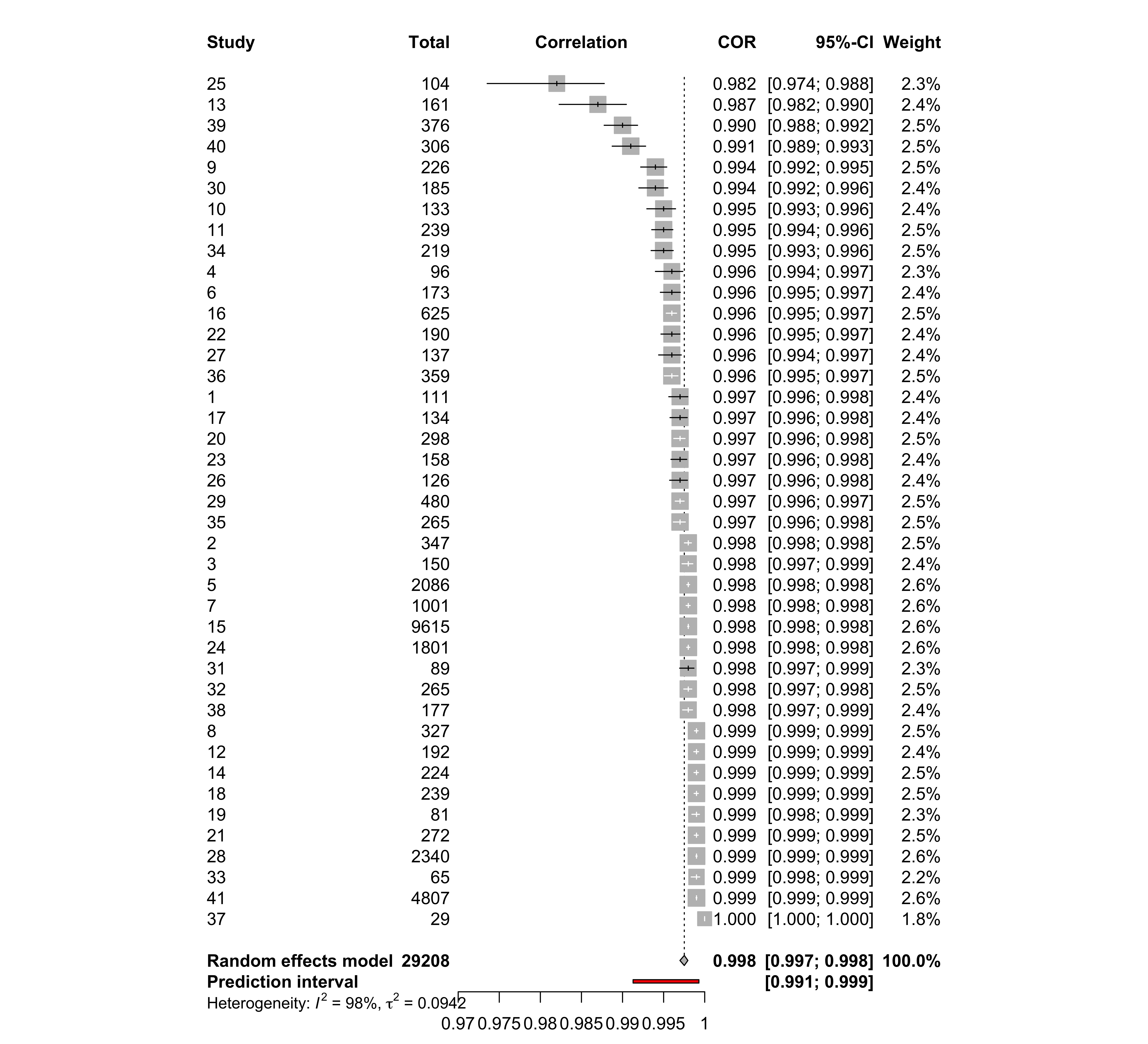
**

**Supplementary Figure S3a.** Forest plots of the difference in sensitivity and specificity estimates at cut-off ≥ 10 between EPDS-9 and full EPDS among semi-structured diagnostic interviews.


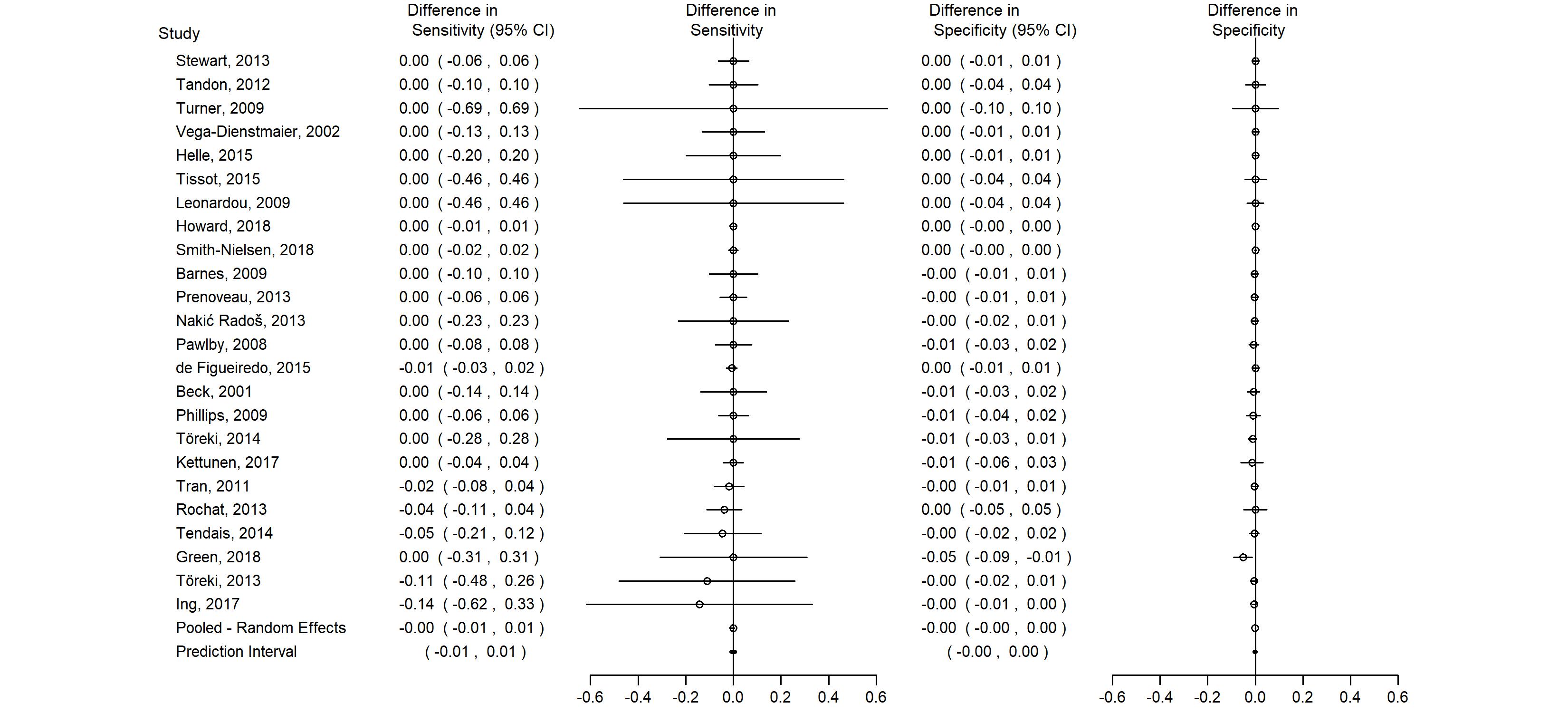


**Supplementary Figure S3b.** Forest plots of the difference in sensitivity and specificity estimates at cut-off ≥ 11 between EPDS-9 and full EPDS among semi-structured diagnostic interviews.

**
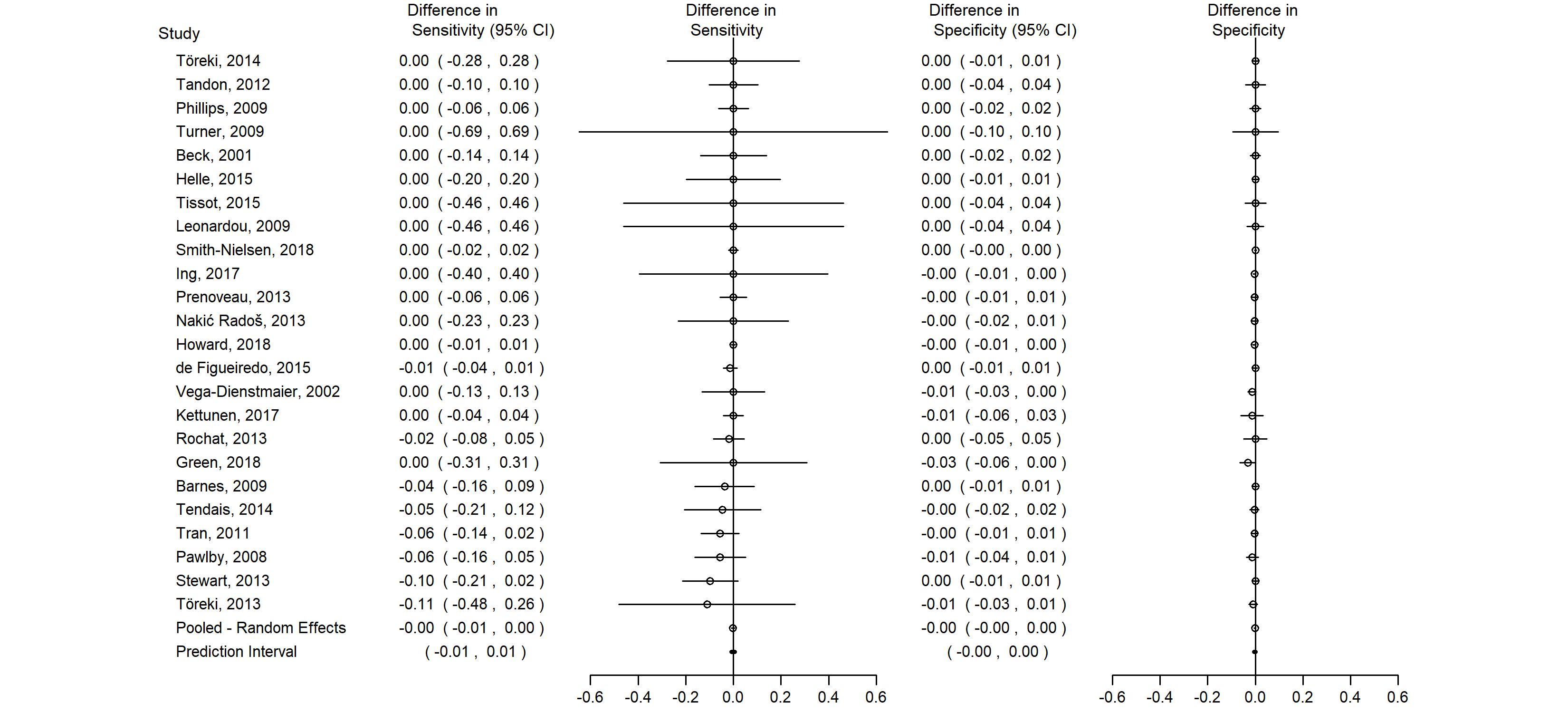
**

**Supplementary Figure S3c.** Forest plots of the difference in sensitivity and specificity estimates at cut-off ≥ 13 between EPDS-9 and full EPDS among semi-structured diagnostic interviews.

**
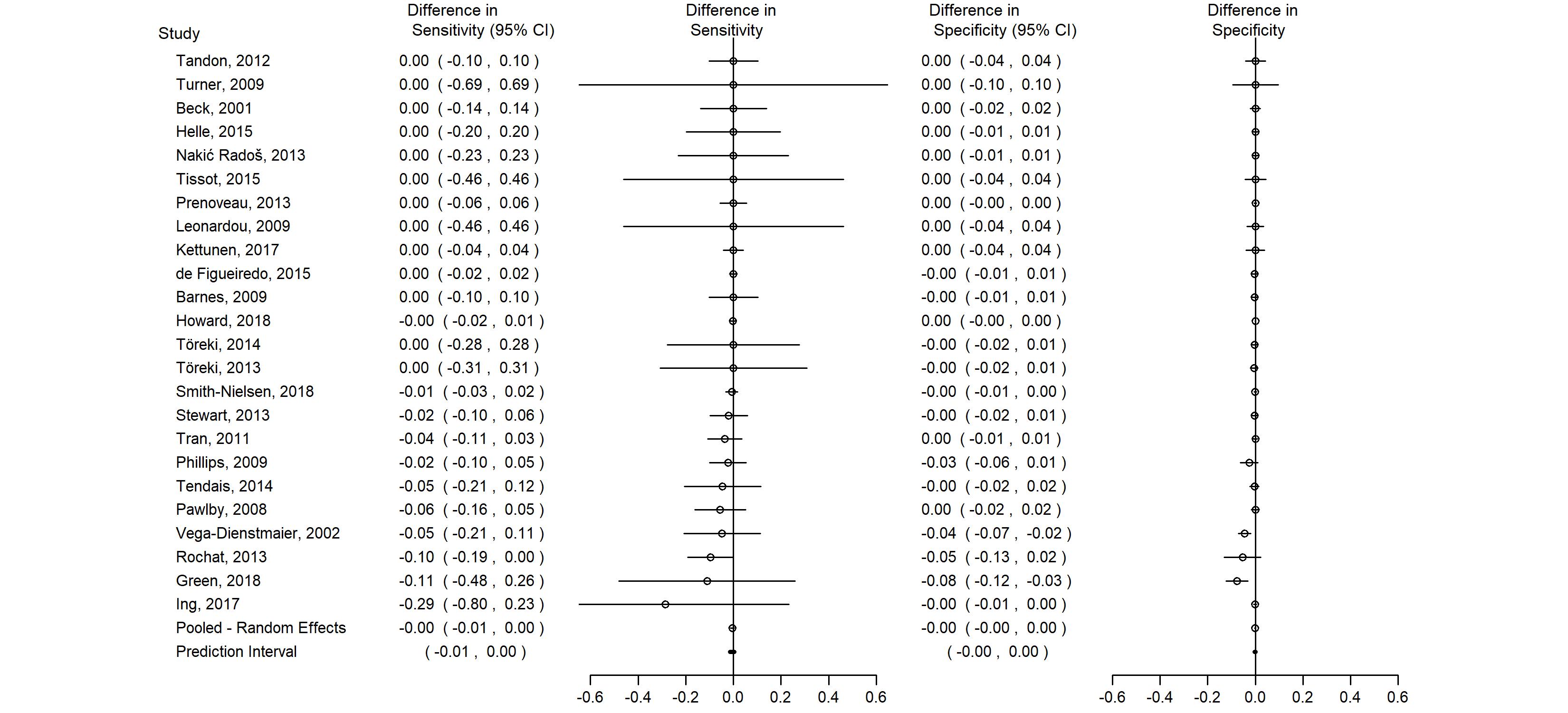
**

**Supplementary Figure S3d.** Forest plots of the difference in sensitivity and specificity estimates at cut-off ≥ 10 between EPDS-9 and full EPDS among fully structured diagnostic interviews (MINI excluded).

**
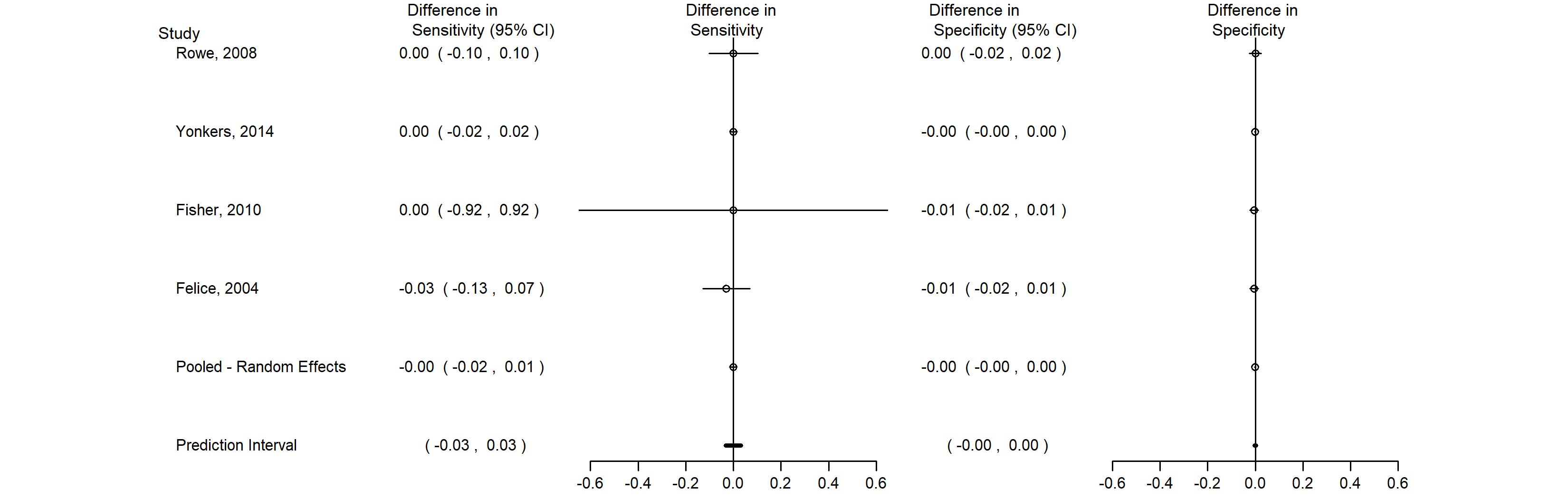
**

**Supplementary Figure S3e.** Forest plots of the difference in sensitivity and specificity estimates at cut-off ≥ 11 between EPDS-9 and full EPDS among fully structured diagnostic interviews (MINI excluded).

**
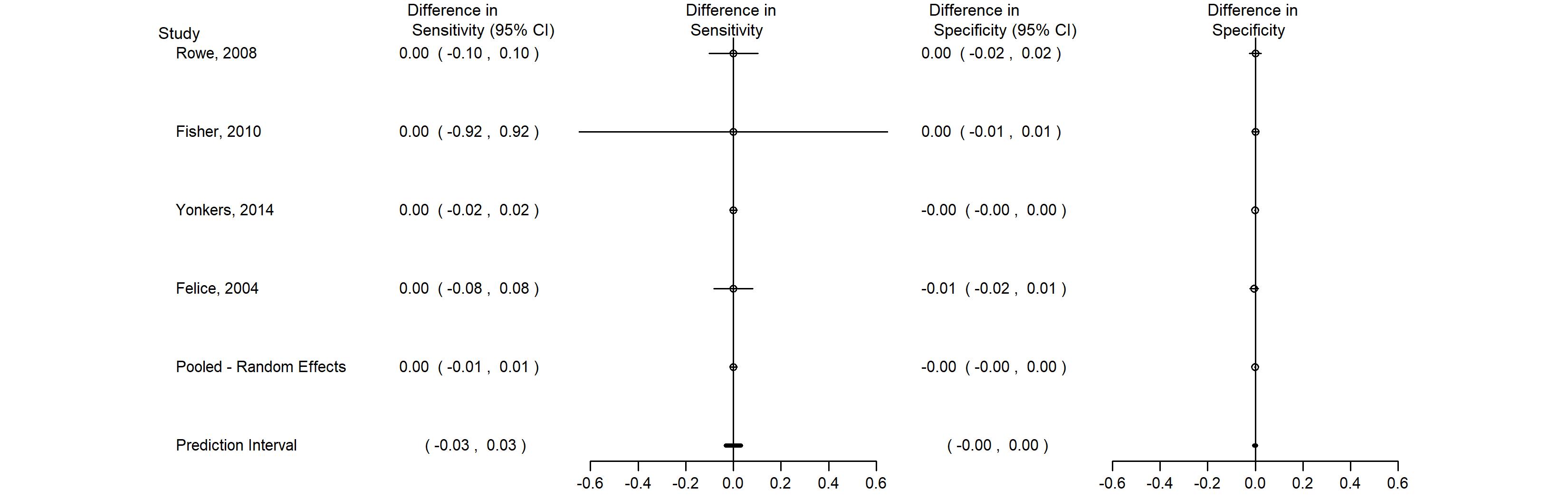
**

**Supplementary Figure S3f.** Forest plots of the difference in sensitivity and specificity estimates at cut-off ≥ 13 between EPDS-9 and full EPDS among fully structured diagnostic interviews (MINI excluded).

**
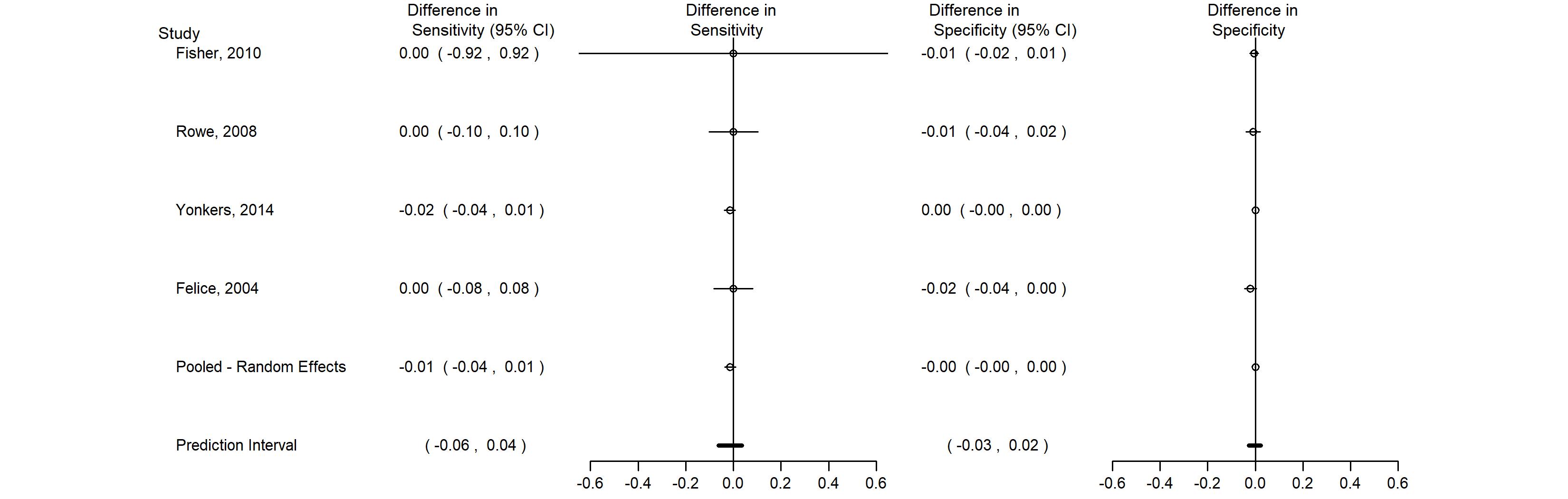
**

**Supplementary Figure S3g.** Forest plots of the difference in sensitivity and specificity estimates at cut-off ≥ 10 between EPDS-9 and full EPDS among MINI.


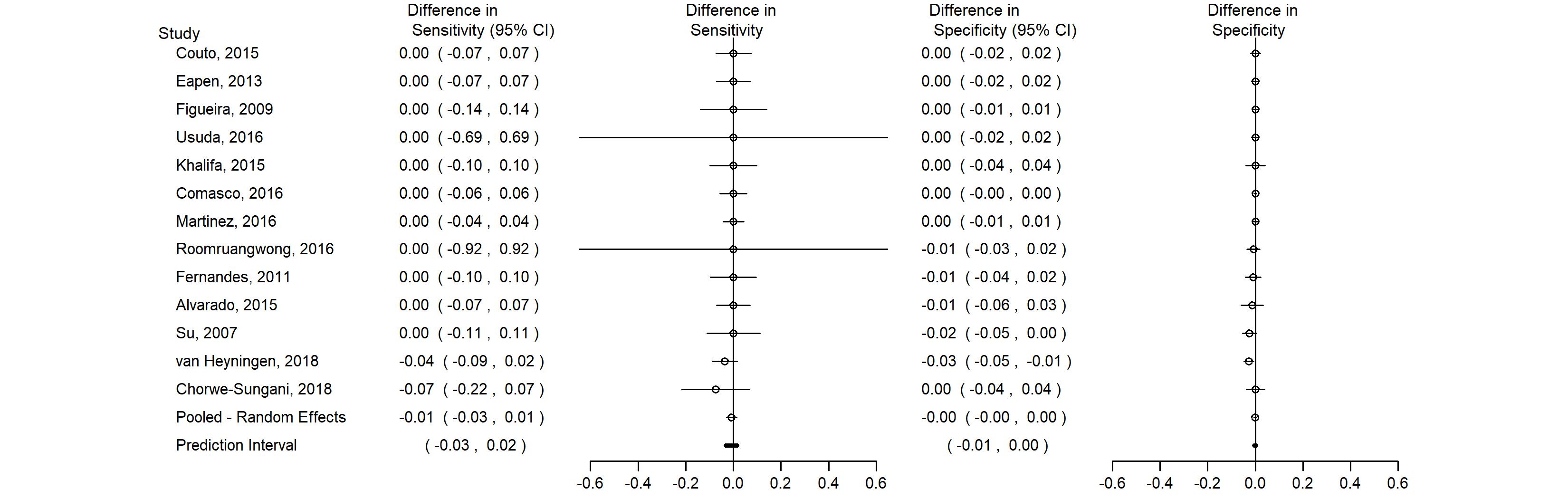


**Supplementary Figure S3h.** Forest plots of the difference in sensitivity and specificity estimates at cut-off ≥ 11 between EPDS-9 and full EPDS among MINI.

**
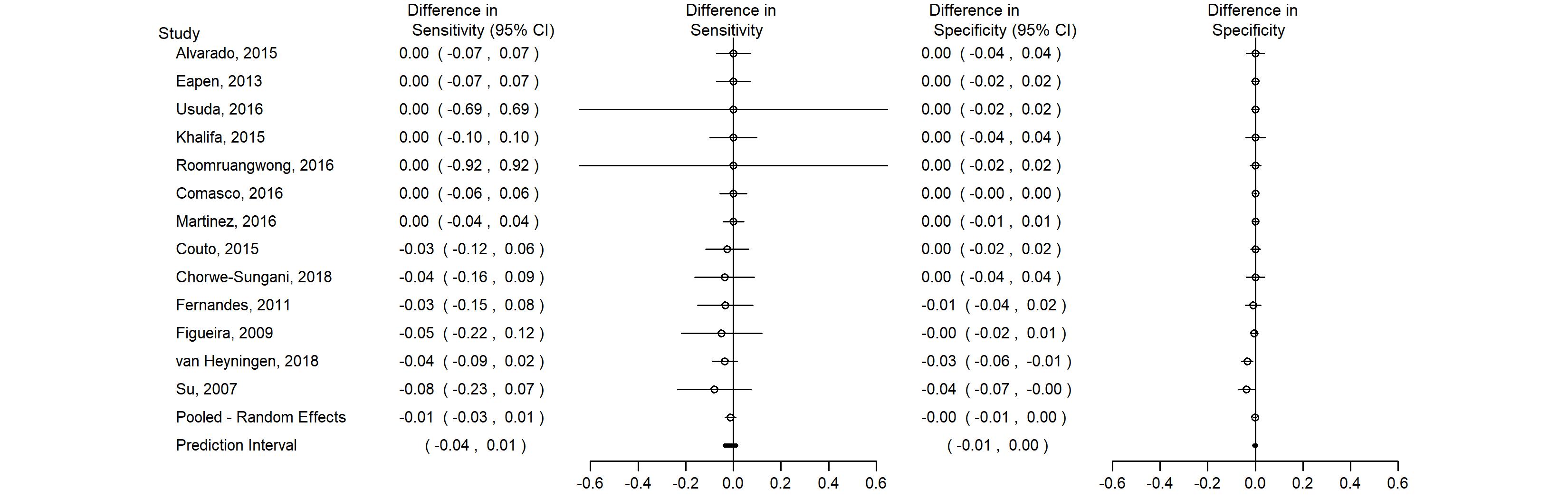
**

**Supplementary Figure S3i.** Forest plots of the difference in sensitivity and specificity estimates at cut-off ≥ 13 between EPDS-9 and full EPDS among MINI.


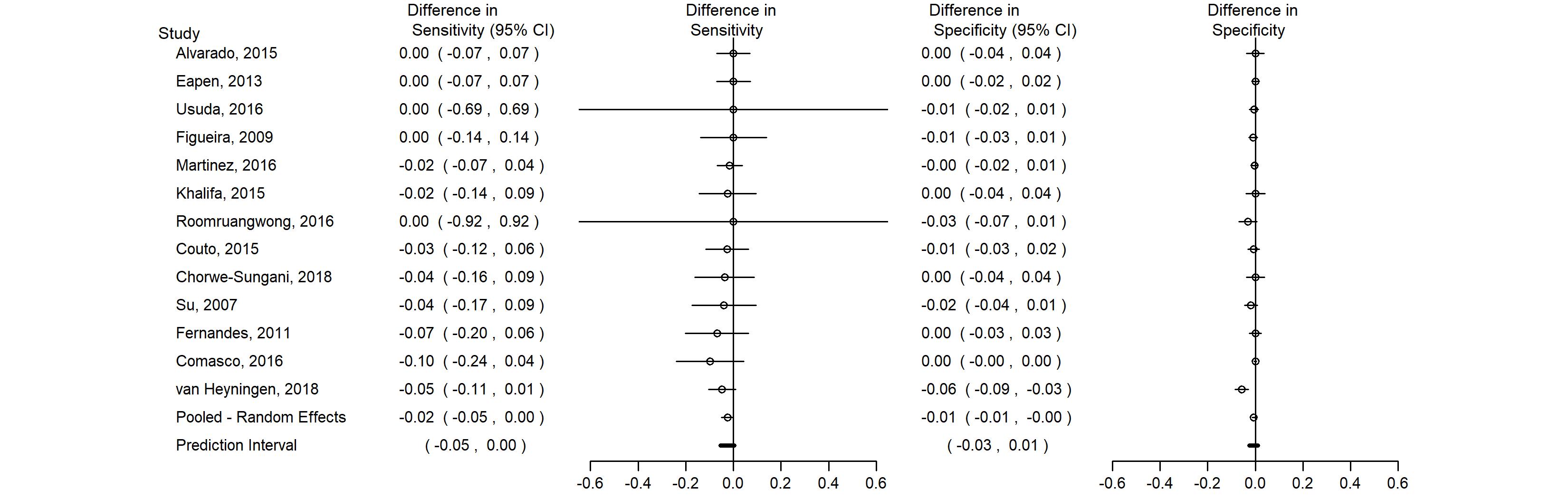


**Supplementary Table S1.** Reasons for exclusion for 257 articles excluded at full-text level.

| **Reference** | **Reason For Exclusion** |
| --- | --- |
| Abiodun OA. Postnatal depression in primary care populations in Nigeria. *General Hospital Psychiatry*. 2006;28:133. | Could not determine eligibility^a^ |
| Abou-Saleh MT, Ghubash R, Karim L, Krymski M, Bhai I. Hormonal aspects of postpartum depression. *Psychoneuroendocrinology*. 1998;23:465. | > 2 weeks between EPDS and diagnostic interview |
| Aceti F, Baglioni V, Ciolli P, De Bei F, Di Lorenzo F, Ferracuti S, Giacchetti N, Marini I, Meuti V, Motta P, Roma P, Zaccagni M, Williams R. Maternal attachment patterns and personality in postpartum depression. *Rivista di Psichiatria*. 2012;47:214. | Sample selected for known distress, mental health diagnosis, or psychiatric setting |
| Adewuya AO, Eegunranti AB, Lawal AM. Prevalence of postnatal depression in Western Nigerian women: a controlled study. *International Journal of Psychiatry in Clinical Practice*. 2005;9:60. | Could not determine eligibility^a^ |
| Adewuya AO. Early postpartum mood as a risk factor for postnatal depression in Nigerian women. *American Journal of Psychiatry*. 2006;163:1435. | No validated interview to assess major depression |
| Ahn S, Corwin EJ. The association between breastfeeding, the stress response, inflammation, and postpartum depression during the postpartum period: Prospective cohort study. *International Journal of Nursing Studies*. 2015;52:1582. | No major depression |
| Al-Modayfer O, Alatiq Y, Khair O, Abdelkawi S. Postpartum depression and related risk factors among Saudi females. *International Journal of Culture and Mental Health*. 2015;8:316. | No validated interview to assess major depression |
| Alami KM, Kadri N, Berrada S. Prevalence and psychosocial correlates of depressed mood during pregnancy and after childbirth in a Moroccan sample. *Archives of Women's Mental Health*. 2006;9:343. | Could not determine eligibility^a^ |
| Albacar G, Sans T, MartinSantos R, GarciaEsteve L, Guillamat R, Sanjuan J, Canellas F, Carot JM, Gratacos M, Bosch J, Gaviria A, Labad A, Zotes AG, Vilella E. Thyroid function 48 h after delivery as a marker for subsequent postpartum depression. *Psychoneuroendocrinology*. 2010;35:738. | Sample selected for known distress, mental health diagnosis, or psychiatric setting |
| Albacar G, Sans T, MartinSantos R, GarciaEsteve L, Guillamat R, Sanjuan J, Canellas F, Gratacos M, Cavalle P, Arija V, Gaviria A, GutierrezZotes A, Vilella E. An association between plasma ferritin concentrations measured 48h after delivery and postpartum depression. *Journal of Affective Disorders*. 2011;131:136. | Sample selected for known distress, mental health diagnosis, or psychiatric setting |
| Alexander S, Palmer C, Stone PC. Evaluation of screening instruments for depression and anxiety in breast cancer survivors. *Breast Cancer Research & Treatment*. 2010;122:573. | No pregnant or postpartum women |
| Algul A, Semiz UB, Dundar O, Ates MA, Basoglu C, Ebrinc S, Doruk A, Gecici O, Cetin M. Psychosocial and Hormone Related Risk Factors for Early Postnatal Depressive Symptoms in Turkish Women. *Neurology Psychiatry and Brain Research*. 2008;15:117. | No major depression |
| Alvarado-Esquivel C, Sifuentes-Alvarez A, Estrada-Martinez S, Salas-Martinez C, Hernandez-Alvarado AB, Ortiz-Rocha SG, Garcia-Lopez CR, Torres-Castorena A, Sandoval-Herrera F. Prevalence of postnatal depression in women attending public hospitals in Durango, Mexico. *Gaceta Medica de Mexico*. 2010;146:1. | No validated interview to assess major depression |
| Alvarado-Esquivel C, Sifuentes-Alvarez A, Salas-Martinez C. Unhappiness with the Fetal Gender is associated with Depression in Adult Pregnant Women Attending Prenatal Care in a Public Hospital in Durango, Mexico. *International Journal of Biomedical Science*. 2016;12:36. | Sample selected for known distress, mental health diagnosis, or psychiatric setting |
| Areias ME, Kumar R, Barros H, Figueiredo E. Comparative incidence of depression in women and men, during pregnancy and after childbirth. Validation of the Edinburgh Postnatal Depression Scale in Portuguese mothers. *The* *British Journal of Psychiatry*. 1996;169:30. | No validated interview to assess major depression |
| Areias ME, Kumar R, Barros H, Figueiredo E. Correlates of postnatal depression in mothers and fathers. *The* *British Journal of Psychiatry*. 1996;169:36. | No validated interview to assess major depression |
| Austin MP, Dudley M, Launders C, Dixon C, MacartneyBourne F. Description and evaluation of a domiciliary perinatal mental health service focussing on early intervention. *Archives of Women's Mental Health*. 1999;2:169. | Sample selected for known distress, mental health diagnosis, or psychiatric setting |
| Austin MP, Frilingos M, Lumley J, Hadzi-Pavlovic D, Roncolato W, Acland S, Saint K, Segal N, Parker G. Brief antenatal cognitive behaviour therapy group intervention for the prevention of postnatal depression and anxiety: a randomised controlled trial. *Journal of Affective Disorders*. 2008;105:35. | Sample selected for known distress, mental health diagnosis, or psychiatric setting |
| Austin MP, Hadzi-Pavlovic D, Priest SR, Reilly N, Wilhelm K, Saint K, Parker G. Depressive and anxiety disorders in the postpartum period: how prevalent are they and can we improve their detection? *Archives of Women's Mental Health*. 2010;13:395. | No major depression |
| Austin MP, Hadzi-Pavlovic D, Saint K,Parker G. Antenatal screening for the prediction of postnatal depression: validation of a psychosocial Pregnancy Risk Questionnaire. *Acta Psychiatrica Scandinavica*. 2005;112:310. | No major depression |
| Azar R, Paquette D, Zoccolillo M, Baltzer F, Tremblay RE. The association of major depression, conduct disorder, and maternal overcontrol with a failure to show a cortisol buffered response in 4-month-old infants of teenage mothers. *Biological Psychiatry*. 2007;62:573. | No adults |
| Bagedahl-Strindlund M, Borjesson KM. Postnatal depression: a hidden illness. *Acta Psychiatrica Scandinavica*. 1998;98:272. | Sample selected for known distress, mental health diagnosis, or psychiatric setting |
| Bawahab JA, Alahmadi JR, Ibrahim AM. Prevalence and determinants of antenatal depression among women attending primary health care centers in Western Saudi Arabia. *Saudi Medical Journal*. 2017;38:1237. | No major depression |
| Bergant AM, Heim K, Ulmer H, Illmensee K. Early postnatal depressive mood: associations with obstetric and psychosocial factors. *Journal of Psychosomatic Research*. 1999;46:391. | No major depression |
| Bergant AM, Nguyen T, Heim K, Ulmer H, Dapunt O. German language version and validation of the Edinburgh postnatal depression scale. *Deutsche Medizinische Wochenschrift*. 1998;123:35. | No validated interview to assess major depression |
| Bhusal BR, Bhandari N, Chapagai M, Gavidia T. Validating the Edinburgh Postnatal Depression Scale as a screening tool for postpartum depression in Kathmandu, Nepal. *International Journal of Mental Health Systems*. 2016;10:71. | No validated interview to assess major depression |
| Bick DE, MacArthur C, Lancashire RJ. What influences the uptake and early cessation of breast feeding? *Midwifery*. 1998;14:242. | No major depression |
| Bloch M, Rotenberg N, Koren D, Klein E. Risk factors associated with the development of postpartum mood disorders. *Journal of Affective Disorders*. 2005;88:9. | > 2 weeks between EPDS and diagnostic interview |
| Boath E, Cox J, Lewis M, Jones P, Pryce A. When the cradle falls: the treatment of postnatal depression in a psychiatric day hospital compared with routine primary care. *Journal of Affective Disorders*. 1999;53:143. | Sample selected for known distress, mental health diagnosis, or psychiatric setting |
| Bodenlos KL, Maranda L, Deligiannidis KM. Comparison of the use of the EPDS-3 vs. EPDS-10 to identify women at risk for peripartum depression. *Obstetrics & Gynecology*. 2016;127:89S-90S. | No major depression |
| Boyce P, Hickey A. Psychosocial risk factors to major depression after childbirth. *Social Psychiatry & Psychiatric Epidemiology*. 2005;40:605. | Sample selected for known distress, mental health diagnosis, or psychiatric setting |
| Boyce P, Stubbs J, Todd A. The Edinburgh Postnatal Depression Scale: validation for an Australian sample. *Australian & New Zealand Journal of Psychiatry*. 1993;27:472. | Sample selected for known distress, mental health diagnosis, or psychiatric setting |
| Bränn E, Papadopoulos F, Fransson E, White R, Edvinsson Å, Hellgren C, Kamali-Moghaddam M, Boström A, Schiöth HB, Sundström-Poromaa I, Skalkidou A. Inflammatory markers in late pregnancy in association with postpartum depression—A nested case-control study. *Psychoneuroendocrinology*. 2017;79:146-59. | Sample selected for known distress, mental health diagnosis, or psychiatric setting |
| Browne JC, Scott KM, Silvers KM. Fish consumption in pregnancy and omega-3 status after birth are not associated with postnatal depression. *Journal of Affective Disorders*. 2006;90:131. | Sample selected for known distress, mental health diagnosis, or psychiatric setting |
| Brugha TS, Wheatley S, Taub NA, Culverwell A, Friedman T, Kirwan P, Jones DR, Shapiro DA. Pragmatic randomized trial of antenatal intervention to prevent post-natal depression by reducing psychosocial risk factors. *Psychological Medicine*. 2000;30:1273. | Sample selected for known distress, mental health diagnosis, or psychiatric setting |
| Bunevicius A, Kusminskas L, Bunevicius R. Validation of the Lithuanian version of the Edinburgh Postnatal Depression Scale. *Medicina*. 2009;45:544. | No validated interview to assess major depression |
| Bunevicius A, Kusminskas L, Bunevicius R. Validity of the Edinburgh Postnatal Depression Scale. *European Psychiatry*. 2009;24. | No validated interview to assess major depression |
| Burns A, O'Mahen H, Baxter H, Bennert K, Wiles N, Ramchandani P, Turner K, Sharp D, Thorn J, Noble S, Evans J. A pilot randomised controlled trial of cognitive behavioural therapy for antenatal depression. *BMC Psychiatry*. 2013;13:Art 33. | Sample selected for known distress, mental health diagnosis, or psychiatric setting |
| Byatt N, Biebel K, Simas TAM, Sarvet B, Ravech M, Allison J, Straus J. Improving perinatal depression care: The Massachusetts Child Psychiatry Access Project for Moms. *General Hospital Psychiatry*. 2016;40:12. | No major depression |
| Byatt N, Cox L, Simas TA, Biebel K, Sankaran P, Swartz HA, Weinreb L. Access to pharmacotherapy amongst women with bipolar disorder during pregnancy: a preliminary study. *Psychiatric Quarterly*. 2018;89:183-90. | Sample selected for known distress, mental health diagnosis, or psychiatric setting |
| Byatt N, Moore Simas TA, Biebel K, Sankaran P, Pbert L, Weinreb L, Ziedonis D, Allison J. PRogram In Support of Moms (PRISM): a pilot group randomized controlled trial of two approaches to improving depression among perinatal women. *Journal of Psychosomatic Obstetrics & Gynecology*. 2018;39:297-306. | Sample selected for known distress, mental health diagnosis, or psychiatric setting |
| Caramlau I, Barlow J, Sembi S, McKenzie-McHarg K, McCabe C. Mums 4 Mums: structured telephone peer-support for women experiencing postnatal depression. Pilot and exploratory RCT of its clinical and cost effectiveness. *Trials*. 2011;12:88. | No original data |
| Carothers AD, Murray L. Estimating psychiatric morbidity by logistic regression: application to post-natal depression in a community sample. *Psychological Medicine*. 1990;20:695. | No validated interview to assess major depression |
| Carpiniello B, Pariante CM, Serri F, Costa G, Carta MG. Validation of the Edinburgh Postnatal Depression Scale in Italy. *Journal of Psychosomatic Obstetrics & Gynecology*. 1997;18:280. | No validated interview to assess major depression |
| Castanon SC, Pinto LJ. Use of the Edinburgh Postnatal Depression Scale to detect postpartum depression *Revista Medica de Chile*. 2008;136:851. | Sample selected for known distress, mental health diagnosis, or psychiatric setting |
| Chaudron LH, Nirodi N. The obsessive-compulsive spectrum in the perinatal period: a prospective pilot study. *Archives of Women's Mental Health*. 2010;13:403. | > 2 weeks between EPDS and diagnostic interview |
| Chee CY, Chong YS, Ng TP, Lee DT, Tan LK, Fones CS. The association between maternal depression and frequent non-routine visits to the infant's doctor--a cohort study. *Journal of Affective Disorders*. 2008;107:247. | Sample selected for known distress, mental health diagnosis, or psychiatric setting |
| Chee CYI, Lee DTS, Chong YS, Tan LK, Ng TR, Fones CSL. Confinement and other psychosocial factors in perinatal depression: A transcultural study in Singapore. *Journal of Affective Disorders*. 2005;89:157. | Sample selected for known distress, mental health diagnosis, or psychiatric setting |
| Chen H, Bautista D, Ch'ng YC, Li W, Chan E, Rush AJ. Screening for postnatal depression in Chinese-speaking women using the Hong Kong translated version of the Edinburgh Postnatal Depression Scale. *Asia-Pacific psychiatry: Official Journal of the Pacific Rim College of Psychiatrists*. 2013;5:E64. | No validated interview to assess major depression |
| Chibanda D, Verhey R, Gibson LJ, Munetsi E, Machando D, Rusakaniko S, Munjoma R, Araya R, Weiss HA, Abas M. Validation of screening tools for depression and anxiety disorders in a primary care population with high HIV prevalence in Zimbabwe. *Journal of Affective Disorders*. 2016;198:50. | No EPDS |
| Clarke PJ. Validation of two postpartum depression screening scales with a sample of First Nations and Metis women. *Canadian Journal of Nursing Research*. 2008;40:113. | No major depression |
| Class QA, Verhulst J, Heiman JR. Exploring the heterogeneity in clinical presentation and functional impairment of postpartum depression. *Journal of Reproductive and Infant Psychology*. 2013;31:183. | Sample selected for known distress, mental health diagnosis, or psychiatric setting |
| Clifford C, Day A, Cox J, Werrett J. A cross-cultural analysis of the use of the Edinburgh Post-Natal Depression Scale (EPDS) in health visiting practice. *Journal of Advanced Nursing*. 1999;30:655. | No validated interview to assess major depression |
| Coleman R, Morison L, Paine K, Powell RA, Walraven G. Women's reproductive health and depression: a community survey in the Gambia, West Africa. *Social Psychiatry & Psychiatric Epidemiology*. 2006;41:720. | No validated interview to assess major depression |
| Cooper PJ, Murray L, Wilson A, Romaniuk H. Controlled trial of the short- and long-term effect of psychological treatment of post-partum depression. I. Impact on maternal mood. *The* *British Journal of Psychiatry*. 2003;182:412. | Sample selected for known distress, mental health diagnosis, or psychiatric setting |
| Costas J, Gratacos M, Escaramis G, Martin-Santos R, de Diego Y, Baca-Garcia E, Canellas F, Estivill X, Guillamat R, Guitart M,Gutierrez-Zotes A, Garcia-Esteve L, Mayoral F, Molto MD, Phillips C, Roca M, Carracedo A, Vilella E, Sanjuan J. Association study of 44 candidate genes with depressive and anxiety symptoms in post-partum women. *Journal of Psychiatric Research*. 2010;44:717. | Sample selected for known distress, mental health diagnosis, or psychiatric setting |
| Cox J. Use and misuse of the Edinburgh Postnatal Depression Scale (EPDS): a ten point 'survival analysis'. *Archives of Women's Mental Health*. 2017;20:789. | No original data |
| Cox JL, Chapman G, Murray D, Jones P. Validation of the Edinburgh Postnatal Depression Scale (EPDS) in non-postnatal women. *Journal of Affective Disorders*. 1996;39:185. | No validated interview to assess major depression |
| Cox JL, Holden JM, Sagovsky R. Detection of postnatal depression. Development of the 10-item Edinburgh Postnatal Depression Scale. *The* *British Journal of Psychiatry*. 1987;150:782. | Sample selected for known distress, mental health diagnosis, or psychiatric setting |
| Cox JL, Murray D, Chapman G. A controlled study of the onset, duration and prevalence of postnatal depression. *The* *British Journal of Psychiatry*. 1993;163:27. | No validated interview to assess major depression |
| de Souza Ribeiro Martins C, dos Santos Motta JV, Quevedo LA, de Matos MB, Pinheiro KAT, de Mattos Souza LD, da Silva RA, Pinheiro RT, da Cunha Coelho FM. Comparison of two instruments to track depression symptoms during pregnancy in a sample of pregnant teenagers in Southern Brazil. *Journal of Affective Disorders*. 2015;177:95. | No adults |
| Dennis CL, Hodnett E, Kenton L, Weston J, Zupancic J, Stewart DE, Kiss A. Effect of peer support on prevention of postnatal depression among high risk women: multisite randomised controlled trial. *BMJ*. 2009;338:a3064. | Sample selected for known distress, mental health diagnosis, or psychiatric setting |
| di Giacomo E, Colmegna F, Pescatore F, Aspesi F, Fotiadou M, Clerici M. The burden of personality disorders on the DSM 5 addiction to tobacco during pregnancy. *Comprehensive Psychiatry*. 2018;84:101-5. | Sample selected for known distress, mental health diagnosis, or psychiatric setting |
| Drozd F, Andersen CE, Haga SM, Slinning K, Bjørkli CA. User experiences and perceptions of internet interventions for depression. InInnovations in Global Maternal Health: Improving Prenatal and Postnatal Care Practices 2020. *IGI Global .* 2018;27:369-94. | No original data |
| e Couto TC, Brancaglion MY, Cardoso MN, Faria GC, Garcia FD, Nicolato R, Aguiar RA, Leite HV, Corrêa H. Suicidality among pregnant women in Brazil: prevalence and risk factors. *Archives of Women's Mental Health*. 2016;19:343-8. | Sample selected for known distress, mental health diagnosis, or psychiatric setting |
| Ebeigbe PN, Akhigbe KO. Incidence and associated risk factors of postpartum depression in a tertiary hospital in Nigeria. *Nigerian Postgraduate Medical Journal*. 2008;15:15. | No major depression |
| Eberhard-Gran M, Eskild A, Tambs K, Schei B, Opjordsmoen S. The Edinburgh Postnatal Depression Scale: validation in a Norwegian community sample. *Nordic Journal of Psychiatry*. 2001;55:113. | No validated interview to assess major depression |
| Ekeroma AJ, Ikenasio-Thorpe B, Weeks S, Kokaua J, Puniani K, Stone P, Foliaki SA. Validation of the Edinburgh Postnatal Depression Scale (EPDS) as a screening tool for postnatal depression in Samoan and Tongan women living in New Zealand. *New Zealand Medical Journal*. 2012;125:41. | > 2 weeks between EPDS and diagnostic interview |
| Ekuklu G, Tokuc B, Eskiocak M, Berberoglu U, Saltik A. Prevalence of postpartum depression in Edirne, Turkey, and related factors. *Journal of Reproductive Medicine*. 2004;49:908. | No major depression |
| El-Ibiary SY, Hamilton SP, Abel R, Erdman CA, Robertson PA, Finley PR. A pilot study evaluating genetic and environmental factors for postpartum depression. *Innovations in Clinical Neuroscience*. 2013;10:15. | Sample selected for known distress, mental health diagnosis, or psychiatric setting |
| Elliott SA, Leverton TJ, Sanjack M, Turner H, Cowmeadow P, Hopkins J, Bushnell D. Promoting mental health after childbirth: a controlled trial of primary prevention of postnatal depression. *British Journal of Clinical Psychology*. 2000;39:223. | Sample selected for known distress, mental health diagnosis, or psychiatric setting |
| Esiwe C, Baillon S, Rajkonwar A, Lindesay J, Lo N, Dennis M. Screening for depression in older people on acute medical wards: the validity of the Edinburgh Depression Scale. *Age and Ageing*. 2016;45:554-8. | No EPDS |
| Fairbrother N, Young AH, Janssen P, Antony MM, Tucker E. Depression and anxiety during the perinatal period. *BMC Psychiatry*. 2015;15:Art 206. | Sample selected for known distress, mental health diagnosis, or psychiatric setting |
| Farhat A, Saeidi R, Mohammadzadeh A, Hesari H. Prevalence of Postpartum Depression; a longitudinal study Iranian. *Journal of Neonatology*. 2015;6:39. | No major depression |
| Fingerhut CG. Differentiating unipolar and bipolar depression in postpartum women. *Dissertation Abstracts International: Section B:* *The Sciences and Engineering*. 2016;77:No Pagination Specified. | Sample selected for known distress, mental health diagnosis, or psychiatric setting |
| Flynn HA, Sexton M, Ratliff S, Porter K, Zivin K. Comparative performance of the Edinburgh Postnatal Depression Scale and the Patient Health Questionnaire-9 in pregnant and postpartum women seeking psychiatric services. *Psychiatry Research*. 2011;187:130. | Sample selected for known distress, mental health diagnosis, or psychiatric setting |
| Gallanti AME, Rodriguez CEAM, Rodriguez IM, Sosa MA. Puerperal depression and its association with demographic and social factors, the way of resolution of pregnancy and the newborn clinical evolution. *Medula*. 2015;24:25. | No major depression |
| Gelabert E, Subira S, Plaza A, Torres A, Navarro P, Imaz ML, Valdes M, Garcia-Esteve L, Martin-Santos R. The Vulnerable Personality Style Questionnaire: psychometric properties in Spanish postpartum women. *Archives of Women's Mental Health*. 2011;14:115. | Sample selected for known distress, mental health diagnosis, or psychiatric setting |
| Gemmill AW, Leigh B, Ericksen J, Milgrom J. A survey of the clinical acceptability of screening for postnatal depression in depressed and non-depressed women. *BMC Public Health*. 2006;6:211. | No major depression |
| George C, Lalitha AR, Antony A, Kumar AV, Jacob KS. Antenatal depression in coastal South India: prevalence and risk factors in the community. *International Journal of Social Psychiatry*. 2016;62:141-7. | Sample selected for known distress, mental health diagnosis, or psychiatric setting |
| Georgiopoulos AM, Bryan TL, Wollan P, Yawn BP. Routine screening for postpartum depression. *Journal of Family Practice*. 2001;50:117. | No major depression |
| Gerardin P, Wendland J, Bodeau N, Galin A, Bialobos S, Tordjman S, Mazet P, Darbois Y, Nizard J, Dommergues M, Cohen D. Depression during pregnancy: Is the developmental impact earlier in boys? A prospective case-control study. *Journal of Clinical Psychiatry*. 2011;72:378. | Sample selected for known distress, mental health diagnosis, or psychiatric setting |
| Gerardin P. Characteristics and clinical consequences of prenatal depression. Main results of a prospective case-control study on perinatal depression from pregnancy to one year-old infant. *Neuropsychiatrie de L'enfance et de L'adolescence*. 2012;60:138. | Sample selected for known distress, mental health diagnosis, or psychiatric setting |
| Ghubash R, Abou-Saleh MT, Daradkeh TK. The validity of the Arabic Edinburgh Postnatal Depression Scale. *Social Psychiatry & Psychiatric Epidemiology*. 1997;32:474. | > 2 weeks between EPDS and diagnostic interview |
| Ghubash R, Abou-Saleh MT. Postpartum psychiatric illness in Arab culture: prevalence and psychosocial correlates. *The* *British Journal of Psychiatry*. 1997;171:65. | > 2 weeks between EPDS and diagnostic interview |
| Ginsburg GS, Barlow A, Goklish N, Hastings R, Baker EV, Mullany B, Tein JY, Walkup J. Postpartum Depression Prevention for Reservation-Based American Indians: Results from a Pilot Randomized Controlled Trial. *Child & Youth Care Forum*. 2012;41:229. | Sample selected for known distress, mental health diagnosis, or psychiatric setting |
| Goeb JL, Ferel S, Guetta J, Guibert J, Guedeney A, Coste J, Dulioust E, Jouannet P, Golse B. Assisted Reproductive Techniques when the Man is Hiv Seropositive. *Psychiatrie De L Enfant*. 2009;52:63. | No major depression |
| Gollan JK, Wisniewski SR, Luther JF, Eng HF, Dills JL, Sit D, Ciolino JD, Wisner KL. Generating an efficient version of the Edinburgh Postnatal Depression Scale in an urban obstetrical population. *Journal of Affective Disorders*. 2017;208:615-20. | No validated interview to assess major depression |
| Goutaudier N, Lopez A, Sejourne N, Denis A, Chabrol H. Premature birth: subjective and psychological experiences in the first weeks following childbirth, a mixed-methods study. *Journal of Reproductive and Infant Psychology*. 2011;29:364. | No major depression |
| Goyal D, Park VT, McNiesh S. Postpartum depression among Asian Indian mothers. *MCN: The American Journal of Maternal/Child Nursing*. 2015;40:256. | No major depression |
| Grant KA, Bautovich A, McMahon C, Reilly N, Leader L, Austin MP. Parental care and control during childhood: Associations with maternal perinatal mood disturbance and parenting stress. *Archives of Women's Mental Health*. 2012;15:297. | Could not determine eligibility^a^ |
| Grant KA, McMahon C, Austin MP, Reilly N, Leader L, Ali S. Maternal prenatal anxiety, postnatal caregiving and infants' cortisol responses to the still-face procedure. *Developmental Psychobiology*. 2009;51:625. | Could not determine eligibility^a^ |
| Grant KA, McMahon C, Reilly N, Austin MP. Maternal sensitivity moderates the impact of prenatal anxiety disorder on infant responses to the still-face procedure. *Infant Behavior & Development*. 2010;33:453. | Could not determine eligibility^a^ |
| Grigoriadis S, de Camps Meschino D, Barrons E,Bradley L, Eady A, Fishell A, Mamisachvili L, Cook GS, O'Keefe M, Romans S, Ross LE. Mood and anxiety disorders in a sample of Canadian perinatal women referred for psychiatric care. *Archives of Women's Mental Health*. 2011;14:325. | Sample selected for known distress, mental health diagnosis, or psychiatric setting |
| Guedeney N, Fermanian J. Validation study of the French version of the Edinburgh Postnatal Depression Scale (EPDS): new results about use and psychometric properties. *European Psychiatry: The Journal of The Association of European Psychiatrists*. 1998;13:83. | Sample selected for known distress, mental health diagnosis, or psychiatric setting |
| Guintivano J, Sullivan PF, Stuebe AM, Penders T, Thorp J, Rubinow DR, Meltzer-Brody S. Adverse life events, psychiatric history, and biological predictors of postpartum depression in an ethnically diverse sample of postpartum women. *Psychological Medicine*. 2018;48:1190-200. | Sample selected for known distress, mental health diagnosis, or psychiatric setting |
| Gutierrez-Zotes A, Labad J, Martin-Santos R, Garcia-Esteve L, Gelabert E, Jover M, Guillamat R, Mayoral F, Gornemann I, Canellas F, Gratacos M, Guitart M, Roca M, Costas J, Ivorra JL, Navines R, de Diego-Otero Y, Vilella E, Sanjuan J. Coping strategies and postpartum depressive symptoms: A structural equation modelling approach. *European Psychiatry: The Journal of The Association of European Psychiatrists*. 2015;30:701. | Sample selected for known distress, mental health diagnosis, or psychiatric setting |
| Gutiérrez-Zotes A, Labad J, Martín-Santos R, García-Esteve L, Gelabert E, Jover M, Guillamat R, Mayoral F, Gornemann I, Canellas F, Gratacós M. Coping strategies for postpartum depression: a multi-centric study of 1626 women. *Archives of Women's Mental Health*. 2016;19:455-61. | Sample selected for known distress, mental health diagnosis, or psychiatric setting |
| Gutierrez-Zotes JA, Farnos A, Vilella E, Labad J. Higher psychoticism as a predictor of thoughts of harming one's infant in postpartum women: a prospective study. *Comprehensive Psychiatry*. 2013;54:1124. | Sample selected for known distress, mental health diagnosis, or psychiatric setting |
| Hamdan A, Tamim H. Psychosocial risk and protective factors for postpartum depression in the United Arab Emirates. *Archives of Women's Mental Health*. 2011;14:125. | Sample selected for known distress, mental health diagnosis, or psychiatric setting |
| Hamdan A, Tamim H. The relationship between postpartum depression and breastfeeding. *International Journal of Psychiatry in Medicine*. 2012;43:243. | Sample selected for known distress, mental health diagnosis, or psychiatric setting |
| Hanusa BH, Scholle SH, Haskett RF, Spadaro K, Wisner KL. Screening for depression in the postpartum period: a comparison of three instruments. *Journal of Women's Health*. 2008;17:585. | Sample selected for known distress, mental health diagnosis, or psychiatric setting |
| Harris B, Huckle P, Thomas R, Johns S, Fung H. The use of rating scales to identify post-natal depression. *The* *British Journal of Psychiatry*. 1989;154:813. | Could not determine eligibility^a^ |
| Harris B, Othman S, Davies JA, Weppner GJ, Richards CJ, Newcombe RG, Lazarus JH, Parkes AB, Hall R, Phillips DI. Association between postpartum thyroid dysfunction and thyroid antibodies and depression. *BMJ*. 1992;305:152. | No validated interview to assess major depression |
| Harvey ST, Pun PK. Analysis of positive Edinburgh depression scale referrals to a consultation liaison psychiatry service in a two-year period. *International Journal of Mental Health Nursing*. 2007;16:161. | Sample selected for known distress, mental health diagnosis, or psychiatric setting |
| Hatton DC, HarrisonHohner J, Matarazzo J, Edwards P, Lewy A, Davis L. Missed antenatal depression among high risk women: A secondary analysis. *Archives of Women's Mental Health*. 2007;10:121. | No validated interview to assess major depression |
| Henshaw C, Foreman D, Cox J. Postnatal blues: a risk factor for postnatal depression. *Journal of Psychosomatic Obstetrics & Gynecology*. 2004;25:267. | Sample selected for known distress, mental health diagnosis, or psychiatric setting |
| Herz E, Thoma M, Umek W, Gruber K, Linzmayer L, Walcher W, Philipp T, Putz M. Non-psychotic post-partum depression. *Geburtshilfe und Frauenheilkunde*. 1997;57:282. | No major depression |
| Holden JM. Postnatal depression: its nature, effects, and identification using the Edinburgh Postnatal Depression scale. *Birth*. 1991;18:211. | No original data |
| Holt WJ. The detection of postnatal depression in general practice using the Edinburgh postnatal depression scale. *New Zealand Medical Journal*. 1995;108:57. | > 2 weeks between EPDS and diagnostic interview |
| Howard LM, Flach C, Mehay A, Sharp D, Tylee A. The prevalence of suicidal ideation identified by the Edinburgh Postnatal Depression Scale in postpartum women in primary care: findings from the RESPOND trial. *BMC Pregnancy & Childbirth*. 2011;11:57. | Sample selected for known distress, mental health diagnosis, or psychiatric setting |
| Huang J, Zhang L, He M, Qiang X, Xiao X, Huang S, Zhang D, Tang M. Comprehensive evaluation of postpartum depression and correlations between postpartum depression and serum levels of homocysteine in Chinese women. *Zhong Nan da Xue Xue Bao. Yi Xue Ban Journal of Central South University Medical Sciences*. 2015;40:311. | No validated interview to assess major depression |
| Huang YC, Mathers NJ. Postnatal depression and the experience of South Asian marriage migrant women in Taiwan: survey and semi-structured interview study. *International Journal of Nursing Studies*. 2008;45:924. | No major depression |
| Hudson C, Spry E, Borschmann R, Becker D, Moran P, Olsson C, Coffey C, Romaniuk H, Bayer JK, Patton GC. Preconception personality disorder and antenatal maternal mental health: A population-based cohort study. *Journal of Affective Disorders*. 2017;209:169-76. | No major depression |
| Husain N, Cruickshank K, Husain M, Khan S, Tomenson B, Rahman A. Social stress and depression during pregnancy and in the postnatal period in British Pakistani mothers: a cohort study. *Journal of Affective Disorders*. 2012;140:268. | Could not determine eligibility^a^ |
| Husain N, Kiran T, Sumra A, Naeem Zafar S, Ur Rahman R, Jafri F, Ansari S, Husain M, Adelekan ML, Bashir Chaudhry I. Detecting maternal depression in a low-income country: comparison of the self-reporting questionnaire and the edinburgh postnatal depression scale. *Journal of Tropical Pediatrics*. 2014;60:129. | Could not determine eligibility^a^ |
| Ibanez G, Bernard JY, Rondet C, Peyre H, Forhan A, Kaminski M, Cubizolles MJS, EDEN Mother-Child Cohort Study Group. Effects of antenatal maternal depression and anxiety on children's early cognitive development: A prospective cohort study. *PLoS One*. 2015;10:Art e0135849. | No major depression |
| Idaiani S, Kusumawardani N, Isfandari S. The determinants of perinatal depression (PND) in Tebet Merdeka, Jakarta and Sindangbarang, Bogor Indonesia. *Asean Journal of Psychiatry*. 2018;19:54 | No major depression |
| Ikeda M, Hayashi M, Kamibeppu K. The relationship between attachment style and postpartum depression. *Attachment & Human Development*. 2014;16:557. | > 2 weeks between EPDS and diagnostic interview |
| Inglis AJ, Hippman CL, Carrion PB, Honer WG, Austin JC. Mania and depression in the perinatal period among women with a history of major depressive disorders. *Archives of Women's Mental Health*. 2014;17:137. | Sample selected for known distress, mental health diagnosis, or psychiatric setting |
| Jadresic E, Araya R, Jara C. Validation of the Edinburgh Postnatal Depression Scale (EPDS) in Chilean postpartum women. *Journal of Psychosomatic Obstetrics & Gynecology*. 1995;16:187. | No validated interview to assess major depression |
| Jaju S, Al Kharusi L, Gowri V. Antenatal prevalence of fear associated with childbirth and depressed mood in primigravid women. *Indian Journal of Psychiatry*. 2015;57:158. | Sample selected for known distress, mental health diagnosis, or psychiatric setting |
| Jardri R, Maron M, Pelta J, Thomas P, Codaccioni X, Goudemand M, Delion P. Impact of midwives' training on postnatal depression screening in the first week post delivery: a quality improvement report. *Midwifery*. 2010;26:622. | > 2 weeks between EPDS and diagnostic interview |
| Ji S, Long Q, Newport DJ, Na H, Knight B, Zach EB, Morris NJ, Kutner M, Stowe ZN. Validity of depression rating scales during pregnancy and the postpartum period: impact of trimester and parity. *Journal of Psychiatric Research*. 2011;45:213. | Sample selected for known distress, mental health diagnosis, or psychiatric setting |
| Josefsson A, Larsson C, Sydsjo G, Nylander PO. Temperament and character in women with postpartum depression. *Archives of Women's Mental Health*. 2007;10:3. | Sample selected for known distress, mental health diagnosis, or psychiatric setting |
| Keshavarzi F, Yazdchi K, Rahimi M, Rezaei M, Farnia V, Davarinejad O, Abdoli N, Jalili M. Post partum depression and thyroid function. *Iranian Journal of Psychiatry*. 2011;6:117. | No major depression |
| Kim K, Hong JP, Cho MJ, Fava M, Mischoulon D, Lee DW, Heo JY, Jeon HJ. Loss of sexual interest and premenstrual mood change in women with postpartum versus non-postpartum depression: A nationwide community sample of Korean adults. *Journal of Affective Disorders*. 2016;191:222-9. | No EPDS |
| Kingston D, Austin MP, van Zanten SV, Harvalik P, Giallo R, McDonald SD, MacQueen G, Vermeyden L, Lasiuk G, Sword W, Biringer A. Pregnant women’s views on the feasibility and acceptability of web-based mental health e-screening versus paper-based screening: a randomized controlled trial. *Journal of Medical Internet Research*. 2017;19:e88. | Could not determine eligibility^a^ |
| Kirkan TS, Aydin N, Yazici E, Akcali Aslan P, Acemoglu H, Daloglu AG. The depression in women in pregnancy and postpartum period: A follow-up study. *International Journal of Social Psychiatry*. 2015;61:343. | Sample selected for known distress, mental health diagnosis, or psychiatric setting |
| Klier CM, Muzik M, Dervic K, Mossaheb N, Benesch T, Ulm B, Zeller M. The role of estrogen and progesterone in depression after birth. *Journal of Psychiatric Research*. 2007;41:273. | > 2 weeks between EPDS and diagnostic interview |
| Knight J, Martin J, Patil A. Principal Component Analysis of EPDS Questions to Identify Trends in Depressive Symptoms Among At-Risk Populations. *Obstetrics & Gynecology*. 2016;127:96S-7S. | No validated interview to assess major depression |
| Knorring LV. Review of Depression in women with focus on the postpartum period. *Nordic Journal of Psychiatry*. 2003;57:390. | No validated interview to assess major depression |
| Kohlhoff J, Hickinbotham R, Knox C, Roach V, Barnett Am B. Antenatal psychosocial assessment and depression screening in a private hospital. *Australian & New Zealand Journal of Obstetrics & Gynaecology*. 2016;56:173. | No major depression |
| Koss J, Bidzan M, Smutek J, Bidzan L. Influence of Perinatal Depression on Labor-Associated Fear and Emotional Attachment to the Child in High-Risk Pregnancies and the First Days After Delivery. *Medical Science Monitor*. 2016;22:1028. | No major depression |
| Koukounari A, Stringaris A, Maughan B. Pathways from maternal depression to young adult offspring depression: an exploratory longitudinal mediation analysis. *International Journal of Methods in Psychiatric Research*. 2017;26:e1520. | No major depression |
| Lai BP, Tang AK, Lee DT, Yip AS, Chung TK. Detecting postnatal depression in Chinese men: a comparison of three instruments. *Psychiatry Research*. 2010;180:80. | No pregnant or postpartum women |
| Lau Y, Wang Y, Yin L, Chan KS, Guo X. Validation of the Mainland Chinese version of the Edinburgh Postnatal Depression Scale in Chengdu mothers. *International Journal of Nursing Studies*. 2010;47:1139. | Could not determine eligibility^a^ |
| Lawrie TA, Hofmeyr GJ, de Jager M, Berk M. Validation of the Edinburgh Postnatal Depression Scale on a cohort of South African women. *South African Medical Journal. Suid-Afrikaanse Tydskrif Vir Geneeskunde*. 1998;88:1340. | No validated interview to assess major depression |
| Lee DT, Wong CK, Ungvari GS, Cheung LP, Haines CJ, Chung TK. Screening psychiatric morbidity after miscarriage: application of the 30-item General Health Questionnaire and the Edinburgh Postnatal Depression Scale. *Psychosomatic Medicine*. 1997;59:207. | No pregnant or postpartum women |
| Lee DT, Yip AS, Chan SS, Tsui MH, Wong WS, Chung TK. Postdelivery screening for postpartum depression. *Psychosomatic Medicine*. 2003;65:357. | No major depression |
| Lee DT, Yip AS, Chiu HF, Chung TK. Screening for postnatal depression using the double-test strategy. *Psychosomatic Medicine*. 2000;62:258. | No major depression |
| Lee DT, Yip AS, Chiu HF, Leung TY, Chung TK. Screening for postnatal depression: are specific instruments mandatory? *Journal of Affective Disorders*. 2001;63:233. | No major depression |
| Lee DT, Yip SK, Chiu HF, Leung TY, Chan KP, Chau IO, Leung HC, Chung TK. Detecting postnatal depression in Chinese women. Validation of the Chinese version of the Edinburgh Postnatal Depression Scale. *The* *British Journal of Psychiatry*. 1998;172:433. | No validated interview to assess major depression |
| Leverton TJ, Elliott SA. Is the EPDS a magic wand ?: 1. A comparison of the Edinburgh Postnatal Depression Scale and health visitor report as predictors of diagnosis on the Present State Examination. *Journal of Reproductive and Infant Psychology*. 2000;18:279. | Sample selected for known distress, mental health diagnosis, or psychiatric setting |
| Lewis BA, Gjerdingen DK, Avery MD, Guo H, Sirard JR, Bonikowske AR, Marcus BH. Examination of a telephone-based exercise intervention for the prevention of postpartum depression: design, methodology, and baseline data from The Healthy Mom study. *Contemporary Clinical Trials*. 2012;33:1150. | No major depression |
| Lewis BA, Gjerdingen DK, Avery MD, Sirard JR, Guo H, Schuver K, Marcus BH. A randomized trial examining a physical activity intervention for the prevention of postpartum depression: the healthy mom trial. *Mental Health and Physical Activity*. 2014;7:42-9. | Sample selected for known distress, mental health diagnosis, or psychiatric setting |
| Littlewood E, Ali S, Ansell P, Dyson L, Gascoyne S, Hewitt C, Keding A, Mann R, McMillan D, Morgan D, Swan K. Identification of depression in women during pregnancy and the early postnatal period using the Whooley questions and the Edinburgh Postnatal Depression Scale: protocol for the Born and Bred in Yorkshire: PeriNatal Depression Diagnostic Accuracy (BaBY PaNDA) study. *BMJ Open*. 2016;6:e011223. | No original data |
| Logsdon MC, Myers JA. Comparative performance of two depression screening instruments in adolescent mothers. *Journal of Women's Health*. 2010;19:1123. | No adults |
| Lukasik A, Blaszczyk K, Wojcieszyn M, Belowska A. Characteristic of affective disorders of the first week of puerperium. *Ginekologia polska*. 2003;74:1194. | No validated interview to assess major depression |
| Lundh W, Gyllang C. Use of the Edinburgh Postnatal Depression Scale in some Swedish child health care centres. *Scandinavian Journal of Caring Sciences*. 1993;7:149. | No validated interview to assess major depression |
| Lydsdottir LB, Howard LM, Olafsdottir H, Thome M, Tyrfingsson P, Sigurdsson JF. The mental health characteristics of pregnant women with depressive symptoms identified by the Edinburgh Postnatal Depression Scale. *Journal of Clinical Psychiatry*. 2014;75:393. | > 2 weeks between EPDS and diagnostic interview |
| Mallett P, Andrew M, Hunter C, Smith J, Richards C, Othman S, Lazarus J, Harris B. Cognitive function, thyroid status and postpartum depression. *Acta Psychiatrica Scandinavica*. 1995;91:243. | No validated interview to assess major depression |
| Maloney DM. Postnatal depression: a study of mothers in the metropolitan area of Perth, Western Australia. *Journal - Australian College of Midwives*. 1998;11:18. | No major depression |
| Mao HJ, Li HJ, Chiu H, Chan WC, Chen SL. Effectiveness of antenatal emotional self-management training program in prevention of postnatal depression in Chinese women. *Perspectives in Psychiatric Care*. 2012;48:218. | Sample selected for known distress, mental health diagnosis, or psychiatric setting |
| Marley JV, Kotz J, Engelke C, Williams M, Stephen D, Coutinho S. Validity and acceptability of kimberley mum’s mood scale to screen for perinatal anxiety and depression in remote aboriginal health care settings. *PLoS One*. 2017;12:e0168969. | No EPDS |
| MartinSantos R, Gelabert E, Subira S, Gutierrezzotes A, Langorh K, Jover M, Torrens M, Guillamat R, Mayoral F, Canellas F, Iborra JL, Gratacos M, Costas J, Gornemann I, Navines R, Gutart M, Roca M, De Frutos R, Vilella E, Valdes M, Garcia Esteve L, Sanjuan J. Research Letter: Is neuroticism a risk factor for postpartum depression? *Psychological Medicine*. 2012;42:1559. | No original data |
| Mason L, Poole H. Healthcare professionals' views of screening for postnatal depression. *Community Practitioner*. 2008;81:30. | No pregnant or postpartum women |
| Matijasevich A, Munhoz TN, Tavares BF, Barbosa AP, da Silva DM, Abitante MS, Dall'Agnol TA, Santos IS. Validation of the Edinburgh Postnatal Depression Scale (EPDS) for screening of major depressive episode among adults from the general population. *BMC Psychiatry*. 2014;14:284. | No pregnant or postpartum women |
| Matthews J, Huberty JL, Leiferman JA, McClain D, Larkey LK. Perceptions, uses of, and interests in complementary health care approaches in depressed pregnant women: the PAW survey. *Journal of Evidence-Based Complementary & Alternative Medicine*. 2017;22:81-95. | No major depression |
| Matthey S, Valenti B, Souter K, Ross-Hamid C. Comparison of four self-report measures and a generic mood question to screen for anxiety during pregnancy in English-speaking women. *Journal of Affective Disorders*. 2013;148:347. | Sample selected for known distress, mental health diagnosis, or psychiatric setting |
| Matthey S. Differentiating between Transient and Enduring distress on the Edinburgh Depression Scale within screening contexts. *Journal of Affective Disorders*. 2016;196:252. | Sample selected for known distress, mental health diagnosis, or psychiatric setting |
| Matthey S. Does an early postpartum Edinburgh Postnatal Depression Scale (EPDS) really detect the majority of women with elevated EPDS scores at 16-weeks postpartum? *Archives of Women's Mental Health*. 2017;20:811-2. | No original data |
| Matthey S. Using the Edinburgh Postnatal Depression Scale to screen for anxiety disorders. *Depression & Anxiety*. 2008;25:926. | No pregnant or postpartum women |
| Mauri M, Banti S, Borri C, Rambelli C, Ramacciotti D, Oppo A, Montagnani MS, Camilleri V, Cortopassi S, Cianelli E, Ciberti A, Mariani MG, Cassano GB. Depressive Symptomatology in Pregnancy Detected with Epds: the Problem of False Positive. *European Psychiatry*. 2010;25. | Sample selected for known distress, mental health diagnosis, or psychiatric setting |
| Mazhari S, Nakhaee N. Validation of the Edinburgh Postnatal Depression Scale in an Iranian sample. *Archives of Women's Mental Health*. 2007;10:293. | No validated interview to assess major depression |
| Mazzeo SE, SlofOp't Landt MC, Jones I, Mitchell K, Kendler KS, Neale MC, Aggen SH, Bulik CM. Associations among postpartum depression, eating disorders, and perfectionism in a population-based sample of adult women. *International Journal of Eating Disorders*. 2006;39:202. | No major depression |
| McMahon CA, Boivin J, Gibson FL, Hammarberg K, Wynter K, Fisher JR. Older maternal age and major depressive episodes in the first two years after birth: Findings from the Parental Age and Transition to Parenthood Australia (PATPA) study. *Journal of Affective Disorders*. 2015;175:454. | No major depression |
| Meltzer-Brody S, Zerwas S, Leserman J, Holle AV, Regis T, Bulik C. Eating disorders and trauma history in women with perinatal depression. *Journal of Women's Health*. 2011;20:863. | Sample selected for known distress, mental health diagnosis, or psychiatric setting |
| Meuti V, Aceti F, Giacchetti N, Carluccio GM, Zaccagni M, Marini I, Giancola O, Ciolli P, Biondi M. Perinatal Depression and Patterns of Attachment: A Critical Risk Factor? *Depression Research and Treatment*. 2015;2015:105012. | Sample selected for known distress, mental health diagnosis, or psychiatric setting |
| Milgrom J, Gemmill AW, Ericksen J, Burrows G, Buist A, Reece J. Treatment of postnatal depression with cognitive behavioural therapy, sertraline and combination therapy: A randomised controlled trial. *Australian and New Zealand Journal of Psychiatry*. 2015;49:236. | Sample selected for known distress, mental health diagnosis, or psychiatric setting |
| Miller L, Gur M, Shanok A, Weissman M. Interpersonal psychotherapy with pregnant adolescents: two pilot studies. *Journal of Child Psychology & Psychiatry & Allied Disciplines*. 2008;49:733. | No adults |
| Mirabella F, Michielin P, Piacentini D, Veltro F, Barbano G, Cattaneo M, Palumbo G, Gigantesco A. Effectiveness of a postnatal psychological treatment for women who had screened positive for depression. *Rivista di Psichiatria*. 2016;51:260-9. | Sample selected for known distress, mental health diagnosis, or psychiatric setting |
| Moayedoddin A, Moser D, Nanzer N. The impact of brief psychotherapy centred on parenthood on the anxio-depressive symptoms of mothers during the perinatal period. *Swiss Medical Weekly*. 2013;143:w13769. | Sample selected for known distress, mental health diagnosis, or psychiatric setting |
| Mochache K, Mathai M, Gachuno O, Vander Stoep A, Kumar M. Depression during pregnancy and preterm delivery: a prospective cohort study among women attending antenatal clinic at Pumwani Maternity Hospital. *Annals of General Psychiatry*. 2018;17:31. | Sample selected for known distress, mental health diagnosis, or psychiatric setting |
| Murray D, Cox JL, Chapman G, Jones P. Childbirth: life event or start of a long-term difficulty? Further data from the Stoke-on-Trent controlled study of postnatal depression. *The* *British Journal of Psychiatry*. 1995;166:595. | No validated interview to assess major depression |
| Murray D, Cox JL. Screening for depression during pregnancy with the Edinburgh Depression Scale (EPDS). *Journal of Reproductive and Infant Psychology*. 1990;8:99. | No validated interview to assess major depression |
| Murray L, Carothers AD. The validation of the Edinburgh Post-natal Depression Scale on a community sample. *The* *British Journal of Psychiatry*. 1990;157:288. | No validated interview to assess major depression |
| O'Mahen H, Himle JA, Fedock G, Henshaw E, Flynn H. A pilot randomized controlled trial of cognitive behavioral therapy for perinatal depression adapted for women with low incomes. *Depression and Anxiety*. 2013;30:679. | Sample selected for known distress, mental health diagnosis, or psychiatric setting |
| O'Neill T. Postnatal depression--aetiological factors. *Irish Medical Journal*. 1990;83:17. | > 2 weeks between EPDS and diagnostic interview |
| Ortiz Collado MA, Saez M, Favrod J, Hatem M. Antenatal psychosomatic programming to reduce postpartum depression risk and improve childbirth outcomes: a randomized controlled trial in Spain and France. *BMC Pregnancy & Childbirth*. 2014;14:22. | No major depression |
| Owoeye AO, Aina OF, Morakinyo O. Risk factors of postpartum depression and EPDS scores in a group of Nigerian women. *Tropical Doctor*. 2006;36:100. | Sample selected for known distress, mental health diagnosis, or psychiatric setting |
| Parker G, Hegarty B, Granville-Smith I, Ho J, Paterson A, Gokiert A, Hadzi-Pavlovic D. Is essential fatty acid status in late pregnancy predictive of post-natal depression? *Acta Psychiatrica Scandinavica*. 2015;131:148. | Sample selected for known distress, mental health diagnosis, or psychiatric setting |
| Parker GB, Hegarty B, Paterson A, Hadzi-Pavlovic D, Granville-Smith I, Gokiert A. Predictors of post-natal depression are shaped distinctly by the measure of 'depression'. *Journal of Affective Disorders*. 2015;173:239. | Sample selected for known distress, mental health diagnosis, or psychiatric setting |
| Patton GC, Romaniuk H, Spry E, Coffey C, Olsson C, Doyle LW, Oats J, Hearps S, Carlin JB, Brown S. Prediction of perinatal depression from adolescence and before conception (VIHCS): 20-year prospective cohort study. *Lancet*. 2015;386:875. | No major depression |
| Peindl KS, Wisner KL, Hanusa BH. Identifying depression in the first postpartum year: guidelines for office-based screening and referral. *Journal of Affective Disorders*. 2004;80:37. | Sample selected for known distress, mental health diagnosis, or psychiatric setting |
| Petri E, Palagini L, Bacci O, Borri C, Teristi V, Corezzi C, Faraoni S, Antonelli P, Cargioli C, Banti S, Perugi G. Maternal–foetal attachment independently predicts the quality of maternal–infant bonding and post-partum psychopathology. *The Journal of Maternal-Fetal & Neonatal Medicine*. 2018;31:3153-9. | Sample selected for known distress, mental health diagnosis, or psychiatric setting |
| Pham D, Cormick G, Amyx MM, Gibbons L, Doty M, Brown A, Norwood A, Daray FM, Althabe F, Belizán JM. Factors associated with postpartum depression in women from low socioeconomic level in Argentina: A hierarchical model approach. *Journal of Affective Disorders.* 2018;227:731-8. | No major depression |
| Phillips J, Sharpe L, Nemeth D. Maternal psychopathology and outcomes of a residential mother-infant intervention for unsettled infant behaviour. *Australian & New Zealand Journal of Psychiatry*. 2010;44:280. | > 2 weeks between EPDS and diagnostic interview |
| Piacentini D, Leveni D, Primerano G, Cattaneo M, Volpi L, Biffi G, Mirabella F. Prevalence and risk factors of postnatal depression among women attending antenatal courses. *Epidemiologia Epsichiatria Sociale*. 2009;18:214. | > 2 weeks between EPDS and diagnostic interview |
| Pitanupong J, Liabsuetrakul T, Vittayanont A. Validation of the Thai Edinburgh Postnatal Depression Scale for screening postpartum depression. *Psychiatry Research*. 2007;149:253. | No validated interview to assess major depression |
| Pollock JI, Manaseki-Holland S, Patel V. Detection of depression in women of child-bearing age in non-Western cultures: a comparison of the Edinburgh Postnatal Depression Scale and the Self-Reporting Questionnaire-20 in Mongolia. *Journal of Affective Disorders*. 2006;92:267. | No adults |
| Quispel C, Schneider TA, Hoogendijk WJ, Bonsel GJ, Lambregtse-van den Berg MP. Successful five-item triage for the broad spectrum of mental disorders in pregnancy–a validation study. *BMC Pregnancy Childbirth*. 2015;15:51. | No major depression cases |
| Reck C, Stehle E, Reinig K, Mundt C. Maternity blues as a predictor of DSM-IV depression and anxiety disorders in the first three months postpartum. *Journal of Affective Disorders*. 2009;113:77. | Sample selected for known distress, mental health diagnosis, or psychiatric setting |
| Reck C, Struben K, Backenstrass M, Stefenelli U, Reinig K, Fuchs T, Sohn C, Mundt C. Prevalence, onset and comorbidity of postpartum anxiety and depressive disorders. *Acta Psychiatrica Scandinavica*. 2008;118:459. | > 2 weeks between EPDS and diagnostic interview |
| Regmi S, Sligl W, Carter D, Grut W, Seear M. A controlled study of postpartum depression among Nepalese women: validation of the Edinburgh Postpartum Depression Scale in Kathmandu. *Tropical Medicine & International Health*. 2002;7:378. | No major depression |
| Robakis TK, Williams KE, Crowe S, Kenna H, Gannon J, Rasgon NL. Optimistic outlook regarding maternity protects against depressive symptoms postpartum. *Archives of Women's Mental Health*. 2015;18:197. | No validated interview to assess major depression |
| Roca A, Imaz ML, Torres A, Plaza A, Subira S, Valdes M, Martin-Santos R, Garcia-Esteve L. Unplanned pregnancy and discontinuation of SSRIs in pregnant women with previously treated affective disorder. *Journal of Affective Disorders*. 2013;150:807. | Sample selected for known distress, mental health diagnosis, or psychiatric setting |
| Rojas G, Fritsch R, Solis J, Gonzalez M, Guajardo V, Araya R. Quality of life of women depressed in the post-partum period. *Revista Medica de Chile*. 2006;134:713. | Sample selected for known distress, mental health diagnosis, or psychiatric setting |
| Rubertsson C, Borjesson K, Berglund A, Josefsson A, Sydsjo G. The Swedish validation of Edinburgh Postnatal Depression Scale (EPDS) during pregnancy. *Nordic Journal of Psychiatry*. 2011;65:414. | No validated interview to assess major depression |
| Saleh ES, El-Bahei W, El-Hadidy MA, Zayed A. Predictors of postpartum depression in a sample of Egyptian women. *Neuropsychiatric Disease and Treatment*. 2012;9:Art 15. | No EPDS |
| Sanjuan J, MartinSantos R, GarciaEsteve L, Carot JM, Guillamat R, GutierrezZotes A, Gornemann I, Canellas F, BacaGarcia E, Jover M, Navines R, Valles V, Vilella E, de Diego Y, Castro JA, Ivorra JL, Gelabert E, Guitart M, Labad A, Mayoral F, Roca M, Gratacos M, Costas J, van Os J, de Frutos R. Mood changes after delivery: Role of the serotonin transporter gene. *The British Journal of Psychiatry*. 2008;193:383. | Sample selected for known distress, mental health diagnosis, or psychiatric setting |
| Santos IS, Matijasevich A, Tavares BF, Barros AJ, Botelho IP, Lapolli C, Magalhaes PV, Barbosa AP, Barros FC. Validation of the Edinburgh Postnatal Depression Scale (EPDS) in a sample of mothers from the 2004 Pelotas Birth Cohort Study. *Cadernos de Saude Publica*. 2007;23:2577. | No validated interview to assess major depression |
| Santos IS, Matijasevich A, Tavares BF, da Cruz Lima AC, Riegel RE, Lopes BC. Comparing validity of Edinburgh scale and SRQ20 in screening for post-partum depression. *Clinical Practice & Epidemiology in Mental Health [Electronic Resource]: CP & EMH*. 2007;3:18. | No validated interview to assess major depression |
| Santos IS, Tavares BF, Munhoz TN, Manzolli P, de Ávila GB, Jannke E, Matijasevich A. Patient health questionnaire-9 versus Edinburgh postnatal depression scale in screening for major depressive episodes: a cross-sectional population-based study. *BMC Research Notes*. 2016;9:453. | No pregnant or postpartum women |
| Savarimuthu RJ, Ezhilarasu P, Charles H, Antonisamy B, Kurian S, Jacob KS. Post-partum depression in the community: a qualitative study from rural South India. *International Journal of Social Psychiatry*. 2010;56:94. | Could not determine eligibility^a^ |
| Sejourne N, Alba J, Onorrus M, Goutaudier N, Chabrol H. Intergenerational transmission of postpartum depression. *Journal of Reproductive and Infant Psychology*. 2011;29:115. | No validated interview to assess major depression |
| Seth S, Lewis AJ, Saffery R, Lappas M, Galbally M. Maternal Prenatal Mental Health and Placental 11 beta-HSD2 Gene Expression: Initial Findings from the Mercy Pregnancy and Emotional Wellbeing Study. *International Journal of Molecular Sciences*. 2015;16:27482. | No major depression |
| Silver M, Moore CM, Villamarin V, Jaitly N, Hall JE, Rothschild AJ, Deligiannidis KM. White matter integrity in medication-free women with peripartum depression: a tract-based spatial statistics study. *Neuropsychopharmacology*. 2018;43:1573. | Sample selected for known distress, mental health diagnosis, or psychiatric setting |
| Silverman ME, Reichenberg A, Savitz DA, Cnattingius S, Lichtenstein P, Hultman CM, Larsson H, Sandin S. The risk factors for postpartum depression: A population‐based study. *Depression and Anxiety*. 2017;34:178-87.. | No EPDS |
| Simpson W, Glazer M, Michalski N, Steiner M, Frey BN. Comparative efficacy of the generalized anxiety disorder 7-item scale and the Edinburgh Postnatal Depression Scale as screening tools for generalized anxiety disorder in pregnancy and the postpartum period. *Canadian Journal of Psychiatry - Revue Canadienne de Psychiatrie*. 2014;59:434. | Sample selected for known distress, mental health diagnosis, or psychiatric setting |
| Sit DK, Flint C, Svidergol D, White J, Wimer M, Bish B, Wisner KL. Best practices: an emerging best practice model for perinatal depression care. *Psychiatric Services*. 2009;60:1429. | No validated interview to assess major depression |
| Slade P, Morrell CJ, Rigby A, Ricci K, Spittlehouse J, Brugha TS. Postnatal women's experiences of management of depressive symptoms: a qualitative study. *British Journal of General Practice*. 2010;60. | No major depression |
| Smith-Nielsen J, Steele H, Mehlhase H, Cordes K, Steele M, Harder S, Væver MS. Links among high EPDS scores, state of mind regarding attachment, and symptoms of personality disorder. *Journal of Personality Disorders*. 2015;29:771-93. | Sample selected for known distress, mental health diagnosis, or psychiatric setting |
| Spinelli MG, Endicott J, Goetz RR, Segre LS. Reanalysis of efficacy of interpersonal psychotherapy for antepartum depression versus parenting education program: initial severity of depression as a predictor of treatment outcome. *The Journal of Clinical Psychiatry*. 2016;77:535-40. | Sample selected for known distress, mental health diagnosis, or psychiatric setting |
| Sundaram S, Harman JS, Cook RL. Maternal morbidities and postpartum depression: An analysis using the 2007 and 2008 pregnancy risk assessment monitoring system. *Women's Health Issues*. Jul 2014;24:e381. | No EPDS |
| Sutter-Dallay AL, Giaconne-Marcesche V, Glatigny-Dallay E, Verdoux H. Women with anxiety disorders during pregnancy are at increased risk of intense postnatal depressive symptoms: a prospective survey of the MATQUID cohort. *European Psychiatry*. 2004;19:459. | > 2 weeks between EPDS and diagnostic interview |
| Tam LW, Newton RP, Dern M, Parry BL. Screening women for postpartum depression at well baby visits: resistance encountered and recommendations. *Archives of Women's Mental Health*. 2002;5:79. | Sample selected for known distress, mental health diagnosis, or psychiatric setting |
| Tan EC, Chua TE, Lee TMY, Tan HS, Ting JLY, Chen HY. Case-control study of glucocorticoid receptor and corticotrophin-releasing hormone receptor gene variants and risk of perinatal depression. *BMC Pregnancy and Childbirth*. 2015;15:283. | No major depression |
| Tang Y,Shi S, Lu W, Chen Y, Wang Q, Zhu Y, Yang J, Yu W, Luo J, Cheng UN. Prenatal psychological prevention trial on postpartum anxiety and depression. *Chinese Mental Health Journal*. 2009;23:83. | Could not determine eligibility^a^ |
| Tavaragi MS, Patil R, Desai M, Arunkumar C. Prevalence Study of Postpartum Depression and its Correlation with Socio-Demographic Variables at a Tertiary Care Hospital, KIMS, Huballi Indian. *Journal of Psychiatry*. 2018;60:133. | No major depression |
| Teng HW, Hsu CS, Shih SM, Lu ML, Pan JJ, Shen WW. Screening postpartum depression with the Taiwanese version of the Edinburgh Postnatal Depression scale. *Comprehensive Psychiatry*. 2005;46:261. | Could not determine eligibility^a^ |
| Tesfaye M, Hanlon C, Wondimagegn D, Alem A. Detecting postnatal common mental disorders in Addis Ababa, Ethiopia: validation of the Edinburgh Postnatal Depression Scale and Kessler Scales. *Journal of Affective Disorders*. 2010;122:102. | No validated interview to assess major depression |
| Thangavelautham Suhitharan TP, Chen H, Assam PN, Sultana R, Han NL, Tan EC, Sng BL. Investigating analgesic and psychological factors associated with risk of postpartum depression development: a case–control study. *Neuropsychiatric Disease and Treatment*. 2016;12:1333. | Sample selected for known distress, mental health diagnosis, or psychiatric setting |
| Tharner A, Luijk MPCM, van IJzendoorn MH, BakermansKranenburg MJ, Jaddoe VWV, Hofman A, Verhulst FC, Tiemeier H. Maternal lifetime history of depression and depressive symptoms in the prenatal and early postnatal period do not predict infant-mother attachment quality in a large, population-based Dutch cohort study. *Attachment & Human Development*. 2012;14:63. | > 2 weeks between EPDS and diagnostic interview |
| Thorpe K. A study of the use of the Edinburgh Postnatal Depression Scale with parent groups outside the postpartum period. *Journal of Reproductive and Infant Psychology*. 1993;11:119. | No pregnant or postpartum women |
| Tietz A, Zietlow AL, Reck C. Maternal bonding in mothers with postpartum anxiety disorder: the crucial role of subclinical depressive symptoms and maternal avoidance behaviour. *Archives of Women's Mental Health*. 2014;17:433. | Sample selected for known distress, mental health diagnosis, or psychiatric setting |
| Trevillion K, Domoney J, Pickles A, Bick D, Byford S, Heslin M, Milgrom J, Mycroft R, Pariante C, Ryan E, Hunter M. Depression: an exploratory parallel-group randomised controlled trial of Antenatal guided self help for WomeN (DAWN): study protocol for a randomised controlled trial. *Trials*. 2016;17:503. | No original data |
| Ueda M, Yamashita H, Yoshida K. Impact of infant health problems on postnatal depression: pilot study to evaluate a health visiting system. *Psychiatry & Clinical Neurosciences*. 2006;60:182. | > 2 weeks between EPDS and diagnostic interview |
| Uguz F, Akman C, Sahingoz M, Kaya N, Kucur R. One year follow-up of post-partum-onset depression: the role of depressive symptom severity and personality disorders. *Journal of Psychosomatic Obstetrics & Gynecology*. 2009;30:141. | Sample selected for known distress, mental health diagnosis, or psychiatric setting |
| Usuda K, Nishi D, Okazaki E, Makino M, Sano Y. Optimal cut‐off score of the Edinburgh Postnatal Depression Scale for major depressive episode during pregnancy in Japan. *Psychiatry and Clinical Neurosciences*. 2017;71:836-42. | Sample selected for known distress, mental health diagnosis, or psychiatric setting |
| Uwakwe R, Okonkwo JE. Affective (depressive) morbidity in puerperal Nigerian women: validation of the Edinburgh Postnatal Depression Scale. *Acta Psychiatrica Scandinavica*. 2003;107:251. | No validated interview to assess major depression |
| Van Der Zee-Van AI, Boere-Boonekamp MM, Groothuis-Oudshoorn CG, IJzerman MJ, Haasnoot-Smallegange RM, Reijneveld SA. Post-up study: postpartum depression screening in well-child care and maternal outcomes. *Pediatrics*. 2017;140:e20170110.1. | > 2 weeks between EPDS and diagnostic interview |
| Varela P, Spyropoulou AC, Kalogerakis Z, Vousoura E, Moraitou M, Zervas IM. Association between gestational diabetes and perinatal depressive symptoms: evidence from a Greek cohort study. *Primary Health Care Research & Development*. 2017;18:441-7. | No major depression |
| Venkatesh KK, Zlotnick C, Triche EW, Ware C, Phipps MG. Accuracy of brief screening tools for identifying postpartum depression among adolescent mothers. *Pediatrics*. 2014;133:e45. | No adults |
| Venter MD, Smets J, Raes F, Wouters K, Franck E, Hanssens M, Jacquemyn Y, Sabbe BGC, Eede FVD. Impact of childhood trauma on postpartum depression: A prospective study. *Archives of Women's Mental Health*. 2016;19:337. | No major depression |
| Verkerk GJ, Denollet J, Van Heck GL, Van Son MJ, Pop VJ. Personality factors as determinants of depression in postpartum women: a prospective 1-year follow-up study. *Psychosomatic Medicine*. 2005;67:632. | No validated interview to assess major depression |
| Verkerk GJM, Pop VJM, Van Son MJM, Van Heck GL. Prediction of depression in the postpartum period: A longitudinal follow-up study in high-risk and low-risk women. *Journal of Affective Disorders*. 2003;77:159. | > 2 weeks between EPDS and diagnostic interview |
| Viktorin A, Meltzer-Brody S, Kuja-Halkola R, Sullivan PF, Landen M, Lichtenstein P, Magnusson PK. Heritability of Perinatal Depression and Genetic Overlap With Nonperinatal Depression. *American Journal of Psychiatry*. 2016;173:158. | No EPDS |
| Wang Y, Guo X, Lau Y, Chan KS, Yin L, Chen J. Psychometric evaluation of the Mainland Chinese version of the Edinburgh Postnatal Depression Scale. *International Journal of Nursing Studies*. 2009;46:813. | Could not determine eligibility^a^ |
| Warner R, Appleby L, Whitton A, Faragher B. Attitudes toward motherhood in postnatal depression: development of the Maternal Attitudes Questionnaire. *Journal of Psychosomatic Research*. 1997;43:351. | Sample selected for known distress, mental health diagnosis, or psychiatric setting |
| Warnock FF, Bakeman R, Shearer K, Misri S, Oberlander T. Caregiving behavior and interactions of prenatally depressed mothers (antidepressant-treated and non-antidepressant-treated) during newborn acute pain. *Infant Mental Health Journal*. 2009;30:384. | Could not determine eligibility^a^ |
| Wenz-Gross M, Weinreb L, Upshur C. Screening for post-traumatic stress disorder in prenatal care: Prevalence and characteristics in a low-income population. *Maternal and Child Health Journal*. 2016;20:1995-2002. | No major depression |
| Weobong B, Akpalu B, Doku V, Agyei SO, Hurt L, Kirkwood B, Prince M. The comparative validity of screening scales for postnatal common mental disorder in Kintampo, Ghana. *Journal of Affective Disorders*. 2009;113:109. | No validated interview to assess major depression |
| Werrett J, Clifford C. Validation of the Punjabi version of the Edinburgh postnatal depression scale (EPDS). *International Journal of Nursing Studies*. 2006;43:227. | No major depression |
| Wickberg B, Hwang CP. Counselling of postnatal depression: a controlled study on a population based Swedish sample. *Journal of Affective Disorders*. 1996;39:209. | No validated interview to assess major depression |
| Wickberg B, Hwang CP. The Edinburgh Postnatal Depression Scale: validation on a Swedish community sample. *Acta Psychiatrica Scandinavica*. 1996;94:181. | No validated interview to assess major depression |
| Williams JA, Romero VC, Clinton CM, Vazquez DM, Marcus SM, Chilimigras JL, Hamilton SE, Allbaugh LJ, Vahratian AM, Schrader RM, Mozurkewich EL. Vitamin D levels and perinatal depressive symptoms in women at risk: a secondary analysis of the mothers, omega-3, and mental health study. *BMC Pregnancy and Childbirth*. 2016;16:203. | Sample selected for known distress, mental health diagnosis, or psychiatric setting |
| Wisner KL, Sit DK, McShea M, Luther JF, Eng HF, Dills JL, Moses-Kolko EL, Wisniewski SR. Telephone-Based Depression Care Management for Postpartum Women: A Randomized Controlled Trial. *The Journal of Clinical Psychiatry*. 2017;78:1369-75. | Sample selected for known distress, mental health diagnosis, or psychiatric setting |
| Wu M, Li X, Feng B, Wu H, Qiu C, Zhang W. Correlation between sleep quality of third-trimester pregnancy and postpartum depression. *Medical Science Monitor*. 2014;20:2740. | Could not determine eligibility^a^ |
| Yamashita H, Yoshida K, Nakano H, Tashiro N. Postnatal depression in Japanese women. Detecting the early onset of postnatal depression by closely monitoring the postpartum mood. *Journal of Affective Disorders*. 2000;58:145. | No validated interview to assess major depression |
| Yelland C, Girke T, Tottman C, Williams AS. Clinical characteristics and mental health outcomes for women admitted to an Australian Mother–Baby Unit: a focus on borderline personality disorder and emotional dysregulation? *Australasian Psychiatry*. 2015;23:683-7. | No validated interview to assess major depression |
| Yonkers KA, Ramin SM, Rush AJ, Navarrete CA, Carmody T, March D, Heartwell SF, Leveno KJ. Onset and persistence of postpartum depression in an inner-city maternal health clinic system. *American Journal of Psychiatry*. 2001;158:1856. | Sample selected for known distress, mental health diagnosis, or psychiatric setting |
| Yoshida K, Yamashita H, Ueda M, Tashiro N. Postnatal depression in Japanese mothers and the reconsideration of 'Satogaeri bunben'. *Pediatrics International*. 2001;43:189. | No validated interview to assess major depression |
| Zammit S, Thomas K, Thompson A, Horwood J, Menezes P, Gunnell D, Hollis C, Wolke D, Lewis G, Harrison G. Maternal tobacco, cannabis and alcohol use during pregnancy and risk of adolescent psychotic symptoms in offspring. *The British Journal of Psychiatry*. 2009;195:294. | No pregnant or postpartum women |
| Zelkowitz P, Milet TH. Postpartum psychiatric disorders: Their relationship to psychological adjustment and marital satisfaction in the spouses. *Journal of Abnormal Psychology*. 1996;105:281. | Sample selected for known distress, mental health diagnosis, or psychiatric setting |
| Zlotnick C, Capezza NM, Parker D. An interpersonally based intervention for low-income pregnant women with intimate partner violence: A pilot study. *Archives of Women's Mental Health*. 2011;14:55. | Sample selected for known distress, mental health diagnosis, or psychiatric setting |
| Zubaran C, Foresti K, Schumacher MV, Amoretti AL, Thorell MR, Muller LC. The correlation between postpartum depression and health status. *Maternal & Child Health Journal*. 2010;14:751. | > 2 weeks between EPDS and diagnostic interview |

^a^It was not possible to determine eligibility based on the published report, and we were not able to obtain clarification from authors despite multiple attempts.

**Supplementary Table S2.** Characteristics of included primary studies (N = 41).

| **First Author, Year** | **Country** | **Recruited Population** | **Diagnostic Interview** | **Classification System** | **Total N** | **Major Depression** |
| --- | --- | --- | --- | --- | --- | --- |
|  |  |  |  |  |  | **N (%)** |
| **Semi-structured Interviews** | | | | | | |
| **Barnes, 2009^1^** | UK | Socially disadvantaged mothers at 2 months postpartum | SCID | DSM-III-R | 347 | 25 (7) |
| **Beck, 2001^2^** | USA | Postpartum mothers | SCID | DSM-IV | 150 | 18 (12) |
| **de Figueiredo, 2015^3^** | Brazil | Postpartum women enrolled in prenatal care outpatient services in a Brazilian city | SCID | DSM_IV | 241 | 94 (39) |
| **Green, 2018^4^** | Kenya | Pregnant and postpartum women receiving maternity services | SCID | DSM-V | 161 | 7 (4) |
| **Helle, 2015^5^** | Germany | Mothers with very low birthweight and normal weight infants between 4 and 6 weeks postpartum | SCID | DSM-IV | 224 | 12 (5) |
| **Howard, 2018^6^** | UK | Pregnant women recruited from an inner-city London maternity service | SCID | DSM-IV | 527 | 130 (25) |
| **Ing, 2017^7^** | Thailand | Postpartum migrant and refugee women | SCID | DSM-IV | 625 | 5 (1) |
| **Kettunen, 2017^8a^** | Finland | Postpartum women recruited from antenatal clinics | SCID | DSM-IV | 134 | 65 (49) |
| **Leonardou, 2009^9^** | Greece | Postpartum women recruited from private and public maternity wards on their second day postpartum | SCID | DSM-III-R | 81 | 4 (5) |
| **Nakić Radoš, 2013^10^** | Croatia | Women between 6 and 8 weeks postpartum | SCID | DSM-IV-TR | 272 | 10 (4) |
| **Pawlby, 2008^11^** | UK | Women at 12 months postpartum | CIS | ICD-9 | 190 | 34 (18) |
| **Phillips, 2009^12^** | Australia | Postpartum mothers with unsettled infants | SCID | DSM-IV | 158 | 42 (27) |
| **Prenoveau, 2013^13^** | UK | Postpartum women at 10 months recruited from mixed health centres | SCID | DSM-IV | 219 | 20 (9) |
| **Rochat, 2013^14^** | South Africa | Women recruited from their antenatal appointment at a primary health care clinic between 26 and 34 weeks of pregnancy | SCID | DSM-IV | 104 | 50 (48) |
| **Smith-Nielsen, 2018^15^** | Denmark | Postpartum women | SCID | DSM-V | 320 | 118 (37) |
| **Stewart, 2013^16^** | Malawi | Pregnant women attending an antenatal clinic in rural Malawi | SCID | DSM-IV | 186 | 34 (18) |
| **Tandon, 2012^17^** | USA | Pregnant and postpartum women enrolled in home visitation programs | SCID | DSM IV | 89 | 25 (28) |
| **Tendais, 2014^18^** | Portugal | Pregnant women recruited in an obstetrics outpatient unit | SCID | DSM-IV | 141 | 18 (13) |
| **Tissot, 2015^19^** | Switzerland | Women at 3 months postpartum | DIGS | DSM-IV | 65 | 4 (6) |
| **Töreki, 2013^20^** | Hungary | Women at 12 weeks antenatal | SCID | DSM-IV | 219 | 7 (3) |
| **Töreki, 2014^21^** | Hungary | Women between 6 and 8 weeks postpartum | SCID | DSM-IV | 265 | 8 (3) |
| **Tran, 2011^22^** | Vietnam | Pregnant and postpartum Vietnamese women recruited from the commune health centre | SCID | DSM-IV | 359 | 52 (14) |
| **Turner, 2009^23^** | Italy | Women from a regional epilepsy center in Italy between 5 and 8 weeks postpartum | SCID | DSM-IV-TR | 29 | 2 (7) |
| **Vega-Dienstmaier, 2002^24^** | Peru | Women up to 12 months postpartum | SCID | DSM-IV | 306 | 19 (6) |
| **Fully Structured Interviews** | | | | | | |
| **Felice, 2004^25^** | Malta | Pregnant women attending an antenatal clinic | CIS-R | ICD-10 | 226 | 32 (14) |
| **Fisher, 2010^26^** | Australia | Postpartum women recruited in Australian maternal and child health centres at 6 months postpartum | CIDI | DSM-IV | 192 | 1 (1)^c^ |
| **Rowe, 2008^27^** | Australia | English speaking women admitted with their up to 1-year-old infants to private parenting centers | CIDI | DSM-IV | 137 | 25 (18) |
| **Yonkers, 2014^28^** | USA | Women at 17 weeks’ gestation | CIDI | DSM-IV | 2634 | 170 (6) |
| **Mini International Neuropsychiatric Interviews (MINI)** | | | | | | |
| **Alvarado, 2015^29^** | Chile | Pregnant women up to 28 weeks’ gestation | MINI | DSM-IV | 111 | 38 (34) |
| **Chorwe-Sungani, 2018^30^** | Malawi | Pregnant women recruited from antenatal clinics | MINI | DSM-IV | 96 | 25 (26) |
| **Couto, 2015^31^** | Brazil | Women in their second trimester of pregnancy recruited at antenatal care in a public hospital | MINI | DSM-IV-TR | 173 | 36 (21) |
| **Comasco, 2016^32^** | Sweden | Pregnant women | MINI | DSM-IV | 220 | 18 (8) |
| **Eapen, 2013^33^** | Australia | Women attending an antenatal clinic in Sydney | MINI | DSM-IV | 131 | 26 (20) |
| **Fernandes, 2011^34^** | India | Rural women in their third trimester | MINI | DSM-IV | 133 | 27 (20) |
| **Figueira, 2009^35^** | Brazil | Postpartum mothers recruited from hospitalization records | MINI | DSM-IV | 239 | 18 (8) |
| **Khalifa, 2015^36^** | Sudan | Women at 3 months postpartum | MINI | ICD-10 | 40 | 18 (45) |
| **Martinez, 2016^37^** | Chile | Postpartum mothers participating in a child health monitoring program | MINI | DSM-IV | 298 | 63 (21) |
| **Roomruangwong, 2016^38^** | Thailand | Pregnant women at the end of their term | MINI | DSM-IV-TR | 126 | 1 (1) |
| **Su, 2007^39^** | Taiwan | Women in their second and third trimesters | MINI | DSM-IV | 185 | 23 (12) |
| **Usuda, 2016^40^** | Japan | Pregnant women between 12-24 weeks of gestation recruited at maternity hospital in Japan | MINI | DSM-IV | 177 | 2 (1) |
| **van Heyningen, 2018^41^** | South Africa | Pregnant women recruited from primary care antenatal clinics | MINI | DSM-IV | 376 | 81 (22) |

**Abbreviations**: CIDI: Composite International Diagnostic Interview; CIS: Clinical Interview Schedule; CIS-R: Clinical Interview Schedule Revised; DIGS: Diagnostic Interview for Genetic Studies; DSM: Diagnostic and Statistical Manual of Mental Disorders; ICD: International Classification of Diseases; MINI: Mini Neuropsychiatric Diagnostic Interview; SCID: Structured Clinical Interview for DSM Disorders; UK: United Kingdom; USA: United States of America.

^a^The primary study used a case-control design, but was unable to provide statistical weights to reflect sampling procedures.

^b^This study was not retrieved at the time of electronic database search.

^c^This case was excluded from the bivariate random-effects meta-analyses.

**Supplementary Table S3.** Characteristics of eligible primary studies that did not provide data for the present study (N = 42).

| **First Author, Year** | **Country** | **Recruited Population** | **Diagnostic Interview** | **Total N** | **Major Depression** | **Reason for not contributing data** |
| --- | --- | --- | --- | --- | --- | --- |
|  |  |  |  |  | **N (%)** |  |
| **Semi-structured Interviews** | | | | | | |
| **Aceti, 2012^42^** | Italy | Pregnant women in the third trimester | SCID | 44 | 22 (50) | The dataset didn’t provide item-level data for EPDS items |
| **Aydin, 2004^43^** | Turkey | Women within their first postpartum year attending primary health care clinics in the province of Erzurum | SCID | 341 | 34 (10) | The author indicated that the data no longer exist |
| **Banti, 2011^44^** | Italy | Pregnant women presenting to the local health service in the region of Tuscany between 12 and 15 weeks’ gestation | SCID | 1066 | NR | The author initially responded but did not provide data and did not respond to further emails |
| **Bavle, 2016^45^** | India | Pregnant women recruited from an outpatient obstetrics department in a tertiary care hospital | SCID | 318 | 6 (2) | The dataset didn’t provide item-level data for EPDS items |
| **Brodey, 2016^46^** | USA | Pregnant women recruited from private obstetrics clinics in Atlanta, Georgia and Tulsa, Oklahoma as well as women within 150 days postpartum | SCID | 879 | NR | The author indicated that she/he was not willing to share data |
| **Bunevicius, 2009^47^** | Lithuania | Pregnant women 12 to 16 weeks pregnant attending an obstetric clinic | SCID | 230 | 12 (5) | The dataset didn’t provide item-level data for EPDS items |
| **Chibanda, 2010^48^** | Zimbabwe | HIV-infected and uninfected women attending two primary care clinics in Chitungwiza six weeks postpartum | SCID | 210 | NR | The author indicated that the data no longer exist |
| **Chaudron, 2010^49^** | USA | Postpartum women recruited from Well-Child Care visits with infants 0-14 months of age | SCID | 187 | 70 (37) | The dataset didn’t provide item-level data for EPDS items |
| **Crotty, 2004^50^** | Ireland | Women between 6 and 8 weeks postpartum | SCAN | 113 | 48 (42) | The author provided a dataset but could not clarify distripencies between the data and the published study |
| **Garcia-Esteve, 2003^51^** | Spain | Women at 6 weeks postpartum | SCID | 334 | 36 (11) | The dataset didn’t provide item-level data for EPDS items |
| **Gausia, 2007^52^** | Bangladesh | Women 6 to 8 weeks postpartum attending an urban childhood immunization clinic in Bangladesh | SCID | 100 | 3 (3) | The author provided a dataset but could not distinguish between major and minor depression cases |
| **Giardinelli, 2012^53^** | Italy | Women between 28 and 32 weeks pregnant recruited from a obstetric course in Florence | SCID | 588 | 28 (5) | The dataset didn’t provide item-level data for EPDS items |
| **Gorman, 2004^54^** | France, Ireland, Italy, USA, UK, Portugal, Austria, Switzerland | Women in their third trimester of pregnancy from 10 sites in 8 countries | SCID | 289 | 10 (3) | The author indicated that too much work was involved and she/he did not have time |
| **Hickey, 1997^55^** | Australia | Postpartum women recruited in the hospital after delivery | SCID | 72 | 31 (43) | The dataset didn’t provide item-level data for EPDS items |
| **Li, 2011^56^** | China | Women between 2 and 12 weeks postpartum recruited from postnatal clinics of the three regional public hospitals in Changsha, China | SCID | 387 | 24 (6) | The author never replied despite multiple attempts to contact |
| **Marsay, 2017^57^** | South Africa | Pregnant women between 22 and 28 weeks’ gestation | SCID | 145 | 16 (11) | The dataset didn’t provide item-level data for EPDS items |
| **Moses-Kolko, 2012^58^** | USA | Postpartum women within 16 weeks of delivery | SCID | 33 | 13 (39) | The author never replied despite multiple attempts to contact |
| **Navarro, 2007^59^** | Spain | Women presenting for postpartum care at 6 weeks | SCID | 401 | 84 (21) | The dataset didn’t provide item-level data for EPDS items |
| **Priest, 2003^60^** | Australia | Women at 2 months postpartum who delivered healthy term infants | SADS | 292 | NR | The author initially responded but did not provide data and did not respond to further emails |
| **Robertson-Blackmore, 2013^61^** | USA | Women at 18 weeks’ gestation | SCID | 358 | 29 (8) | The dataset didn’t provide item-level data for EPDS items |
| **Siu, 2012^62^** | China | Postpartum women | SCID | 805 | 126 (16) | The dataset didn’t provide item-level data for EPDS items |
| **Stuebe, 2013^63^** | USA | Women in the third trimester of a singleton pregnancy who intended to breastfeed for at least 3 months | SCID | 47 | 8 (17) | The author indicated that she/he was not willing to share data |
| **Tungchama, 2017^64^** | Nigeria | Postpartum women recruited from welfare clinics | SCID | 147 | 64 (44) | The dataset didn’t provide item-level data for EPDS items |
| **Fully Structured Interviews** | | | | | | |
| **Barnett, 1999^65^** | Australia | Pregnant women during their second trimester from four antenatal clinics in South-Western Sydney | DIS | 316 | 21 (7) | The author never replied despite multiple attempts to contact |
| **Bergink, 2011^66^** | The Netherlands | Pregnant women at 12 weeks’ gestation from 5 community midwifery practices in and around the city of Eindhoven | CIDI | 845 | 47 (6) | The author indicated that the data no longer exist |
| **Mahmud, 2003^67^** | Malaysia | Women between 4 and 12 weeks postpartum attending a health clinic in Kedah | CIDI | 64 | 9 (14) | The author indicated that the data no longer exist |
| **Matthey, 2001^68^** | Australia | Women between 6 and 7 weeks postpartum who attended an evening preparation for parenthood class with their partners in South West Sydney | DIS | 230 | 11 (5) | The author indicated that too much work was involved and she/he did not have time |
| **O’Brien, 2004^69^** | UK | Mother of children with serial weights that crossed 2 major centiles on | CIS-R | 216 | 31 (14) | The author provided a dataset, but could not clarify distripencies between the data and the published study |
|  |  | standardized growth charts or fell below the second centile. |  |  |  |  |
| **Mini International Neuropsychiatric Interviews (MINI)** | | | | | | |
| **Adewuya, 2006^70^** | Nigeria | Women between 32 and 36 weeks pregnant recruited from the antenatal clinics in western Nigeria | MINI | 86 | 9 (10) | The author initially responded but did not provide data and did not respond to further emails |
| **Adouard, 2005^71^** | France | Women between 28 and 34 weeks’ gestation attending antenatal consultations for pregnancy complication in a major Parisian maternity facility | MINI | 60 | 15 (25) | The author indicated that the data no longer exist |
| **Agoub, 2005^72^** | Morocco | Postpartum women at their first postnatal visit 15 to 20 days after delivery | MINI | 144 | 27 (19) | The author never replied despite multiple attempts to contact |
| **Alvarado-Esquivel, 2006^73^** | Mexico | Women within 3 months postpartum | MINI | 91 | 10 (11) | The dataset didn’t provide item-level data for EPDS items |
| **Alvarado-Esquivel, 2016^74^** | Mexico | Pregnant women recruited at a public hospital | MINI | 184 | 12 (7) | The dataset didn’t provide item-level data for EPDS items |
| **Bakare, 2014^75^** | Nigeria | Postpartum women | MINI | 405 | 62 (15) | The dataset didn’t provide item-level data for EPDS items |
| **Benvenuti, 1999^76^** | Italy | Women between 8 and 12 weeks postpartum in Florence’s metropolitan area | MINI | 113 | 18 (16) | The author initially responded but did not provide data and did not respond to further emails |
| **Berle, 2003^77^** | Norway | Women attending routine postnatal visits between 6 and 12 weeks postpartum | MINI | 100 | 27 (27) | The author indicated that too much work was involved and she/he did not have time |
| **Christl, 2013^78^** | Australia | Mothers with unsettled infants aged up to 12 months from a family care centre in Canterbury | MINI | 232 | 13 (6) | The author initially responded but did not provide data and did not respond to further emails |
| **Imbula, 2012^79^** | Democratic Republic of Congo | Women between 1 and 10 months postpartum recruited from 'well-baby' clinics | MINI | 117 | 29 (25) | The dataset didn’t provide item-level data for EPDS items |
| **Pedersen, 2016^80^** | USA | Euthyroid women between 35 to 36 weeks pregnant recruited from a public health obstetrics clinic | MINI | 199 | NR | The author indicated that too much work was involved and she/he did not have time |
| **Pinheiro, 2013^81^** | Brazil | Women between 32 and 36 weeks pregnant recruited from the antenatal clinics in western Nigeria | MINI | 207 | 27 (13) | The author initially responded but did not provide data and did not respond to further emails |
| **Thiagayson, 2013^82^** | Singapore | Inpatient high-risk pregnant women at 23 weeks or more of gestation | MINI | 200 | 22 (11) | The dataset didn’t provide item-level data for EPDS items |
| **van der Westhuizen, 2018^83^** | South Africa | Pregnant women between 20 and 28 weeks’ gestation | MINI | 662 | 31 (5) | The author's decision to contribute is still pending |

CIDI: Composite International Diagnostic Interview; CIS-R: Clinical Interview Schedule Revised; DIS: Diagnostic Interview Schedule; MINI: Mini International Neuropsychiatric Interview; NR: Not Reported; SADS: Schedule for Affective Disorders and Schizophrenia; SCAN: Schedule for Clinical Assessment in Neuropsychiatry; SCID: Structured Clinical Interview for DSM Disorders; UK: United Kingdom; USA: United States of America.

**Supplementary Table S4.** Comparison of sensitivity and specificity estimates between EPDS-9 and full EPDS across cutoffs ≥ 7 to ≥ 15 for studies that used different three reference standards.

| **Cut-off** | **EPDS-9^a,b^** | | | | **full EPDS^c,d,e,f^** | | | | **EPDS-9 – full EPDS** | | | |
| --- | --- | --- | --- | --- | --- | --- | --- | --- | --- | --- | --- | --- |
|  | **Sensitivity** | **95% CI** | **Specificity** | **95% CI** | **Sensitivity** | **95% CI** | **Specificity** | **95% CI** | **Sensitivity** | **95% CI** | **Specificity** | **95% CI** |
| **Semi-structured reference standard (N Studies = 24; N Participants = 5412; N major depression = 803)** | | | | | | | | | | | | |
| ≥ 7 | 0.94 | (0.89, 0.97) | 0.65 | (0.56, 0.73) | 0.94 | (0.89, 0.97) | 0.65 | (0.56, 0.73) | 0.00 | (-0.00, 0.00) | 0.00 | (0.00, 0.01) |
| ≥ 8 | 0.89 | (0.82, 0.93) | 0.72 | (0.63, 0.79) | 0.90 | (0.83, 0.94) | 0.71 | (0.63, 0.79) | -0.01 | (-0.03, 0.00) | 0.01 | (0.00, 0.01) |
| ≥ 9 | 0.86 | (0.79, 0.91) | 0.78 | (0.71, 0.84) | 0.87 | (0.79, 0.92) | 0.77 | (0.70, 0.84) | -0.01 | (-0.03, 0.00) | 0.01 | (0.00, 0.01) |
| ≥ 10 | 0.81 | (0.72, 0.87) | 0.84 | (0.78, 0.88) | 0.82 | (0.74, 0.88) | 0.83 | (0.77, 0.88) | -0.01 | (-0.04, -0.00) | 0.01 | (0.00, 0.01) |
| ≥ 11 | 0.76 | (0.66, 0.84) | 0.88 | (0.83, 0.92) | 0.78 | (0.69, 0.85) | 0.88 | (0.83, 0.91) | -0.02 | (-0.04, -0.00) | 0.00 | (0.00, 0.01) |
| ≥ 12 | 0.69 | (0.58, 0.78) | 0.92 | (0.88, 0.95) | 0.71 | (0.61, 0.80) | 0.92 | (0.88, 0.94) | -0.02 | (-0.04, -0.00) | 0.00 | (0.00, 0.01) |
| ≥ 13 | 0.60 | (0.48, 0.72) | 0.95 | (0.92, 0.96) | 0.65 | (0.53, 0.75) | 0.94 | (0.91, 0.96) | -0.05 | (-0.10, -0.01) | 0.01 | (0.00, 0.01) |
| ≥ 14 | 0.51 | (0.40, 0.62) | 0.96 | (0.94, 0.98) | 0.56 | (0.44, 0.67) | 0.96 | (0.93, 0.98) | -0.05 | (-0.09, -0.02) | 0.00 | (0.00, 0.01) |
| ≥ 15^c^ | 0.47 | (0.37, 0.57) | 0.98 | (0.96, 0.99) | 0.50 | (0.40, 0.60) | 0.97 | (0.95, 0.98) | -0.03 | (-0.07, -0.01) | 0.01 | (0.00, 0.01) |
| **Fully structured reference standard (MINI excluded) (N Studies = 4; N Participants = 3188; N major depression = 227)^g^** | | | | | | | | | | | | |
| ≥ 7 | 0.95 | (0.71-0.99) | 0.57 | (0.36-0.76) | 0.95 | (0.71-0.99) | 0.57 | (0.36-0.76) | 0.00 | (-0.00, 0.00) | 0.00 | (0.00, 0.01) |
| ≥ 8 | 0.95 | (0.70-0.99) | 0.63 | (0.41-0.81) | 0.95 | (0.70-0.99) | 0.62 | (0.41-0.80) | 0.00 | (-0.02, 0.01) | 0.01 | (0.00, 0.02) |
| ≥ 9 | 0.95 | (0.64-1.00) | 0.71 | (0.51-0.86) | 0.95 | (0.64-1.00) | 0.71 | (0.50-0.85) | 0.00 | (-0.02, 0.01) | 0.00 | (0.00, 0.02) |
| ≥ 10 | 0.91 | (0.64-0.98) | 0.78 | (0.57-0.91) | 0.93 | (0.64-0.99) | 0.78 | (0.57-0.90) | -0.02 | (-0.09, 0.05) | 0.00 | (-0.00, 0.01) |
| ≥ 11 | 0.90 | (0.58-0.98) | 0.83 | (0.63-0.94) | 0.90 | (0.58-0.98) | 0.83 | (0.62-0.94) | 0.00 | (-0.01, 0.02) | 0.00 | (0.00, 0.01) |
| ≥ 12 | 0.80 | (0.55-0.93) | 0.86 | (0.71-0.94) | 0.81 | (0.56-0.94) | 0.86 | (0.70-0.94) | -0.01 | (-0.06, 0.04) | 0.00 | (-0.00, 0.01) |
| ≥ 13 | 0.79 | (0.49-0.94) | 0.91 | (0.76-0.97) | 0.79 | (0.50-0.94) | 0.90 | (0.75-0.96) | 0.00 | (-0.14, 0.25) | 0.01 | (0.00, 0.03) |
| ≥ 14 | 0.76 | (0.40-0.94) | 0.94 | (0.82-0.98) | 0.77 | (0.43-0.94) | 0.93 | (0.82-0.98) | -0.01 | (-0.21, 0.04) | 0.01 | (-0.00, 0.03) |
| ≥ 15 | 0.63 | (0.34-0.84) | 0.96 | (0.87-0.99) | 0.66 | (0.37-0.87) | 0.95 | (0.86-0.99) | -0.03 | (-0.15, 0.01) | 0.01 | (-0.00, 0.01) |
| **MINI reference standard (N Studies = 13; N Participants = 2305; N major depression = 376)** | | | | | | | | | | | | |
| ≥ 7 | 0.96 | (0.87, 0.99) | 0.62 | (0.52, 0.71) | 0.96 | (0.87, 0.99) | 0.62 | (0.51-0.71) | 0.00 | (-0.00, 0.00) | 0.00 | (0.00, 0.01) |
| ≥ 8 | 0.91 | (0.83, 0.95) | 0.70 | (0.60, 0.78) | 0.91 | (0.84, 0.96) | 0.69 | (0.59-0.78) | 0.00 | (-0.02, 0.00) | 0.01 | (0.00, 0.02) |
| ≥ 9^d^ | 0.87 | (0.78, 0.93) | 0.76 | (0.66, 0.84) | 0.88 | (0.79, 0.94) | 0.76 | (0.66-0.83) | -0.01 | (-0.04, 0.00) | 0.00 | (0.00, 0.01) |
| ≥ 10 | 0.83 | (0.70, 0.91) | 0.81 | (0.72, 0.87) | 0.84 | (0.71, 0.92) | 0.80 | (0.72, 0.86) | -0.01 | (-0.05, 0.00) | 0.01 | (0.00, 0.01) |
| ≥ 11 | 0.78 | (0.66, 0.87) | 0.85 | (0.79, 0.90) | 0.82 | (0.68, 0.91) | 0.85 | (0.78, 0.90) | -0.04 | (-0.09, -0.00) | 0.00 | (-0.00, 0.01) |
| ≥ 12 | 0.71 | (0.56, 0.82) | 0.90 | (0.83, 0.94) | 0.75 | (0.58, 0.86) | 0.89 | (0.82, 0.93) | -0.04 | (-0.13, 0.00) | 0.01 | (0.00, 0.02) |
| ≥ 13 | 0.66 | (0.51, 0.79) | 0.92 | (0.87, 0.96) | 0.71 | (0.54, 0.84) | 0.92 | (0.86, 0.95) | -0.05 | (-0.10, -0.01) | 0.00 | (0.00, 0.02) |
| ≥ 14^a,e^ | 0.55 | (0.41, 0.67) | 0.95 | (0.91, 0.97) | 0.61 | (0.44-0.75) | 0.94 | (0.90, 0.97) | -0.06 | (-0.11, -0.01) | 0.01 | (0.00, 0.02) |
| ≥ 15^b,f^ | 0.45 | (0.33, 0.59) | 0.96 | (0.93, 0.98) | 0.51 | (0.37, 0.65) | 0.95 | (0.91, 0.98) | -0.06 | (-0.12, -0.01) | 0.01 | (0.00, 0.02) |

^a^For EPDS-9 cut-off 14, among studies that used MINI reference standard, the default and bobyqa optimizers in glmer all failed to converge, thus nlminb was used instead.

^b^For EPDS-9 cut-off 15, among studies that used MINI reference standard, the default optimizer in glmer failed to converge, thus bobyqa was used instead.

^c^For full EPDS cut-off 15, among studies that used semi-structured reference standard, the default optimizer in glmer failed to converge, thus bobyqa was used instead.

^d^For full EPDS cut-off 9, among studies that used MINI reference standard, the default optimizer in glmer failed to converge, thus bobyqa was used instead.

^e^For full EPDS cut-off 14, among studies that used MINI reference standard, the default optimizer in glmer failed to converge, thus bobyqa was used instead.

^f^For full EPDS cut-off 15, among studies that used MINI reference standard, the default optimizer in glmer failed to converge, thus bobyqa was used instead.

^g^For fully structured reference standard (MINI excluded), the bivariate model was modified by setting the correlation between random effects to zero and the participant with major depression from the study was excluded, which had only one participant with major depression. Number of studies=3 for sensitivity and 4 for specificity. About the differences of the EPDS-9 and full EPDS with cut-offs ≥ 7 to ≥ 15, 996 out of 1000 iterations in R software ran successfully, which provided reliable evidence.

CI: confidence interval; EPDS: Edinburgh Postnatal Depression Scale.

**Supplementary Material References**

Provided Item-level Individual Participant Data

1. Barnes JS, Senior R, MacPherson K. The utility of volunteer home‐visiting support to prevent maternal depression in the first year of life. *Child Care Health Dev*. 2009;35:807-16.
2. Beck CT, Gable RK. Comparative analysis of the performance of the Postpartum Depression Screening Scale with two other depression instruments. *Nurs Res*. 2001;50:242-50.
3. de Figueiredo FP, Parada AP, Cardoso VC, Batista RF, da Silva AA, Barbieri MA, de Carvalho Cavalli R, Bettiol H, Del-Ben CM. Postpartum depression screening by telephone: a good alternative for public health and research. *Arch Womens Ment Health*. 2015;18:547-53.
4. Green EP, Tuli H, Kwobah E, Menya D, Chesire I, Schmidt C. Developing and validating a perinatal depression screening tool in Kenya blending Western criteria with local idioms: A mixed methods study. *J Affect Disord*. 2018;228:49.
5. Helle N, Barkmann C, Bartz-Seel J, Diehl T, Ehrhardt S, Hendel A, Nestoriuc Y, Schulte-Markwort M, Von Der Wense A, Bindt C. Very low birth-weight as a risk factor for postpartum depression four to six weeks postbirth in mothers and fathers: Cross-sectional results from a controlled multicentre cohort study. *J Affect Disord*. 2015;180:154-61.
6. Howard LM, Ryan EG, Trevillion K, Anderson F, Bick D, Bye A, et al. Accuracy of the Whooley questions and the Edinburgh Postnatal Depression Scale in identifying depression and other mental disorders in early pregnancy. *Br J Psychiatry*. 2018;212:50-56.
7. Ing H, Fellmeth G, White J, Stein A, Simpson JA, McGready R. Validation of the Edinburgh Postnatal Depression Scale (EPDS) on the Thai-Myanmar border. *Trop Doct.* 2017;47:339.
8. Kettunen P, Hintikka J. Psychosocial risk factors and treatment of new onset and recurrent depression during the post-partum period. *Nord J Psychiatry*. 2017;71:355.
9. Leonardou AA, Zervas YM, Papageorgiou CC, Marks MN, Tsartsara EC, Antsaklis A, Christodoulou GN, Soldatos CR. Validation of the Edinburgh Postnatal Depression Scale and prevalence of postnatal depression at two months postpartum in a sample of Greek mothers. *J Reprod Infant Psychol*. 2009;27:28-39.
10. Nakić Radoš S, Tadinac M, Herman R. Validation study of the Croatian version of the Edinburgh Postnatal Depression Scale (EPDS). *Suvrem Psihol*. 2013;16:203-18.
11. Pawlby S, Sharp D, Hay D, O'Keane V. Postnatal depression and child outcome at 11 years: the importance of accurate diagnosis. *J Affect Disord*. 2008;107:241-5.
12. Phillips J, Charles M, Sharpe L, Matthey S. Validation of the subscales of the Edinburgh Postnatal Depression Scale in a sample of women with unsettled infants. *J Affect Disord.* 2009;118:101-12.
13. Prenoveau J, Craske M, Counsell N, West V, Davies B, Cooper P, Rapa E, Stein A. Postpartum GAD is a risk factor for postpartum MDD: the course and longitudinal relationships of postpartum GAD and MDD. *Depress Anxiety*. 2013;30:506-14.
14. Rochat TJ, Tomlinson M, Newell ML, Stein A. Detection of antenatal depression in rural HIV-affected populations with short and ultrashort versions of the Edinburgh Postnatal Depression Scale (EPDS). *Arch Womens Ment Health*. 2013;16:401-10.
15. Smith-Nielsen J, Matthey S, Lange T, Væver MS. Validation of the Edinburgh Postnatal Depression Scale against both DSM-5 and ICD-10 diagnostic criteria for depression. *BMC Psychiatry*. 2018;18:393.
16. Stewart RC, Umar E, Tomenson B, Creed F. Validation of screening tools for antenatal depression in Malawi—A comparison of the Edinburgh Postnatal Depression Scale and Self Reporting Questionnaire. *J Affect Disord*. 2013;150:1041-7.
17. Tandon SD, Cluxton-Keller F, Leis J, Le HN, Perry DF. A comparison of three screening tools to identify perinatal depression among low-income African American women. *J Affect Disord*. 2012;136:155-62.
18. Tendais I, Costa R, Conde A, Figueiredo B. Screening for depression and anxiety disorders from pregnancy to postpartum with the EPDS and STAI. *Span J Psychol*. 2014;17.
19. Tissot H, Favez N, Frascarolo-Moutinot F, Despland JN. Assessing postpartum depression: Evidences for the need of multiple methods. *Eur Rev Appl Psychol*. 2015;65:61-6.
20. Töreki A, Andó B, Keresztúri A, Sikovanyecz J, Dudas RB, Janka Z, Kozinszky Z, Pál A. The Edinburgh Postnatal Depression Scale: translation and antepartum validation for a Hungarian sample. *Midwifery*. 2013;29:308-15.
21. Töreki A, Andó B, Dudas RB, Dweik D, Janka Z, Kozinszky Z, Keresztúri A. Validation of the Edinburgh Postnatal Depression Scale as a screening tool for postpartum depression in a clinical sample in Hungary. *Midwifery*. 2014;30:911-8.
22. Tran TD, Tran T, La B, Lee D, Rosenthal D, Fisher J. Screening for perinatal common mental disorders in women in the north of Vietnam: a comparison of three psychometric instruments. *J Affect Disord*. 2011;133:281-93.
23. Turner K, Piazzini A, Franza A, Marconi AM, Canger R, Canevini MP. Epilepsy and postpartum depression. *Epilepsia.* 2009;50:24-7.
24. Vega-Dienstmaier JM, Mazzotti GS, Campos MS. Validation of a Spanish version of the Edinburgh postnatal depression scale. *Actas Esp Psiquiatr*. 2002;30:106-11.
25. Felice E, Saliba J, Grech V, Cox J. Prevalence rates and psychosocial characteristics associated with depression in pregnancy and postpartum in Maltese women. *J Affect Disord*. 2004;82:297-301.
26. Fisher JR, Wynter KH, Rowe HJ. Innovative psycho-educational program to prevent common postpartum mental disorders in primiparous women: a before and after controlled study*. BMC Public Health*. 2010;10:432.
27. Rowe HJ, Fisher JR, Loh WM. The Edinburgh Postnatal Depression Scale detects but does not distinguish anxiety disorders from depression in mothers of infants. *Arch Womens Ment Health*. 2008;11:103-8.
28. Yonkers KA, Smith MV, Forray A, Epperson CN, Costello D, Lin H, Belanger K. Pregnant women with posttraumatic stress disorder and risk of preterm birth. *JAMA Psychiatry*. 2014;71:897-904.
29. Alvarado R, Jadresic E, Guajardo V, Rojas G. First validation of a Spanish-translated version of the Edinburgh postnatal depression scale (EPDS) for use in pregnant women. A Chilean study. *Arch Womens Ment Health*. 2015;18:607-12.
30. Chorwe-Sungani G, Chipps J. Validity and utility of instruments for screening of depression in women attending antenatal clinics in Blantyre district in Malawi. *SAFP*. 2018;60:114.
31. Couto TC, Brancaglion MY, Cardoso MN, Protzner AB, Garcia FD, Nicolato R, Aguiar RA, Leite HV, Corrêa H. What is the best tool for screening antenatal depression? *J Affect Disord*. 2015;178:12-7.
32. Comasco E, Gulinello M, Hellgren C, Skalkidou A, Sylven S, Sundström-Poromaa I. Sleep duration, depression, and oxytocinergic genotype influence prepulse inhibition of the startle reflex in postpartum women. *Eur Neuropsychopharmacol*. 2016;26:767-76.
33. Eapen V, Johnston D, Apler A, Rees S, Silove DM. Adult separation anxiety during pregnancy and its relationship to depression and anxiety. *J Perinat Med.* 2013;41:159-63.
34. Fernandes MC, Srinivasan K, Stein AL, Menezes G, Sumithra RS, Ramchandani PG. Assessing prenatal depression in the rural developing world: a comparison of two screening measures. *Arch Womens Ment Health*. 2011;14:209-16.
35. Figueira P, Corrêa H, Malloy-Diniz L, Romano-Silva MA. Edinburgh Postnatal Depression Scale for screening in the public health system. *Rev Saude Publica*. 2009;43:79-84.
36. Khalifa DS, Glavin K, Bjertness E, Lien L. Postnatal depression among Sudanese women: prevalence and validation of the Edinburgh Postnatal Depression Scale at 3 months postpartum. *Int J Womens Health*. 2015;7:677.
37. Martinez P, Vohringer PA, Rojas G. Barriers to access to treatment for mothers with postpartum depression in primary health care centers: a predictive model. *Rev Lat Am Enfermagem*. 2016;24.
38. Roomruangwong C, Kanchanatawan B, Sirivichayakul S, Maes M. Antenatal depression and hematocrit levels as predictors of postpartum depression and anxiety symptoms. *Psychiatry Res.* 2016;238:211-7.
39. Su KP, Chiu TH, Huang CL, Ho M, Lee CC, Wu PL, Lin CY, Liau CH, Liao CC, Chiu WC, Pariante CM. Different cutoff points for different trimesters? The use of Edinburgh Postnatal Depression Scale and Beck Depression Inventory to screen for depression in pregnant Taiwanese women. *Gen Hosp Psychiatry*. 2007;29:436-41.
40. Usuda K, Nishi D, Makino M, Tachimori H, Matsuoka Y, Sano Y, Konishi T, Takeshima T. Prevalence and related factors of common mental disorders during pregnancy in Japan: a cross-sectional study. *Biopsychosoc Med*. 2016;10:17.
41. van Heyningen T, Honikman S, Tomlinson M, Field S, Myer L. Comparison of mental health screening tools for detecting antenatal depression and anxiety disorders in South African women. *PLoS One*. 2018;13.

Did not Provide Item-level Individual Participant Data

1. Aceti F, Aveni F, Baglioni V, Carluccio GM, Colosimo D, Giacchetti N, Marini I, Meuti V, Motta P, Zaccagni M, Biondi M. Perinatal and postpartum depression: from attachment to personality. A pilot study. *J Psychopathol.* 2012;18:328-34.
2. Aydin N, Inandi T, Yigit A, Hodoglugil NN. Validation of the Turkish version of the Edinburgh Postnatal Depression Scale among women within their first postpartum year. *Soc Psychiatry Psychiatr Epidemiol*. 2004;39:483.
3. Banti S, Mauri M, Oppo A, Borri C, Rambelli C, Ramacciotti D, Montagnani MS, Camilleri V, Cortopassi S, Rucci P, Cassano GB. From the third month of pregnancy to 1 year postpartum. Prevalence, incidence, recurrence, and new onset of depression. Results from the perinatal depression-research & screening unit study. *Compr Psychiatry*. 2011;52:343.
4. Bavle AD, Chandahalli AS, Phatak AS, Rangaiah N, Kuthandahalli SM, Nagendra PN. Antenatal depression in a tertiary care hospital. *Indian* *J Psychol Med*. 2016;38:31.
5. Brodey BB, Goodman SH, Baldasaro RE, Brooks-DeWeese A, Wilson ME, Brodey ISB, Doyle NM. Development of the Perinatal Depression Inventory (PDI)-14 using item response theory: a comparison of the BDI-II, EPDS, PDI, and PHQ-9. *Arch Womens Ment Health*. 2016;19:307.
6. Bunevicius A, Kusminskas L, Pop VJ, Pedersen CA, Bunevicius R. Screening for antenatal depression with the Edinburgh Depression Scale. *J Psychosom Obstet Gynaecol.* 2009;30:238-43.
7. Chibanda D, Mangezi W, Tshimanga M, Woelk G, Rusakaniko P, Stranix-Chibanda L, Midzi S, Maldonado Y, Shetty AK. Validation of the Edinburgh Postnatal Depression Scale among women in a high HIV prevalence area in urban Zimbabwe. *Arch Womens Ment Health*. 2010;13:201.
8. Chaudron LH, Szilagyi PG, Tang W, Anson E, Talbot NL, Wadkins HI, Tu X, Wisner KL. Accuracy of depression screening tools for identifying postpartum depression among urban mothers. *Pediatrics*. 2010:peds-2008.
9. Crotty F, Sheehan J. Prevalence and detection of postnatal depression in an Irish community sample. *Ir J Psychol Med*. 2004;21:117.
10. Garcia-Esteve L, Ascaso C, Ojuel J, Navarro P. Validation of the Edinburgh postnatal depression scale (EPDS) in Spanish mothers. *J Affect Disord*. 2003;75:71-6.
11. Gausia K, Fisher C, Algin S, Oosthuizen J. Validation of the Bangla version of the Edinburgh Postnatal Depression Scale for a Bangladeshi sample. *J Reprod Infant Psychol*. 2007;25:308.
12. Giardinelli L, Innocenti A, Benni L, Stefanini MC, Lino G, Lunardi C, Svelto V, Afshar S, Bovani R, Castellini G, Faravelli C. Depression and anxiety in perinatal period: prevalence and risk factors in an Italian sample. *Arch Womens Ment Health*. 2012;15:21-30.
13. Gorman LL, O'Hara MW, Figueiredo B, Hayes S, Jacquemain F, Kammerer MH, Klier CM, Rosi S, Seneviratne G, Sutter-Dallay AL, TCS-PND Group. Adaptation of the structured clinical interview for DSM-IV disorders for assessing depression in women during pregnancy and post-partum across countries and cultures. *Br J Psychiatry Suppl*. 2004;46:s17.
14. Hickey AR, Boyce PM, Ellwood D, Morris-Yates AD. Early discharge and risk for postnatal depression. *Med J Aust.* 1997;167:244-7.
15. Li L, Liu F, Zhang H, Wang L, Chen X. Chinese version of the Postpartum Depression Screening Scale: translation and validation. *Nurs Res*. 2011;60:231.
16. Marsay C, Manderson L, Subramaney U. Validation of the Whooley questions for antenatal depression and anxiety among low-income women in urban South Africa. *S Afr J Psychiatr.* 2017;23.
17. Moses-Kolko EL, Price JC, Wisner KL, Hanusa BH, Meltzer CC, Berga SL, Grace AA, di Scalea TL, Kaye WH, Becker C, Drevets WC. Postpartum and depression status are associated with lower [11 C] raclopride BP ND in Reproductive-Age Women. *Neuropsychopharmacology*. 2012;37:1422.
18. Navarro P, Ascaso C, Garcia-Esteve L, Aguado J, Torres A, Martín-Santos R. Postnatal psychiatric morbidity: a validation study of the GHQ-12 and the EPDS as screening tools. *Gen Hosp Psychiatry*. 2007;29:1-7.
19. Priest SR, Henderson J, Evans SF, Hagan R. Stress debriefing after childbirth: a randomised controlled trial. *Med J Aust*. 2003;178:542.
20. Robertson-Blackmore E, Putnam FW, Rubinow DR, Matthieu M, Hunn JE, Putnam KT, Moynihan JA, O'Connor TG. Antecedent trauma exposure and risk of depression in the perinatal period. *J Clin Psychiatry.* 2013;74:e942-8.
21. Siu BW, Leung SS, Ip P, Hung SF, O'Hara MW. Antenatal risk factors for postnatal depression: a prospective study of Chinese women at maternal and child health centres. *BMC Psychiatry*. 2012;12:22.
22. Stuebe AM, Grewen K, MeltzerBrody S. Association between maternal mood and oxytocin response to breastfeeding. *J Womens Health*. 2013;22:352.
23. Tungchama F, Piwuna C, Armiya'u A, Maigari Y, Davou F, Goar S, Umar M, Sadiq S, Ojih E, Uwakwe R. Independent socio-demographic and clinical correlates associated with the perception of quality of life of women with postpartum depressionin North-central, Nigeria. *Int J Psychiatry Clin Pract*. 2017;21:292.
24. Barnett B, Matthey S, Gyaneshwar R. Screening for postnatal depression in women of non-English speaking background. *Arch Womens Ment Health*. 1999;2:67.
25. Bergink V, Kooistra L, Lambregtse-van den Berg MP, Wijnen H, Bunevicius R, van Baar A,Pop V. Validation of the Edinburgh Depression Scale during pregnancy. *J Psychosom Res*. 2011;70:385.
26. Mahmud WM, Awang A, Mohamed MN. Revalidation of the Malay Version of the Edinburgh Postnatal Depression Scale (EPDS) Among Malay Postpartum Women Attending the Bakar Bata Health Center in Alor Setar, Kedah, North West Of Peninsular Malaysia. *Malays J Med Sci*. 2003;10:71.
27. Matthey S, Barnett B, Kavanagh DJ, Howie P. Validation of the Edinburgh Postnatal Depression Scale for men, and comparison of item endorsement with their partners. *J Affect Disord*. 2001;64:175.
28. O'Brien LM, Heycock EG, Hanna M, Jones PW, Cox JL. Postnatal depression and faltering growth: A community study. *Pediatrics*. 2004;113:1242.
29. Adewuya AO, Ola BA, Dada AO, Fasoto OO. Validation of the Edinburgh Postnatal Depression Scale as a screening tool for depression in late pregnancy among Nigerian women. *J Psychosom Obstet Gynaecol*. 2006;27:267.
30. Adouard F, Glangeaud-Freudenthal NM, Golse B. Validation of the Edinburgh postnatal depression scale (EPDS) in a sample of women with high-risk pregnancies in France. *Arch Womens Ment Health*. 2005;8:89.
31. Agoub M, Moussaoui D, Battas O. Prevalence of postpartum depression in a Moroccan sample. *Arch Womens Ment Health*. 2005;8:37.
32. Alvarado-Esquivel C, Sifuentes-Alvarez A, Salas-Martinez C, Martínez-García S. Validation of the Edinburgh Postpartum Depression Scale in a population of puerperal women in Mexico. *Clin Pract Epidemiol Ment Health*. 2006;2:33.
33. Alvarado-Esquivel C, Sifuentes-Alvarez A, Salas-Martinez C. Detection of mental disorders other than depression with the Edinburgh Postnatal Depression Scale in a sample of pregnant women in northern Mexico. *Ment Illn.* 2016;8.
34. Bakare MO, Okoye JO, Obindo JT. Introducing depression and developmental screenings into the National Programme on Immunization (NPI) in southeast Nigeria: an experimental cross-sectional assessment. *Gen Hosp Psychiatry*. 2014;36:105-12.
35. Benvenuti P, Ferrara M, Niccolai C, Valoriani V, Cox JL. The Edinburgh Postnatal Depression Scale: validation for an Italian sample. *J Affect Disord*. 1999;53:137.
36. Berle JO, Aarre TF, Mykletun A, Dahl AA, Holsten F. Screening for postnatal depression. Validation of the Norwegian version of the Edinburgh Postnatal Depression Scale, and assessment of risk factors for postnatal depression. *J Affect Disord*. 2003;76:151.
37. Christl B, Reilly N, Smith M, Sims D, Chavasse F, Austin MP. The mental health of mothers of unsettled infants: is there value in routine psychosocial assessment in this context? *Arch Womens Ment Health*. 2013;16:391.
38. Imbula BE, Okitundu EL, Mampunza SM. Postpartum depression in Kinshasa (DR Congo): prevalence and risk factors. *Med Sante Trop*. 2012;22:379-84.
39. Pedersen C, Leserman J, Garcia N, Stansbury M, Meltzer-Brody S, Johnson J. Late pregnancy thyroid-binding globulin predicts perinatal depression. *Psychoneuroendocrinology*. 2016;65:84.
40. Pinheiro RT, Coelho FM, Silva RA, Pinheiro KA, Oses JP, Quevedo Lde A, Souza LD, Jansen K, Zimmermann Peruzatto JM, Manfro GG, Giovenardi M, Almeida S, Lucion AB. Association of a serotonin transporter gene polymorphism (5-HTTLPR) and stressful life events with postpartum depressive symptoms: a population-based study. *J Psychosom Obstet Gynaecol*. 2013;34:29.
41. Thiagayson P, Krishnaswamy G, Lim ML, Sung SC, Haley CL, Fung DS, Allen Jr JC, Chen H. Depression and anxiety in Singaporean high-risk pregnancies—prevalence and screening. *Gen Hosp Psychiatry*. 2013;35:112-6.
42. van der Westhuizen C, Brittain K, Koen N, Maré K, Zar HJ, Stein DJ. Sensitivity and specificity of the SRQ-20 and the EPDS in diagnosing major depression ante-and postnatally in a south African birth cohort study. *Int J Ment Health Addict*. 2018;16:175-86.
